# Enzymatic synthesis of aromatic biobased polymers in green, low-boiling solvents

**DOI:** 10.1016/j.jbiotec.2024.10.003

**Published:** 2024-10-10

**Authors:** Thaís Fabiana Chan Salum, Daniel Day, James Sherwood, Alessandro Pellis, Thomas James Farmer

**Affiliations:** a University of York, Department of Chemistry, Green Chemistry Centre of Excellence, Heslington, York YO10 5DD, UK; b Embrapa Agroenergy, Parque Estação Biológica, Brasilia 70770-901, Brazil; c University of Genova, Department of Chemistry and Industrial Chemistry, via Dodecaneso 31, Genova 16146, Italy

**Keywords:** Pyridinedicarboxylic acids, Eucalyptol, Enzymatic polycondensation, Green solvents, Biomass-derived monomers, Sustainable polymers

## Abstract

Given the urge to accelerate the substitution of petrol-derived solvents not only in more traditional fields like pharmaceuticals, personal care, or electronics but also in innovative research processes, this work focuses on the utilisation of four biobased solvents as media for the enzymatic synthesis of aliphatic-aromatic polyesters. As building blocks, the lignin-derived diethyl-2,4-pyridinedicarboxylate was selected as the potentially biobased, aromatic component while more classical diols such as 1,4-butanediol and 1,8-octanediol were used as the aliphatic portion. Results show that among the tested green solvents (cyclohexanone, phenetole, anisole and eucalyptol), the most suitable medium for lipase B from *Candida antarctica*-catalysed polycondensation reactions was eucalyptol that allowed reach monomer conversions >95 % and number average molecular weights up to 3500 g⋅mol^−1^. On the other hand, cyclohexanone led to the lowest monomer conversions (<80 %) and molecular weights (M_n_<500 g⋅mol^−1^) confirming once again the unsuitability of ketone-containing solvents for enzymatic esterification and transesterification reactions. The lipase could be used up to three times, in eucalyptol as a solvent, without a significant decrease in monomer conversion or molecular weight.

## 1 Introduction

Due to the concerns about environmental preservation and the search for more sustainable materials, processes for polymer production based on Green Chemistry principles have been gaining importance. Different issues need to be considered, mainly regarding the origin of the monomers, the nature of the catalysts, the solvents used and the biodegradability of the polymers.

Currently, primary plastics production processes are fossil-fuel-based, accounting for 93 % of plastics production in 2019 (OECD, 2023). Secondary plastics (recycled) account for around 6 % of plastics production while biobased plastics just for 0.6 % (OECD, 2023). Due to the urgent need to replace polymers of fossil origin, many bio-based monomers from renewable feedstocks have been suggested for polymer synthesis (Pellis et al., 2016). Many works have studied the synthesis of polymers similar to poly(ethylene terephthalate) (PET), as furan-based polymers, from renewable sources (Feng et al., 2023; Jiang et al., 2015; Wang et al., 2024). Our group previously studied pyridinedicarboxylic acids (PDCAs) for the synthesis of polymers, due to their similarity to the terephthalic unit (Pellis et al., 2019b).

In addition to renewable monomers, another very important point to be considered to make polymerisation processes greener is the replacement of toxic chemical catalysts with biological ones keeping in mind the specific sectors in which biocatalysis proves to be advantageous vs traditional chemical catalysis. Enzymes are non-toxic, potentially reusable and eco-friendly catalysts that have been used in synthetic processes for decades. Furthermore, enzymatic polymerisation has emerged as an important technique for the production of biode-gradable polymers (Hevilla et al., 2021). Enzymes operate in mild reaction conditions, with high control of enantio, chemo- and regioselectivity, and lead to the generation of few by-products. Besides, enzymatic polymerisation avoids problems related to metal catalysts commonly used for polymer synthesis, which cause effects on the environment and toxicity in biomedical materials (Liu et al., 2020).

Finally, to develop more sustainable processes to produce polymers, it is necessary to consider the type of solvent to be used. The enzymatic synthesis of polyesters can be carried out in organic solvent systems or in solvent-free systems. In solvent-free systems, the enzyme is directly mixed within the reagents, leading to efficient polymerisation but in general with low molecular weights, due to mass transfer problems. In organic solvent systems, the substrates are dissolved in a solvent, which makes the substrates more accessible to the enzyme, usually employed in immobilized form, therefore improving the efficiency of the reactions. The physical properties of the solvent, including polarity (e.g. dielectric constant, logP_OW_), water solubility, and the solubility of the reactants in the solvents must be considered since they affect the polymerisation reaction. Furthermore, the solvent must have an appropriate boiling point to conduct the reaction and must be chemically inert (Hevilla et al., 2021; Wang et al., 2022).

Some traditionally used solvents are considered hazardous and environmental pollutants, such as toluene and hexane. Thus, it is essential to replace these solvents (Pellis et al., 2019a). To be considered green, the solvent must undergo an assessment of environmental, health and safety (EHS) and energy demand to produce the solvent. Ideally, it should also be bio-based (Byrne et al., 2016).

In order to overcome the problem of traditional solvents, many studies on polymer synthesis using enzymes have been developed, employing a variety of reaction media. For instance, Gustini et al. (2016) successfully demonstrated the synthesis of sorbitol-containing polyesters catalyzed by lipase in a solvent-free system, but they reported a limitation of the system related to the solubilization of some reagents. Additionally, various bio-based solvents have been investigated. The enzymatic polycondensation of 2H-HBO-HBO, a bicyclic diol synthe-sized from levoglucosenone, was demonstrated using Cyrene as a solvent (Warne et al., 2023).

Other non-traditional media such as supercritical carbon dioxide, ionic liquids, and deep eutectic solvents have also been studied for polymer synthesis. Sagnelli et al. (2021) demonstrated the polymerization of a renewable monomer, CHA, extracted from birch bark. They used CaLB as a catalyst and supercritical carbon dioxide, achieving a Mn of 7000 g/mol at 35 °C, temperature significantly lower than those typically employed in conventional reactions. Additionally, the synthesis of bio-based furanic-aliphatic polyesters via enzymatic polymerization in ionic liquids and deep eutectic solvents, was also demonstrated, with a yield of up to 56 % (Silvianti et al., 2022).

**Fig. 1:**
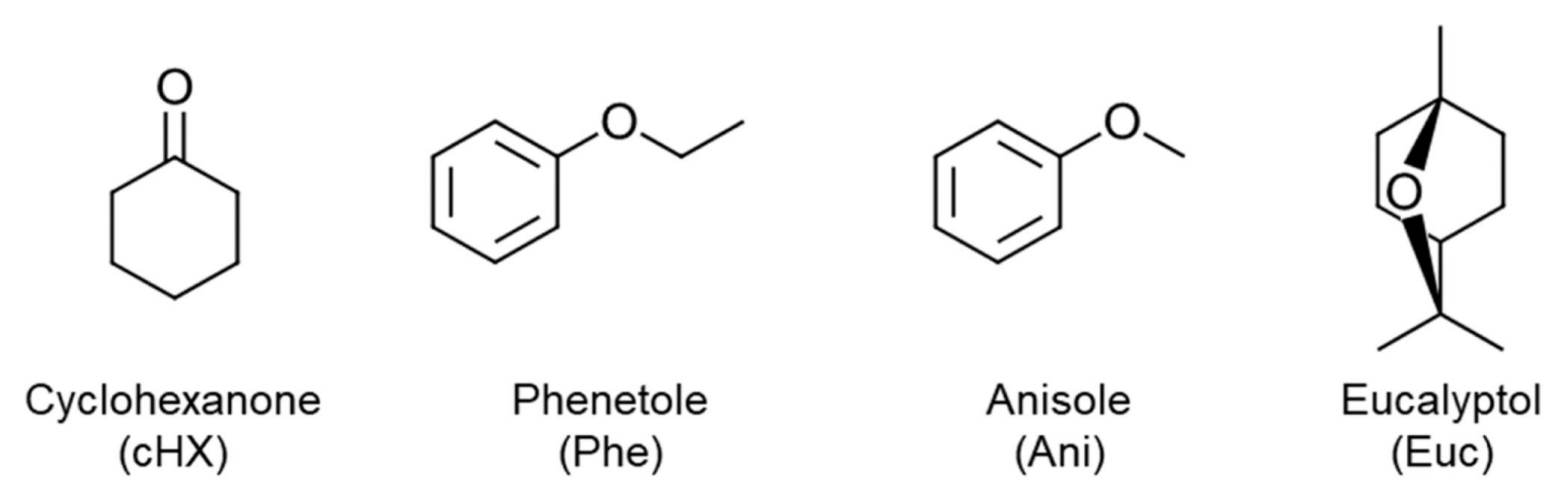
The four potentially biobased solvents used in this work for the enzymatic synthesis of aromatic polyesters.

Our group successfully conducted enzymatic polymerisations of lignin-derivable pyridine diesters using immobilised *Candida antarctica* lipase B (CaLB) as biocatalyst (reactions conducted without solvent and in diphenyl ether, DPE, as the organic medium). The best results in terms of highest molecular weights were obtained in DPE (Pellis et al., 2019b). The use of DPE has proven to be a valuable option for producing polymers with high molecular weights. However, given the difficulty in removing this solvent from the reaction product, in this study, we implemented a new approach based on lower boiling green solvents that have the possibility of being easily removed under reduced pressure to purify the reaction product. This choice had the double aim of avoiding the time-consuming precipitation process for product recovery that was previously used with DPE while obtaining polymers with high molecular weights, a feature not achievable using solvent-free reaction systems. The solvents selected for this work were: cyclohexanone (cHX), phenetole (Phe), anisole (Ani) and eucalyptol (Euc) (Fig. 1).

## 2 Materials and methods

### 2.1 Chemicals and enzyme

Diethyl pyridine-2,4-dicarboxylate (PD24) was purchased from Carbosynth. Diethyl pyridine-2,5-dicarboxylate (PD25) was purchased from TCI. 1,8-octanediol (ODO) and 1,4-butanediol (BDO) were purchased from Acros Organics. Tetrahydrofuran and chloroform were purchased from Fisher Scientific. All other chemicals and solvents were purchased from Sigma-Aldrich. *Candida antarctica lipase* B (CaLB) immobilized onto acrylic resin (iCaLB) was purchased from Sigma-Aldrich (product code L4777, also called Novozym 435) with a determined PLU activity of 5600 U g^−1^.

### 2.2 Synthetic activity of lipases

The synthesis of propyl laurate was carried out at 55 °C using an orbital shaker (250 rpm) in a 20 mL vial using equimolar amounts of lauric acid and 1-propanol (1.2 g and 0.36 g respectively). An amount equal to 10−20 mg of the immobilized enzyme was added to the substrates and the formation of the ester was monitored by ^1^H NMR in the first 15 % of conversion. One enzymatic unit is expressed as the amount of enzyme able to catalyze the formation of 1 μmol of propyl laurate per min at 55 °C.

### 2.3 Enzymatic polymerizations in organic media

*Candida antarctica* lipase B (CaLB) immobilized onto acrylic resin was vacuum dried for 24 h and stored in a desiccator before use. For the reactions, 8⋅10^−4^ mol of diester (diethyl pyridine-2,4-dicarboxylate) (0.2 mol/L) and 8⋅10^−4^ mol of 1,8-octanediol (0.2 mol/L) (diester:diol = ratio 1:1) were added in 4 mL of solvent in a 25-mL round bottom flask. The solvents tested were: anisole, eucalyptol, cyclohexanone and phenetole. The mixture was then stirred at 85 °C until complete dissolution of the monomers in the solvent. In total, 10 % w⋅w^−1^ (calculated on the total amount of the monomers) of lipase was then added and the reaction was run at 85 °C and 270 rpm. In some cases (reactions conducted for 48 h and 72 h), a vacuum of 360 mbar was applied after 6 h of reaction. The biocatalyst was filtered off and then the solvent was removed *in vacuo* using a rotary evaporator. The experiments were performed in duplicate Table 1.

In the enzyme reuse experiment, the polymer synthesis reaction was performed four times using the same enzyme. After each reaction, the enzyme was removed from the flask by dissolving the product with 2-methyl THF. The enzyme was then filtered out, washed with 2-methyl THF, and dried in a desiccator in the refrigerator overnight. The enzyme was then reused for the next reaction.

Control reactions were carried out without the addition of the bio-catalyst to the reaction system and did not lead to any appreciable monomer conversion.

### 2.4 Nuclear magnetic resonance (NMR) spectroscopy

^1^H NMR spectroscopic analysis was performed on a JEOL JNM-ECS spectrometer at a frequency of 400 MHz. CDCl_3_ was used as the NMR solvent.

#### 2.4.1 Gel permeation chromatography (GPC)

GPC was carried out using a set (PSS SDV High) of 3 analytical columns (300 × 8 mm, particle diameter 5 μm) of 1000, 1000000 and 10000000 Å pore sizes, plus a guard column, supplied by Polymer Standards Service GmbH (PSS) installed in a PSS SECurity SEC system. Elution was with THF at 1 mL/min with a column temperature of 30°C and detection by refractive index. 20 μL of a 3 mg/mL sample in THF, with a small quantity of toluene added as a flow marker, was injected for each measurement and eluted for 45 min. Calibration was carried out in the molecular weight range 400–20000000 g⋅mol^−1^ using ReadyCal polystyrene standards supplied by Sigma Aldrich, and referenced to the toluene peak.

### 2.5 Differential scanning calorimetry (DSC)

DSC experiments were performed on a TA Instruments Q2000 DSC under an inert gas atmosphere (N_2_). The used heating and cooling rates were set to 5 °C/min over the T range of −60 to 200 °C. Sample mass between 5 and 10 mg was used for all samples. The Tg values were reported from the second heating scan.

### 2.6 Thermogravimetric analysis (TGA)

TGA was performed on a PL Thermal Sciences STA 625 thermal analyser. Approximately 10 mg of accurately weighed sample in an aluminium sample cup was placed into the furnace with an N_2_ flow of 55 mL⋅min^−1^ and heated from room temperature to 625 °C at a heating rate of 10 °C⋅min^−1^. From the TGA profiles, the temperatures at 5, 10 and 50 % mass loss (TD5, TD10 and TD50, respectively) were subsequently determined.

### 2.7 ATR FT-IR Spectroscopy

ATR FT-IR spectra were recorded on a Perkin Elmer Spectrum 400 spectrometer. The ATR accessory (supplied by Specac Ltd., UK) was equipped with a diamond crystal. A total of 32 scans for each Polymer sample were taken with a resolution of 2 cm^−1^.

### 2.8 Computational studies

Molecular conformations of molecules were calculated with COS-MOconfX (version 4.0; COSMOlogic GmbH & Co. KG, Leverkusen, Germany, 2015) while COSMOthermX (version C30_1705; COSMOlogic GmbH & Co. KG, 2017, TZVP basis set level) was used to provide molecular surface charges and σ-potentials.

## 3 Results and discussion

### 3.1 Screening of green solvents for the enzymatic polymerisation of bio-based Screening of green solvents for the enzymatic polymerisation of bio-based aromatic monomers

The solvents (Fig. 1) were selected based on their structural similarity to toluene and known replacements for toluene (Pellis et al., 2019a). They are also potentially bio-derived and present less severe health and safety hazards compared to toluene (Prat et al., 2016). All these solvents are readily available on the marked and can be purchased also in large quantities despite their actual price exceeds the one of toluene. Anisole and phenetole can be easily prepared starting from phenol obtained from lignin using Williamson’s synthesis (Scheme 2) (Yoshikawa et al., 2013). Anisole can then be subjected to a hydrogenation reaction using a KBr-modified Pd/C catalyst and conducting the reaction in an H_2_O/CH_2_Cl_2_ mixture that yields up to 96 % cyclohexanone (Meng et al., 2017). Eucalyptol is prepared by fractional distillation at a temperature of 170–180 ºC starting from eucalyptol-rich essential oils (Burdock, 2009).

These solvents were also selected as they are rather hydrophobic and from previous works it is known that lipases, such as the *Candida antarctica* lipase B (CaLB) selected in this work, maintain a better synthetic activity when employed in less polar media (Kumar and Gross, 2000; Pellis et al., 2019a; Yao et al., 2011). In Table 2 we compared the Hansen solubility parameters of the selected bio-based solvents with the data of toluene, the petrol-based solvent that is a typical solvent for enzymatic ring-opening polymerisations of lactides (Düskünkorur et al., 2015; Hans et al., 2009). The Hansen solubility parameters correlate to solubility, with the hydrocarbon toluene having the lowest dipolarity (δ_P_) and hydrogen bonding (δ_H_) parameters (Abbott and Yamamoto, 2015). The aromatic solvents anisole and phenetole are more polar than toluene due to their additional ether functional group. Eucalyptol also has a low polarity, while cyclohexanone is more polar due to the ketone functional group. The Kamlet-Abboud-Taft parameters also describe polarity but correlate to chemical phenomena such as kinetics and equilibria (Jessop et al., 2012; Marcus, 1993). All solvents are aprotic (α = 0). Anisole and phenetole exhibit low hydrogen accepting ability (β), whereas the aliphatic ether eucalyptol is a strong hydrogen bond acceptor. Eucalyptol has a low dipolarity (π*) while the other solvents have greater dipolarity or polarisability than toluene. The donor number (DN) is somewhat proportional to β (Marcus, 1993). Partition coefficients are sometimes used to represent polarity. Anisole and phenetole have a similar 1-octanol-water partition coefficient (logP_OW_) to toluene, while cyclohexanone is more hydrophilic and eucalyptol less hydrophilic than the other solvents in this set. The health and safety scores of cyclohexanone, anisole, phenetole, and eucalyptol are all preferable to toluene (Prat et al., 2016). The environment scores of the solvents are moderate, which is a consequence of their boiling points and the energy requirement of recovery by distillation.

For polycondensation reactions, monomer conversion rates were calculated via ^1^H NMR spectroscopy (^1^NMR spectra can be found in Supplementary Figures 2−18). Product formation was also confirmed by FT-IR (Supplementary Fig. 44).

**Fig. 2:**
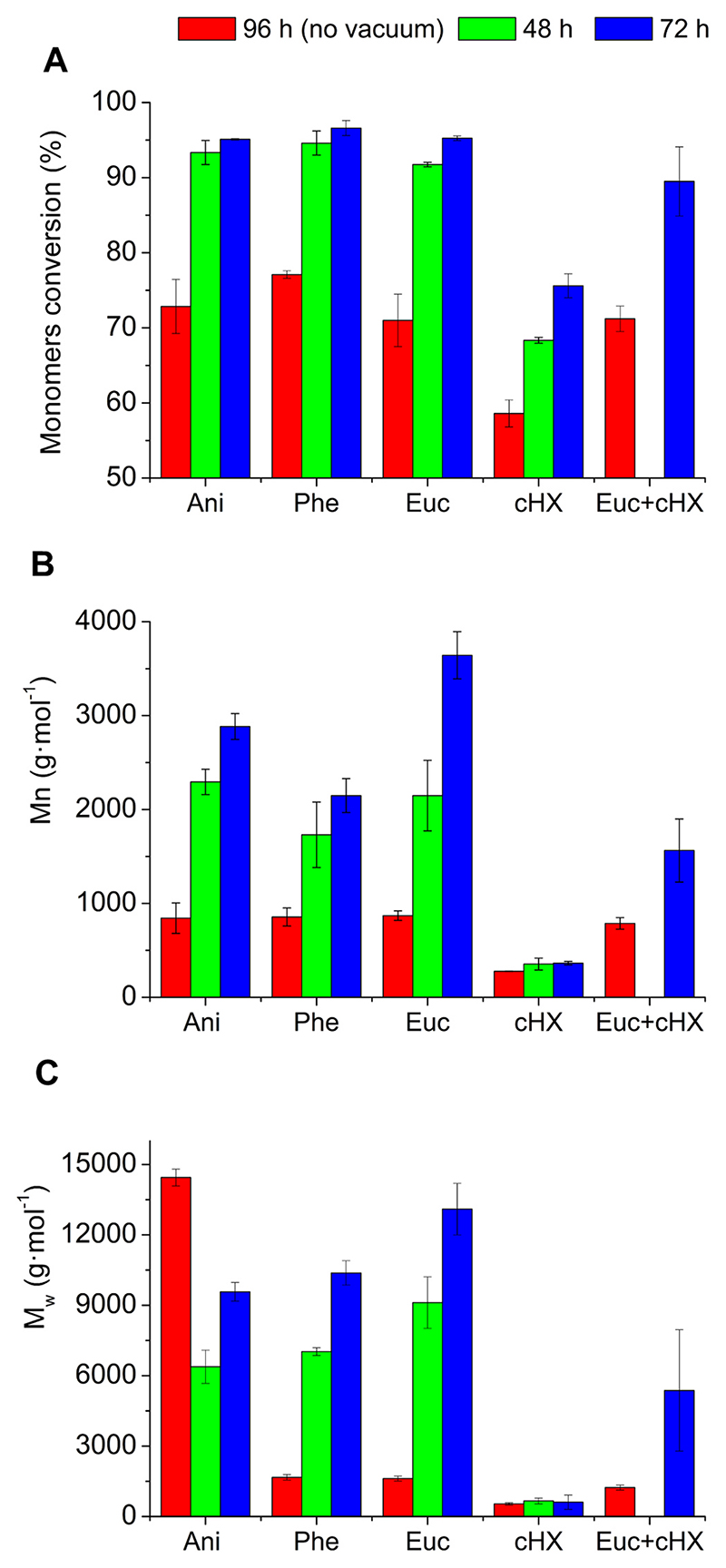
Enzymatic synthesis of poly(1,8-octylene-2,4-pyridinedicarboxylate) in various conditions. A) Monomers conversion calculated via ^1^H NMR spectros-copy; B) number average molecular weight (M_n_) calculated via GPC; C) weight average molecular weight (M_w_) calculated via GPC. Euc+cHX: 9:1 Eucalyptol + cyclohexanone solvents mixture.

When performing the polycondensation reaction of PD24 and ODO, the best monomer conversion rates were obtained after 72 h of reaction keeping the system under a 360 mbar vacuum for 66 h (Fig. 2A, blue bars). Using this condition **3**, conversion rates for anisole, eucalyptol and phenetole were all >95 % while for cyclohexanone a 76 % monomer conversion was observed. Slightly lower conversions were obtained using condition **2** (Fig. 2A, green bars) most likely due to the reduced reaction time (48 h for **2** vs 72 h for **3**). The worst conversion results were obtained using condition **1** (96 h of total reaction time, no vacuum applied, Fig. 2A, red bars).

**Scheme 1:**
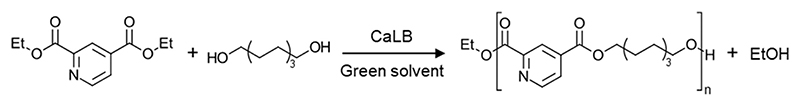
Polycondensation reaction between diethyl pyridine-2,4-dicarboxylate (PD24) and 1,8-octanediol (ODO) catalysed by the lipase B from *Candida antarctica*, (CaLB) yielding the pyridine-based polyester poly(1,8-octylene-2,4-pyridinedicarboxylate) (pPD4-ODO).

The polycondensation reaction between diethyl pyridine-2,4-dicarboxylate (PD24) and 1,8-octanediol (ODO) (Scheme 1) was selected as a model reaction as it yielded the best results in terms of conversion (~98 %) and obtained molecular weights (M_n_ ~14000 g⋅mol^−1^) when it was carried out in diphenyl ether (DPE) as the reaction solvent (Pellis et al., 2019b).

**Scheme 2:**
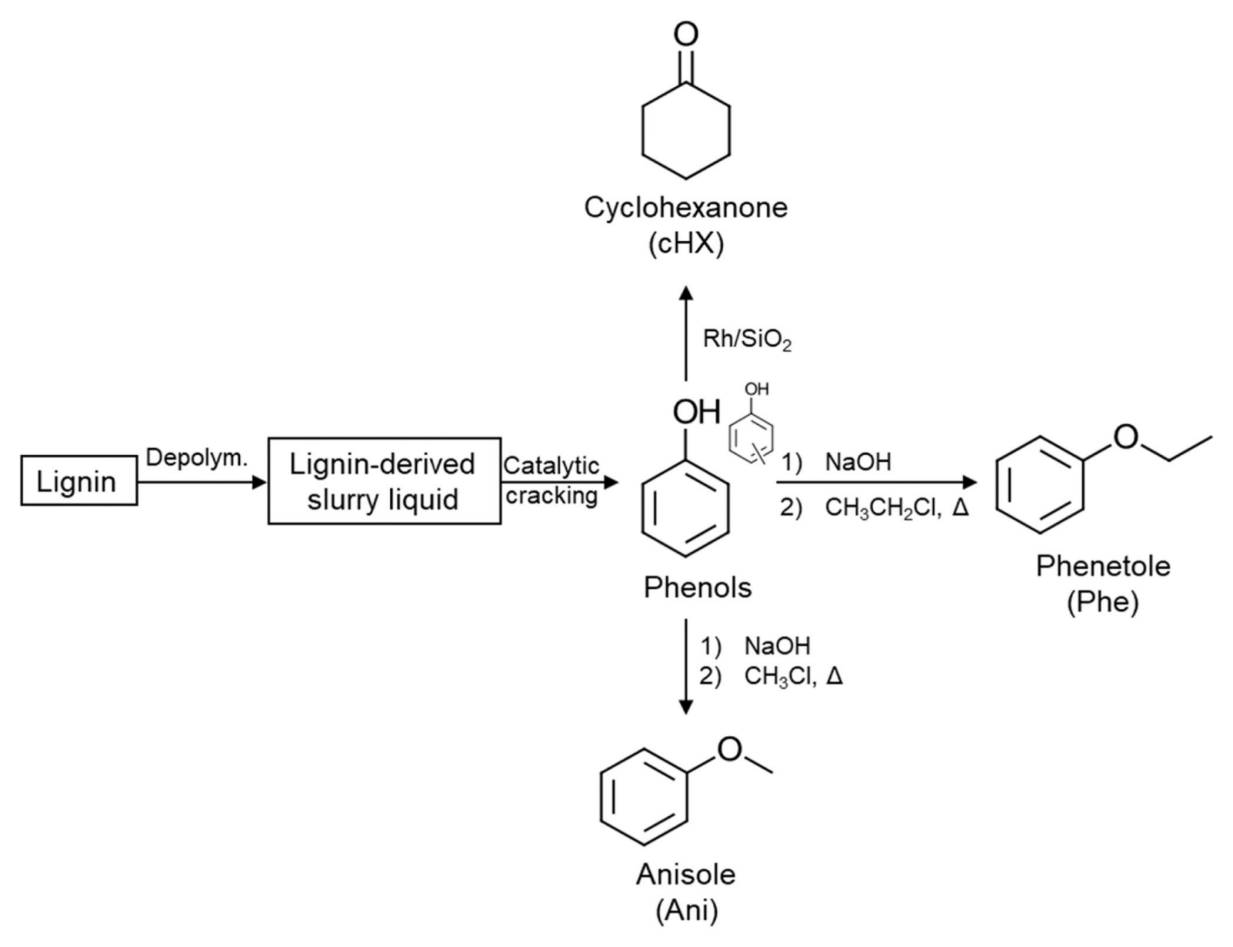
Production routes of phenetole, anisole and cyclohexanone starting from lignin.

This reaction was carried out using three different conditions detailed in Table 1 to find the best reaction conditions in the newly selected low-to-moderate boiling solvents anisole, eucalyptol, cyclo-hexanone, and phenetole. To be noted that grinding of the biocatalyst due to magnetic stirring was not observed and it was possible to recover >95 % of the beads that were than used for the recycling experiments Scheme 2.

The applied vacuum had a crucial role in the progression of the step-growth polymerisation as it enabled the constant removal of the ethanol produced in the reaction, shifting the reaction equilibrium towards the polymer formation. Note that the reaction in the ketone solvent, cyclo-hexanone, was significantly less effective than the ether solvents.

In terms of molecular weight, the longest chains were obtained with reactions in eucalyptol, which using condition **3** led to polyesters having a M_n_ of 3600 g⋅mol^−1^ and a M_w_ of 13100 g⋅mol^−1^ (Figs. 2B and C). Also, in the case of the obtained molecular weights the obtained trend shows how condition **3** outperforms conditions **1** and **2** and that eucalyptol is the preferred solvent to carry out this reaction.

**Fig. 3:**
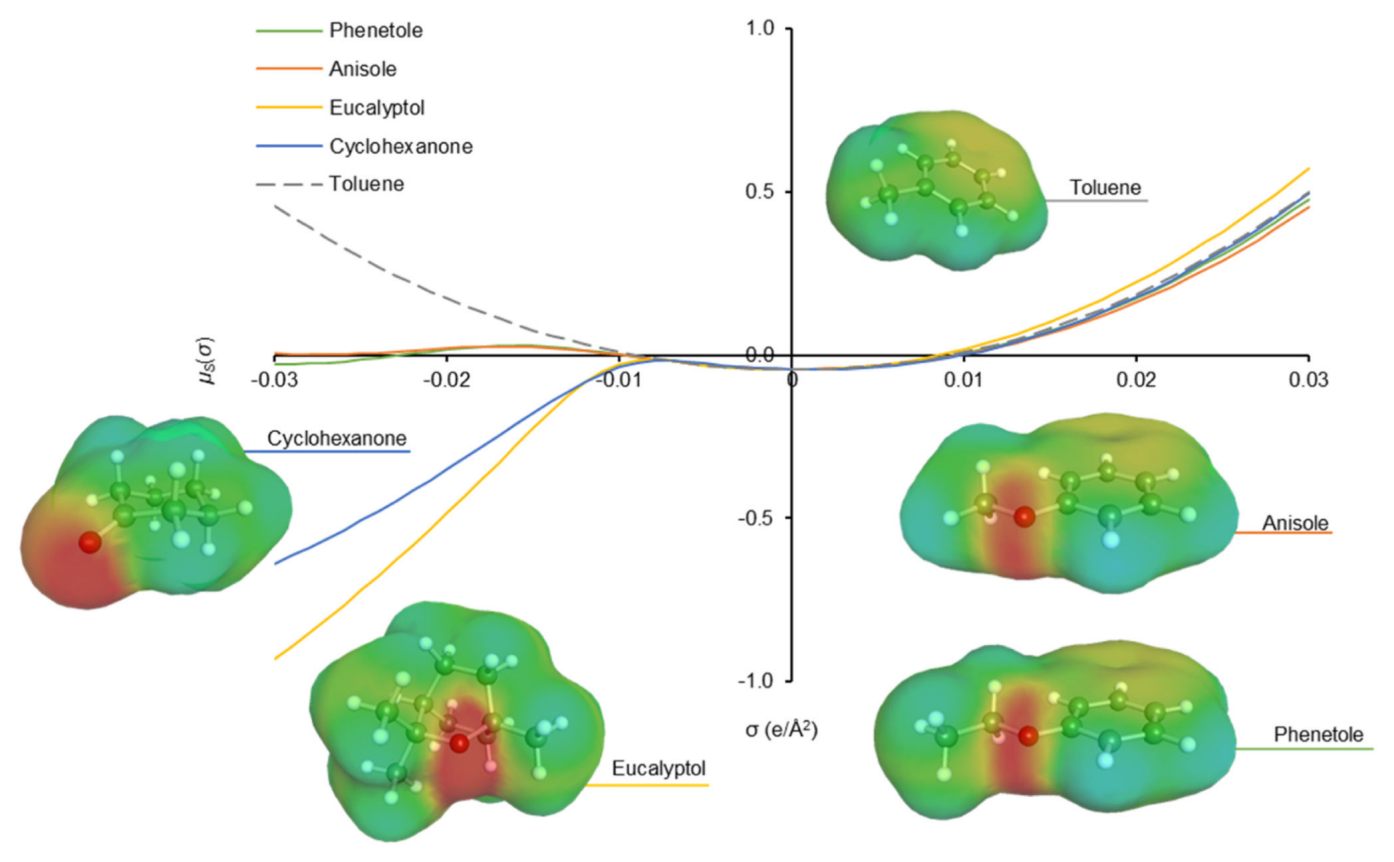
Chemical potentials of a molecular surface of charge σ in a variety of solvents (σ-potential). Annotated with the molecular structures of solvents including electron density maps (σ-surface).

To try to understand the observed trend, a deeper investigation of the solvent effect was carried out. As stated before, toluene is the conventional solvent for these transformations (Flores et al., 2019) and among anisole, phenetole and eucalyptol, the latter is the least similar to the structure of toluene, but its use yielded the greatest molecular weight polymers. Whereas toluene has the lowest magnitude hydrogen bond accepting parameter (β), donor number (DN), and dipolarity (δ_D_) on the Hansen solubility parameter scale between these solvents (Table 2), eucalyptol has the largest values (Abbott and Yamamoto, 2015; Jessop et al., 2012; Marcus, 1993). To further understand the polarity of the solvent set, the σ-potentials of the solvents were produced using COS-MOtherm (Fig. 3) (Klamt et al., 2010). Eucalyptol is described as a strong electron donor, with the aromatic ethers anisole and phenetole only weakly interacting with electropositive species (Fig. 3). Toluene repels charges due to its apolar nature. The dissimilarity between toluene and eucalyptol means the properties of the resultant polymers are not determined solely by the polarity of the solvent in which they were synthesised. When performing the synthesis of poly(1, 8-octylene-2,4-pyridinedicarboxylate) in toluene as control reaction, lower monomer conversions when compared to anisole, phenetole and eucalyptol were observed (71 % for the reaction at 48 h, 81 % for the reaction at 72 h and 56 % for the reaction at 96 h) demonstrating experimentally the superiority of the biobased solvents vs the traditionally used petrol-based solvents for enzymatic polycondensation reactions.

The single solvent parameter that appears to correspond to the performance of the enzymatic polymerisations is logP_OW_. Historically, there has been an association between the octanol-water partition coefficient and the efficacy of enzymatic catalysis in organic media (Laane et al., 1987). The logP_OW_ parameter can be described more precisely as a function of hydrogen bond accepting parameter (β) and dipolarity (π*) and molar volume (Kamlet et al., 1984), and the rate of bio-catalysed esterifications has previously been described as a function of β and molar volume in preference to logP_OW_ (Paggiola et al., 2014). A low β value is preferable to minimise interaction with the condensation products of the esterification or transesterification, which could affect equilibria and enzyme stability, and a large molar volume reduces cohesive energy density (i.e. intermolecular interaction energy within a specific volume). In this work, even though eucalyptol has a high β value, its large molar volume compensates for this effect (as evidenced by its high logP_OW_ value) and may explain its high performance as a toluene substitute.

Because reactions in eucalyptol resulted in polymers with the highest molecular weights, and this solvent possesses the advantage of being both a green and renewable solvent, it was selected for further studies.

### 3.2 Reactions doping with cyclohexanone

To better understand the interaction between cyclohexanone and the enzyme, reactions in eucalyptol were doped with 10 % v/v cyclohexanone. While the reduction in monomer conversion was small (95.3 % for eucalyptol *vs*. 89.5 % for the doped reaction after 72 h, Fig. 2A), a more significant effect was observed on the obtained molecular weights. The reactions in the eucalyptol-cyclohexanone blends yielded polymer chains less than half the M_n_ (1600 *vs*. 3600 g⋅mol^−1^) and almost three times lower M_w_ (5400 *vs*. 13100 g⋅mol^−1^) than the equivalent materials obtained using eucalyptol alone (results are shown in Fig. 2). This observation concurs with prior molecular modelling studies that report that solvents containing ketone moieties (e.g. 2-pentanone, 3-pentanone) show a clear affinity for the active site of the enzyme (Graber et al., 2007). During the performed simulations, Graber et al. demonstrated that this solvent stayed in the active site binding the oxyanion hole and to the catalytic serine with hydrogen bonds. At the end of the simulation the paper reports that the ketone lost its hydrogen bonds and started exploring the active site, but without leaving it. This explains very well the inhibitory effect that solvents containing ketone moieties have on the lipase B from *Candida antarctica*.

### 3.3 Reactions using different aromatic monomers

**Fig. 4:**
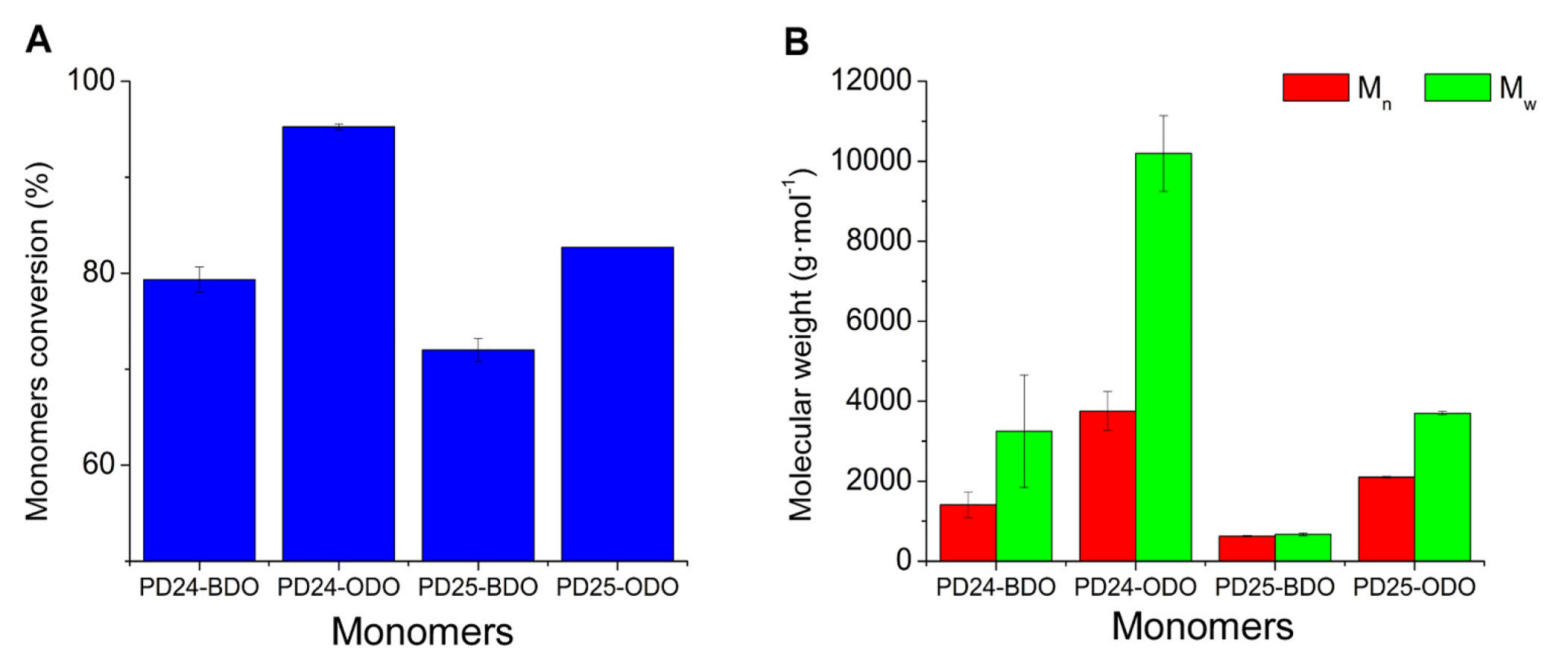
Enzymatic synthesis of pyridine-based polyesters using eucalyptol as the organic medium after 72 h (6 h at 1000 mbar + 66 h at 360 mbar) of reaction. A) Monomers conversion calculated via ^1^H NMR spectroscopy; B) number average molecular weight (M_n_, red bars) and weight average molecular weight (M_w_, green bars) calculated via GPC.

Polymer synthesis was also carried out using another pyridine diester isomer, namely 2,5-diethyl pyridine dicarboxylate (PD25) and a shorter, aliphatic, biobased diol, 1,4-butanediol (BDO). The combinations of the newly selected monomers resulted in conversions between 70 % and 80 % (Fig. 4A) and number average molecular weights between 600 and 2100 g⋅mol^−1^ (Fig. 4B). These results confirm the group’s previous results in the synthesis of these polymers in diphenyl ether that showed how the polymerisation of PD24 is superior to the one of PD25 (similarly to what was observed for the petrol-based diethyl isophthalate and diethyl terephthalate monomers) (Pellis et al., 2019b). Regarding the enzyme’s preference for ODO over BDO several reports describe how lipases show better activity on longer chain, more hydrophobic monomers (Campisano et al., 2021; Debuissy et al., 2017; Jiang et al., 2015; Pellis et al., 2019b).

FT-IR analyses of the products confirmed the presence of bonds typical of polyesters. Supplementary Figure 1 shows the spectrum of ODO only (red line) and PD24-ODO reaction products in eucalyptol (black line). By comparing the product spectrum with the ODO spectrum, one can observe the disappearance of the -OH peaks from ODO and a reduction in the peaks corresponding to the C-H bonds of alkanes. There is the appearance of the peak referring to the C═O bonds and the peaks referring to the C-H bonds of aromatics.

### 3.4 Thermal characterisation of the polyesters

**Fig. 5:**
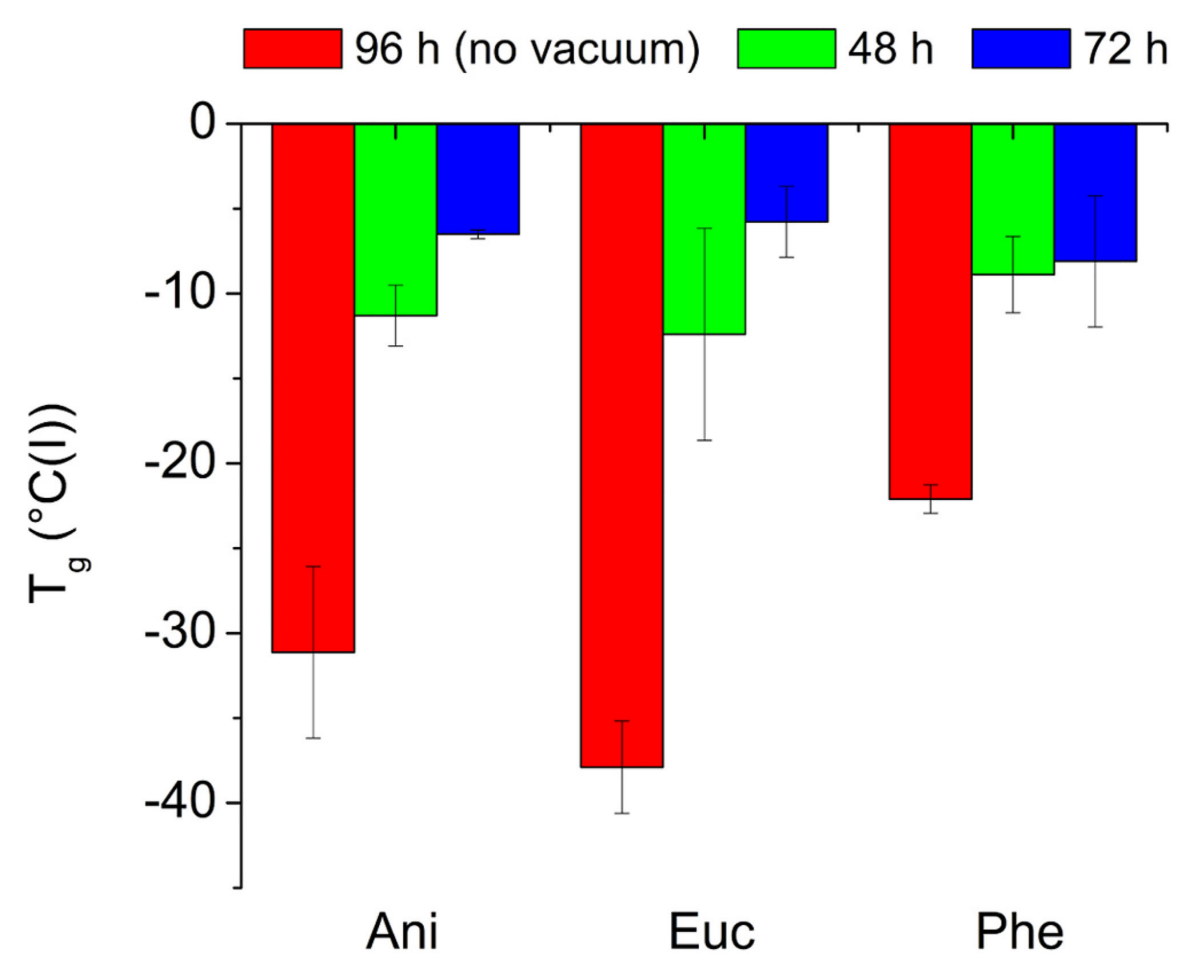
Glass transition temperatures (T_g_) obtained from the DSC analysis of the pPD24-ODO polymers synthesised in various conditions and green solvents.

DSC analyses showed that the reactions using PD24 and ODO led to amorphous reaction products while using PD25 and ODO led to more crystalline polymers (the only products with a melting point). The same observation was previously reported in a publication by our group for the polymerisation of the same monomers in solventless bulk reaction systems and in DPE as a solvent (Pellis et al., 2019b). The Tg values of the pPD24-ODO are shown in Fig. 5, where it can be seen that the thermal properties depend on the molecular weight of the polymer. The Tg values in the different solvents were similar, and as the molecular weight of the polymers increased, the Tg also increased (for the complete set of DSC data see Supplementary Table 2 and Supplementary Figures 33–43).

The pPD25-ODO polymer showed a melting point of 93 °C, similar to what was previously reported for the same reaction conducted in bulk (95 °C) (Pellis et al., 2019b).

The reactions performed in cyclohexanone (PD24-ODO) and the reactions using PD25 and BDO in eucalyptol led to products with very low molecular weight, so these samples were not analysed by DSC.

**Fig. 6:**
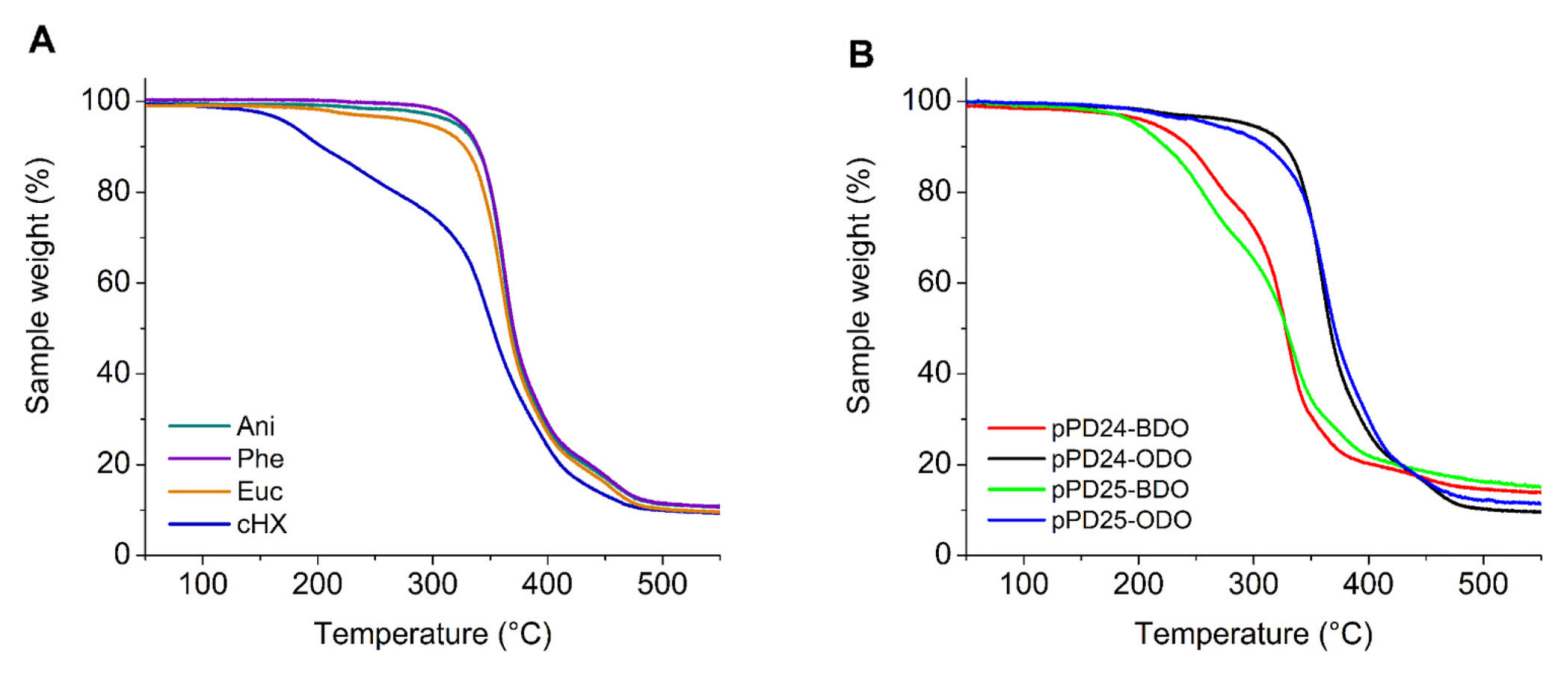
Thermogravimetric analysis of the synthesised polymers. A) pPD24-ODO synthesised in the various green solvents using condition 3. Anisole (green line), eucalyptol (orange line), phenetole (purple line) and cyclohexanone (blue line). B) polymers synthesised in eucalyptol using condition 3 and various diesters (PD24 and PD25) and aliphatic diols (C4, BDO and C8, ODO). pPD24-BDO (C4, red line); pPD24-ODO (C8, black line); pPD25-BDO (green line) and pPD25-ODO (blue line).

The thermal stability of the polyesters produced in different solvents was investigated by TGA. Apart from the reaction in cyclohexanone which led to low molecular weight products, polyesters produced in the other solvents appeared to be thermally stable up to around 300 °C with less than 5 % weight loss (Fig. 6A). The thermal decomposition behaviour was very similar for polymers synthesised in eucalyptol, anisole, and phenetole (for the complete set of TGA data see Supplementary Table 3).

From the thermogravimetric analysis plotted in Fig. 6B, it is possible to see a difference between the polymers synthesised using the C4 and C8 diols both with PD24 and PD25 used as the aromatic diesters. The polymers synthesised using BDO as the diol decompose at a lower temperature than those synthesised using ODO. This trend is consistent with the GPC analysis which shows that ODO-based polymers have higher molecular weights compared to the ones using BDO and therefore they are more thermally stable.

### 3.5 Enzyme reutilization

**Fig. 7:**
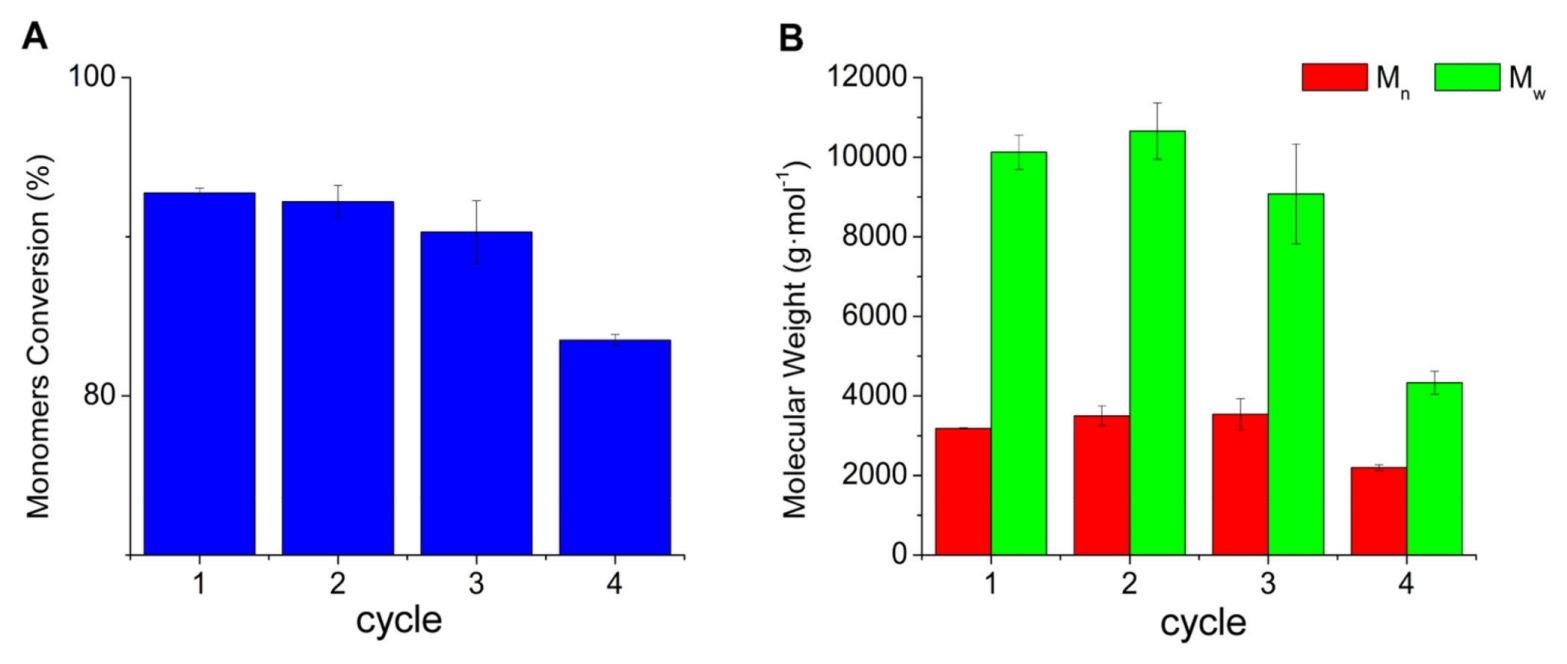
Enzyme reutilization for the synthesis of pPD24-ODO using eucalyptol as a solvent in 72-h cycles (vacuum of 360 mbar applied after 6 h). A) Monomers conversion calculated *via* ^1^H NMR spectroscopy in each cycle. B) number average molecular weight (M_n_) and weight average molecular weight (M_w_) calculated via GPC in each cycle.

After defining the conditions for polymer synthesis, it was tested whether the enzyme could be recycled to reduce process costs. When the biocatalyst was used in successive 72-hour cycles (vacuum of 360 mbar applied after 6 hours), the original conversion and molecular weight were maintained for two more cycles. In the 4th cycle the conversion dropped off 10 % in conversion (Fig. 7A) and 31 % and 57 % in M_n_ and M_w_, respectively (Fig. 7B). This result is better than some reports in the literature using Novozym 435 for polymer synthesis. Skoczinski et al. studied the reuse of the biocatalyst for an oligofuranoate synthesis and observed that the reaction yield decreased by around 20 % in the second cycle and an additional 20 % decrease in the third cycle (Skoczinski et al., 2020). Guckert et al. tested Novozym 435 reutilization in the polycondensation of 1,4-butanediol and diethyl succinate in diphenyl ether as a solvent (Guckert et al., 2023). The yields of the products obtained were highly affected: 69.3 % (1st cycle), 15.0 % (2nd cycle, indicating a decrease of 78.4 % concerning the 1st cycle), and 9.6 % (3rd cycle, indicating a decrease of 86.2 % concerning the 1st cycle).

Enzyme stability depends on many factors, such as temperature, high reaction times, the presence of organic solvent and the presence of substrates. Campisano et al. (2021) investigated the Novozym 435 thermal stability by incubating the enzyme preparation in butanol at 90 C for 96 h, and observed that only 38 % of the immobilised enzyme activity remained after 96 h. Furthermore, the disintegration of the macroporous acrylic resin support material used for the enzyme immobilization (Hevilla et al., 2021; Palsule and Poojari, 2010) and leaching of the enzyme (Hevilla et al., 2021) may also occur.

## 4 Conclusions

In this work four green solvents namely anisole, cyclohexanone, phenetole, and eucalyptol were used as reaction media for the enzymatic synthesis of biobased aromatic-aliphatic polyesters. The best results in terms of monomer conversion (>95 %) and number-average molecular weight (~3500 g⋅mol^−1^) were obtained using eucalyptol as the solvent. On the other hand, cyclohexanone led to the lowest monomer conversions (<80 %) and molecular weights (M_n_<500 g⋅mol^−1^), confirming the unsuitability of ketone-containing solvents for enzymatic esterification and transesterification reactions because of the solvent inhibition effect caused by the interaction of the media with the enzyme’s active site and oxyanion hole. The used enzymatic preparation could be recycled up to three times without significantly losing monomer conversion or the obtained molecular weight. In future works it would be interesting to scale up the optimized reaction to prepare these oligomers on a multi-gram scale since they could be used for further applications as additives or plasticizers.

## Supplementary Material

Supp info

## Figures and Tables

**Table 1:** Details on the reaction conditions that were used to optimise the polymerisation of diethyl pyridine-2,4-dicarboxylate (PD24) and 1,8-octanediol (ODO) using the new green solvents (anisole, eucalyptol, cyclohexanone, or phenetole) and CaLB as the biocatalyst.

Condition	Total time (h)	Time at 1000 mbar (h)	Time at 360 mbar (h)
1	96	96	0
2	48	6	42
3	72	6	66

**Table 2:** Hansen solubility parameters and other properties of the selected solvents and the classically used petrol-based solvent toluene for comparison.

	Toluene	cHX	Phe	Ani	Euc
BP /°C^a^	111	154	193	154	177
δ_D_ /MPa^½b^	18.0	17.8	18.4	17.8	16.7
δ_P_ /MPa^½b^	1.4	8.4	4.5	4.4	4.6
δ_H_ /MPa^½b^	2.0	5.1	4.0	6.9	3.4
α^c^	0.00	0.00	0.00	0.00	0.00
β^c^	0.11	0.53	0.30	0.32	0.61
π*^c^	0.54	0.76	0.69	0.73	0.36
V_M_ /cm^3^·mol^−1b^	107	104	127	109	168
DN^c^	1	18	8	9	24
logP_ow_^a^	2.7	0.86	2.0	2.6	3.4
Safety /10^d^	5	3	4	4	3
Health /10^d^	6	2	1	1	2
Environment /10^d^	3	5	5	5	5

a Data obtained from REACH registration dossiers.

b Data obtained from the HSPiP software (Abbott and Yamamoto, 2015).

c Kamlet-Abboud-Taft solvatochromic parameters (hydrogen bond donating ability, α; hydrogen bond accepting ability, β; dipolarity/polarisability, π*) and donor number (DN) (Jessop et al., 2012; Marcus, 1993).

d CHEM21 solvent selection rankings, with low scores preferred (Prat et al., 2016).

## Data Availability

Data will be made available on request.
