# Supplementary material for: Enzymatic synthesis of aromatic biobased polymers in green, low-boiling solvents
Source: J Biotechnol. Author manuscript; Available in PMC 2024 Nov 6. (PMC7616777; doi:10.1016/j.jbiotec.2024.10.003)
Supplement: Supp info [file EMS199710-supplement-Supp_info.docx]

**Electronic Supplementary Materials**

**Enzymatic synthesis of aromatic biobased polymers in green, low-boiling solvents**

Thaís Fabiana Chan Salum^a,b*^, Daniel Day^a^, James Sherwood^a^, Alessandro Pellis^c,^*, Thomas James Farmer^a^

^a^ University of York, Department of Chemistry, Green Chemistry Centre of Excellence, Heslington, York YO10 5DD, UK

^b^ Embrapa Agroenergy, Parque Estação Biológica, 70770-901, Brasilia, Brazil

^c^ University of Genova, Department of Chemistry and Industrial Chemistry, via Dodecaneso 31, 16146, Genova, Italy

*Corresponding authors: Dr Thaís Fabiana Chan Salum, email: [thais.salum@embrapa.br](mailto:thais.salum@embrapa.br) and Prof. Alessandro Pellis, email: alessandro.pellis@unige.it

**Supplementary figures**


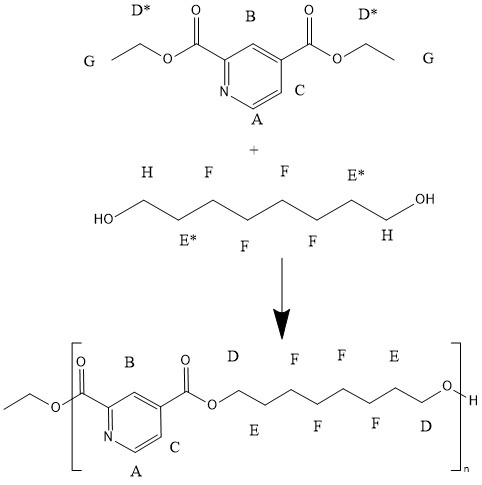


**Supplementary Figure 1.** Polycondensation reaction between diethyl pyridine-2,4-dicarboxylate (PD24) and 1,8-octanediol (ODO) yielding the pyridine-based polyester poly(1,8-octylene-2,4-pyridinedicarboxylate) (pPD4-ODO).


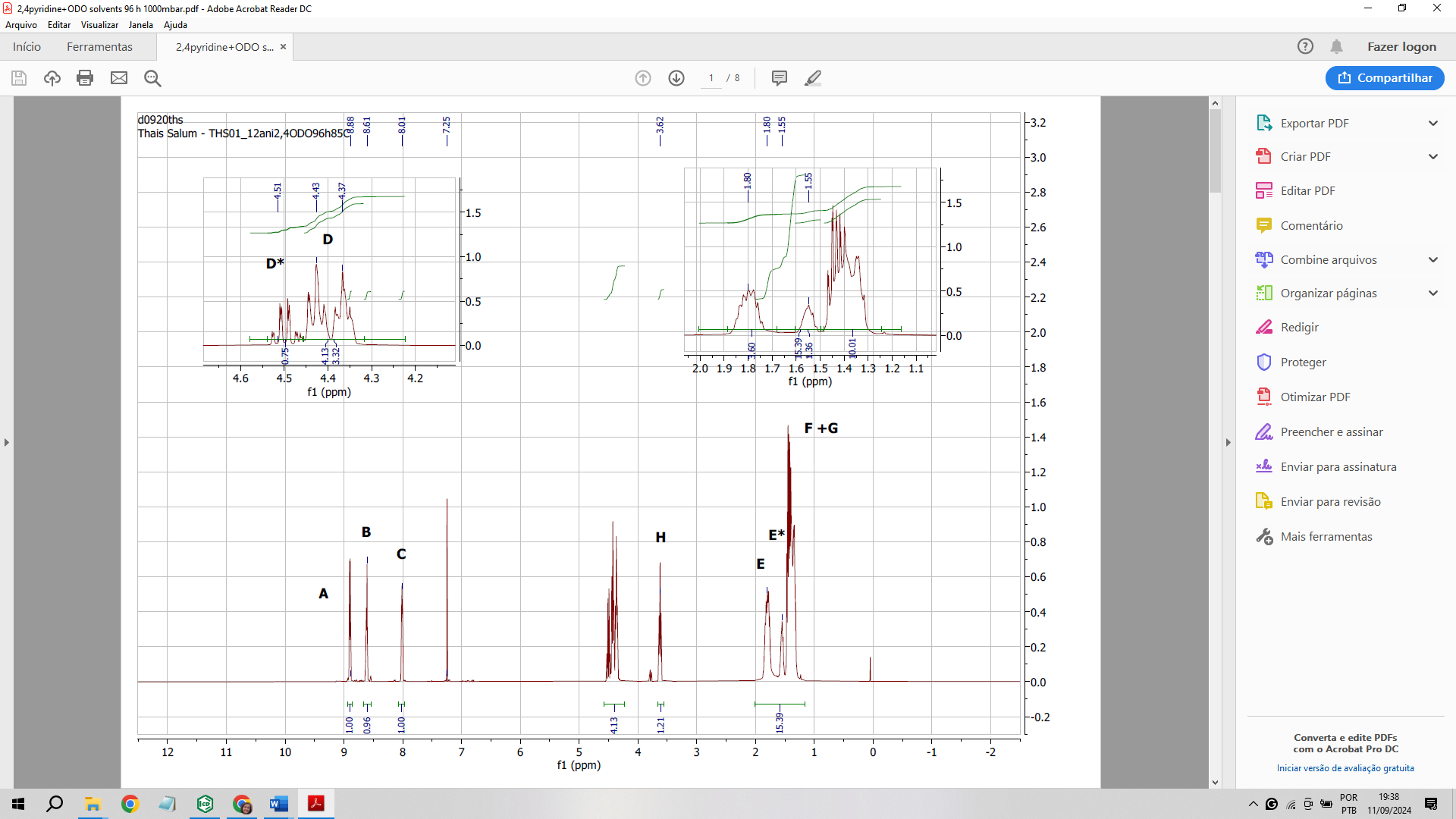


**Supplementary Figure 2.** ^1^H-NMR spectrum of the polymer PD24-ODO synthesized in anisole as a solvent after 96 h (no vacuum) of reaction.


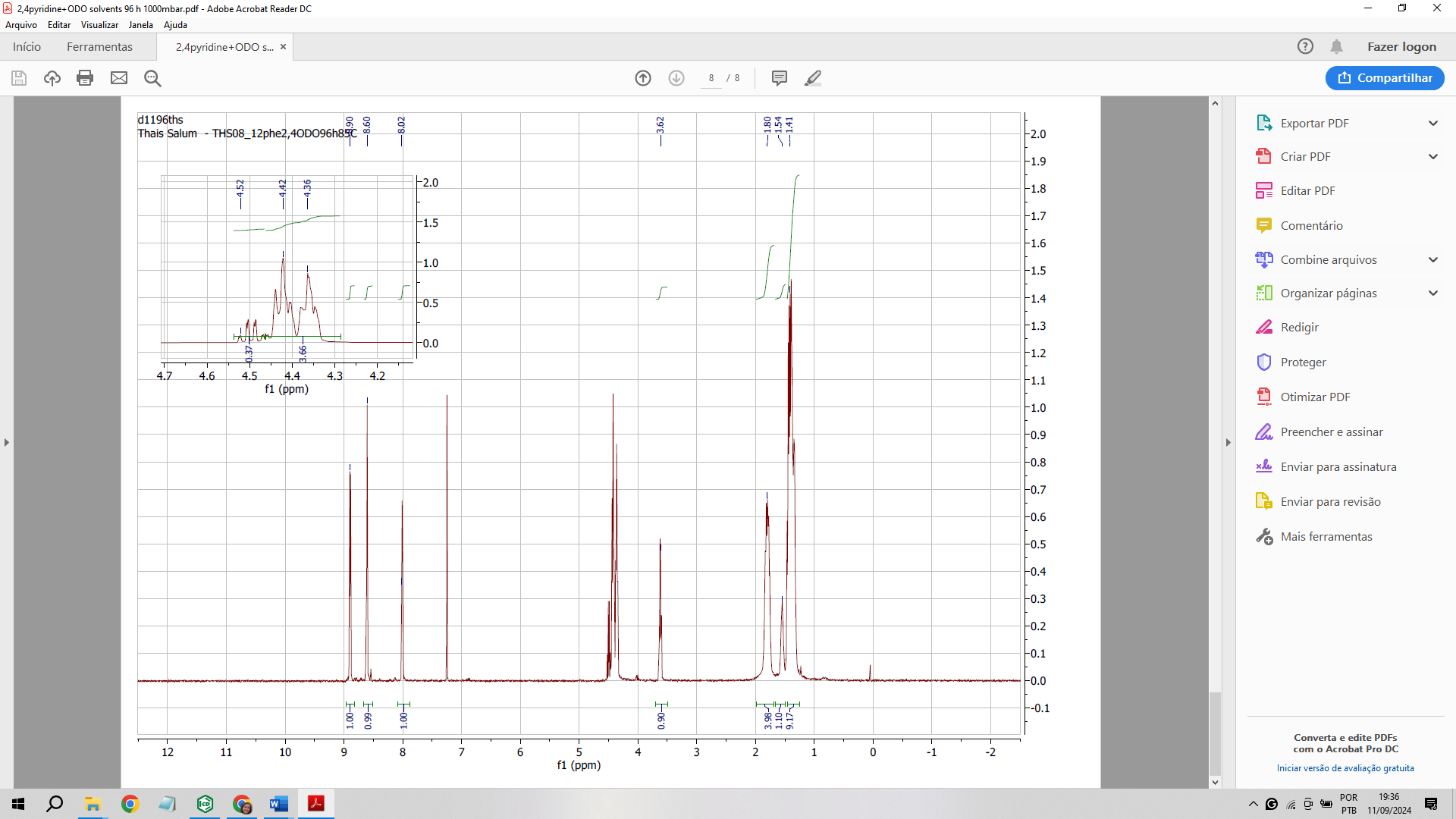


**Supplementary Figure 3.** ^1^H-NMR spectrum of the polymer PD24-ODO synthesized in phenetole as a solvent after 96 h (no vaccum) of reaction.


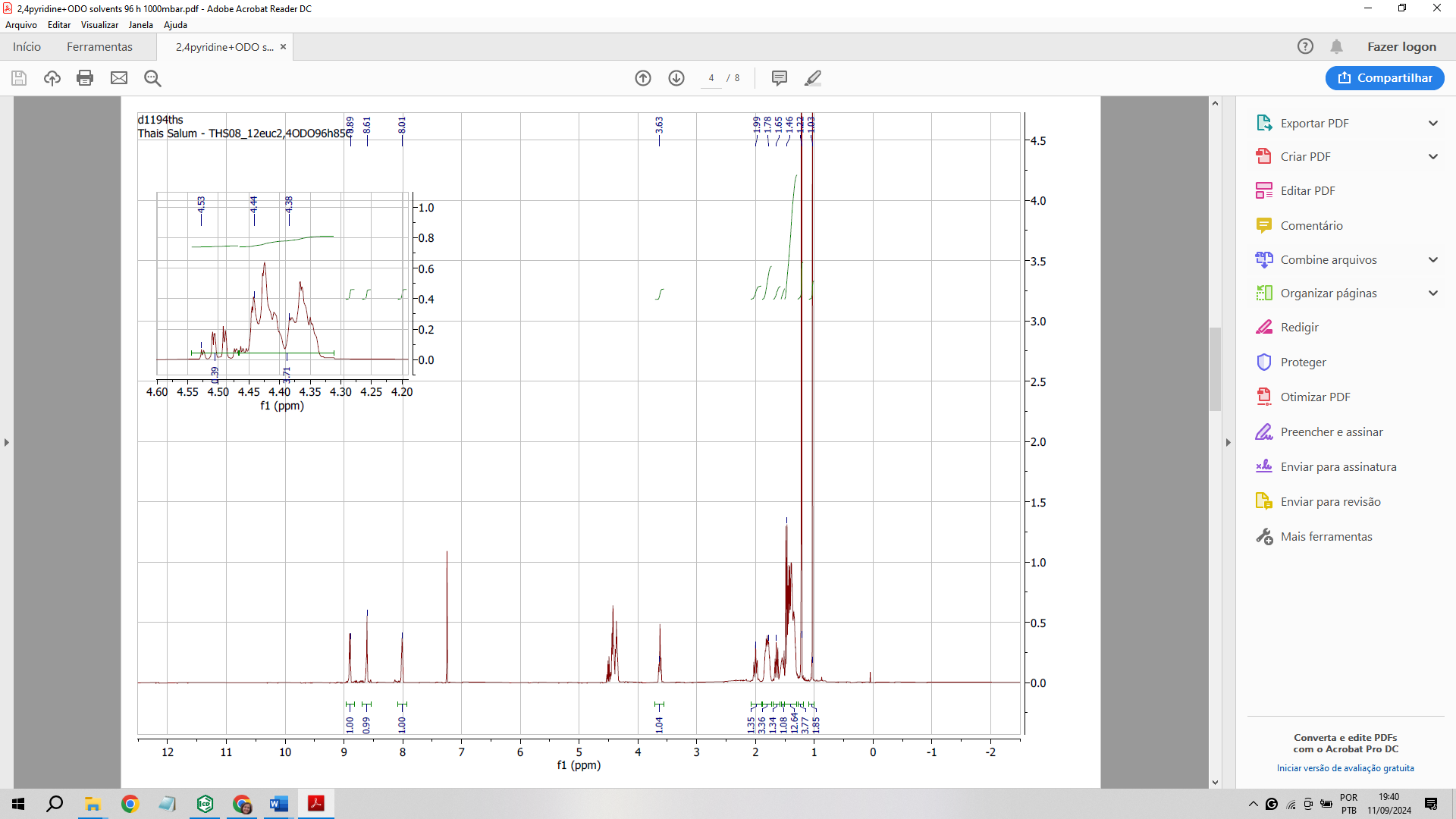


**Supplementary Figure 4.** ^1^H-NMR spectrum of the polymer PD24-ODO synthesized in eucalyptol as a solvent after 96 h (no vacuum) of reaction.


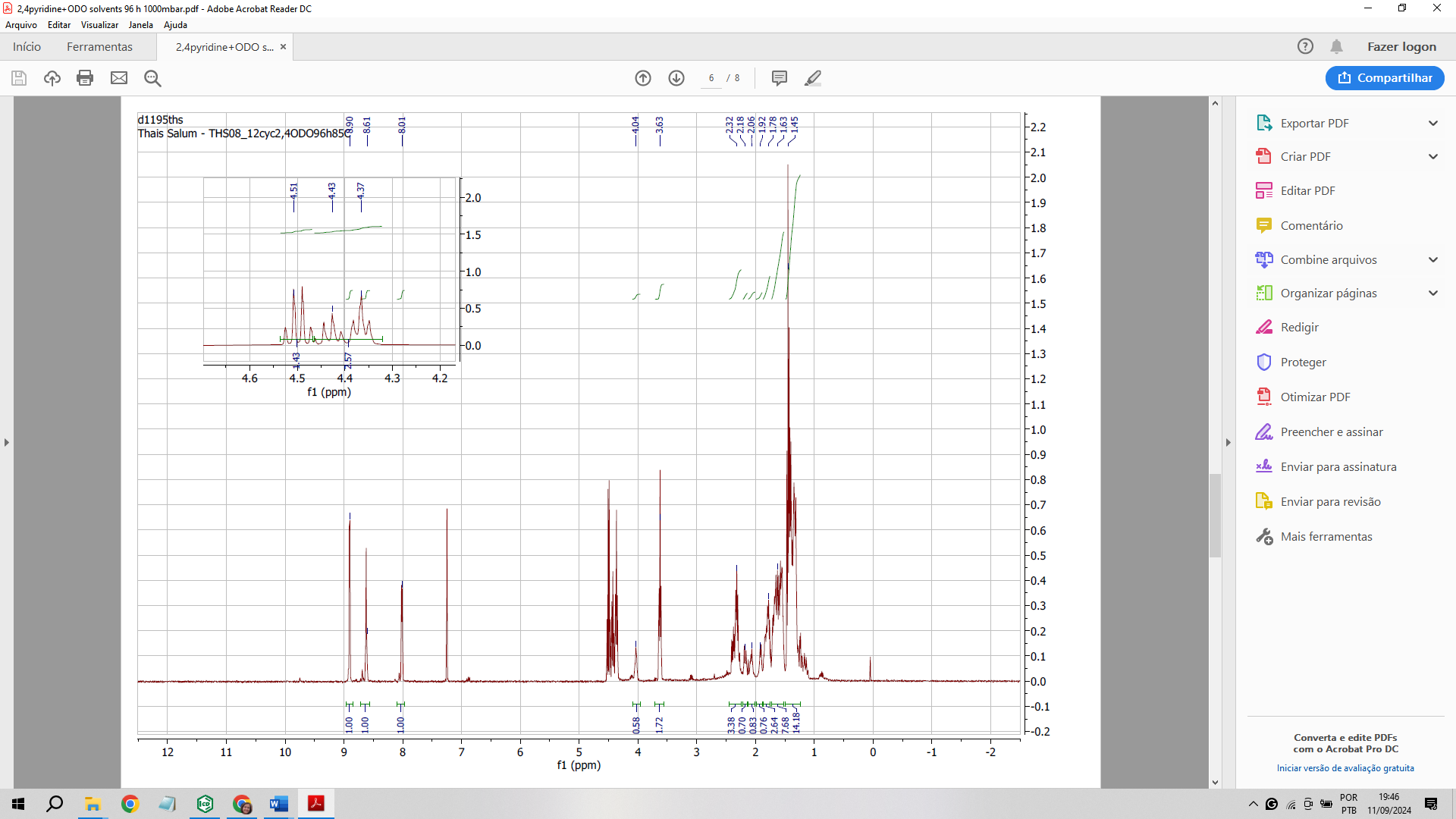


**Supplementary Figure 5.** ^1^H-NMR spectrum of the polymer PD24-ODO synthesized in cyclohexane as a solvent after 96 h (no vacuum) of reaction.


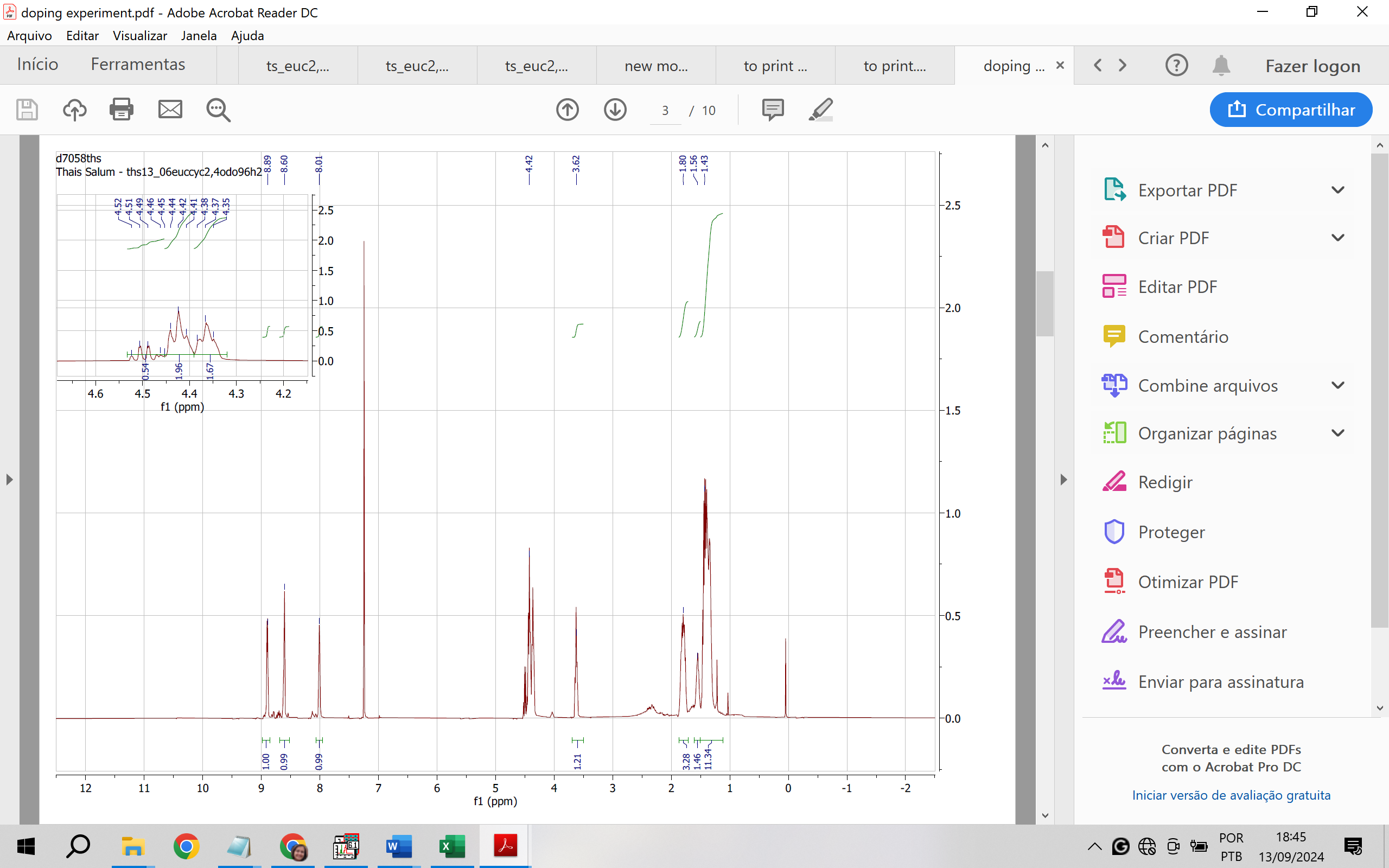


**Supplementary Figure 6.** ^1^H-NMR spectrum of the polymer PD24-ODO synthesized in eucalyptol:cyclohexane 9:1 as a solvent after 96 h (no vacuum) of reaction.


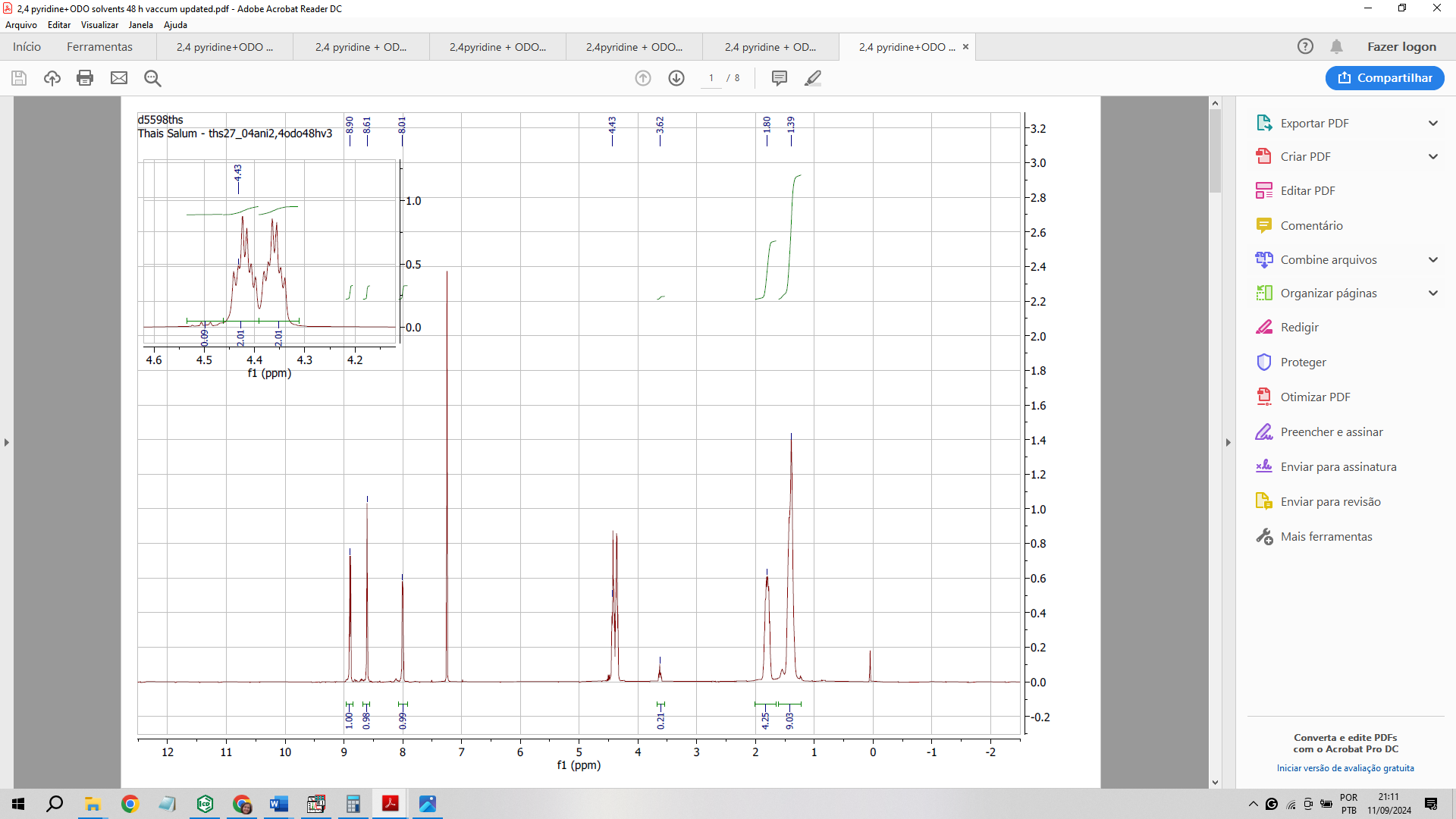


**Supplementary Figure 7.** ^1^H-NMR spectrum of the polymer PD24-ODO synthesized in anisole as a solvent after 48 h (6 h at 1000 mbar + 42 h at 360 mbar) of reaction.


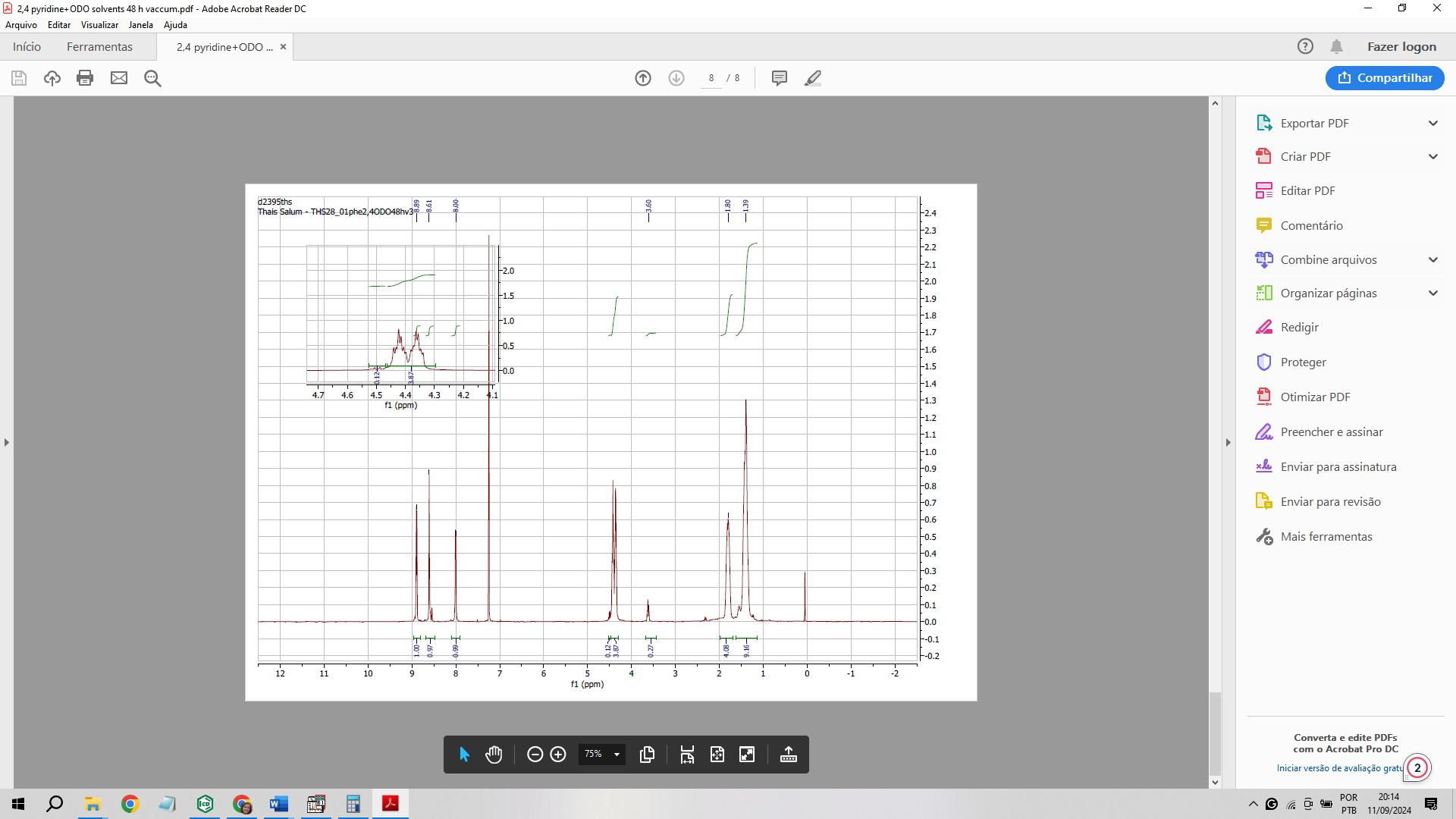


**Supplementary Figure 8.** ^1^H-NMR spectrum of the polymer PD24-ODO synthesized in phenetole as a solvent after 48 h (6 h at 1000 mbar + 42 h at 360 mbar) of reaction.


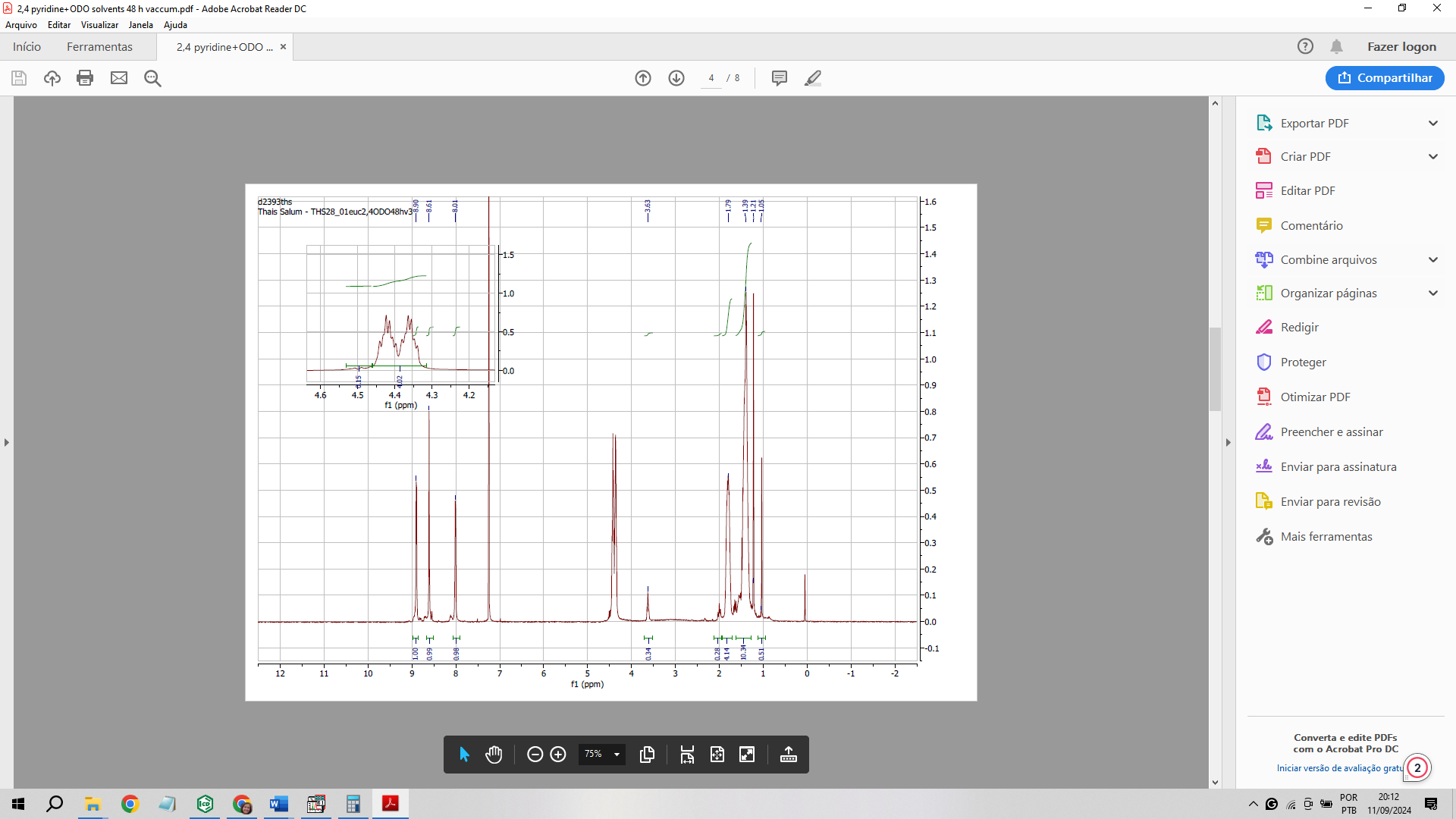


**Supplementary Figure 9.** 1H-NMR spectrum of the polymer PD24-ODO synthesized in eucalyptol as a solvent after 48 h (6 h at 1000 mbar + 42 h at 360 mbar) of reaction.


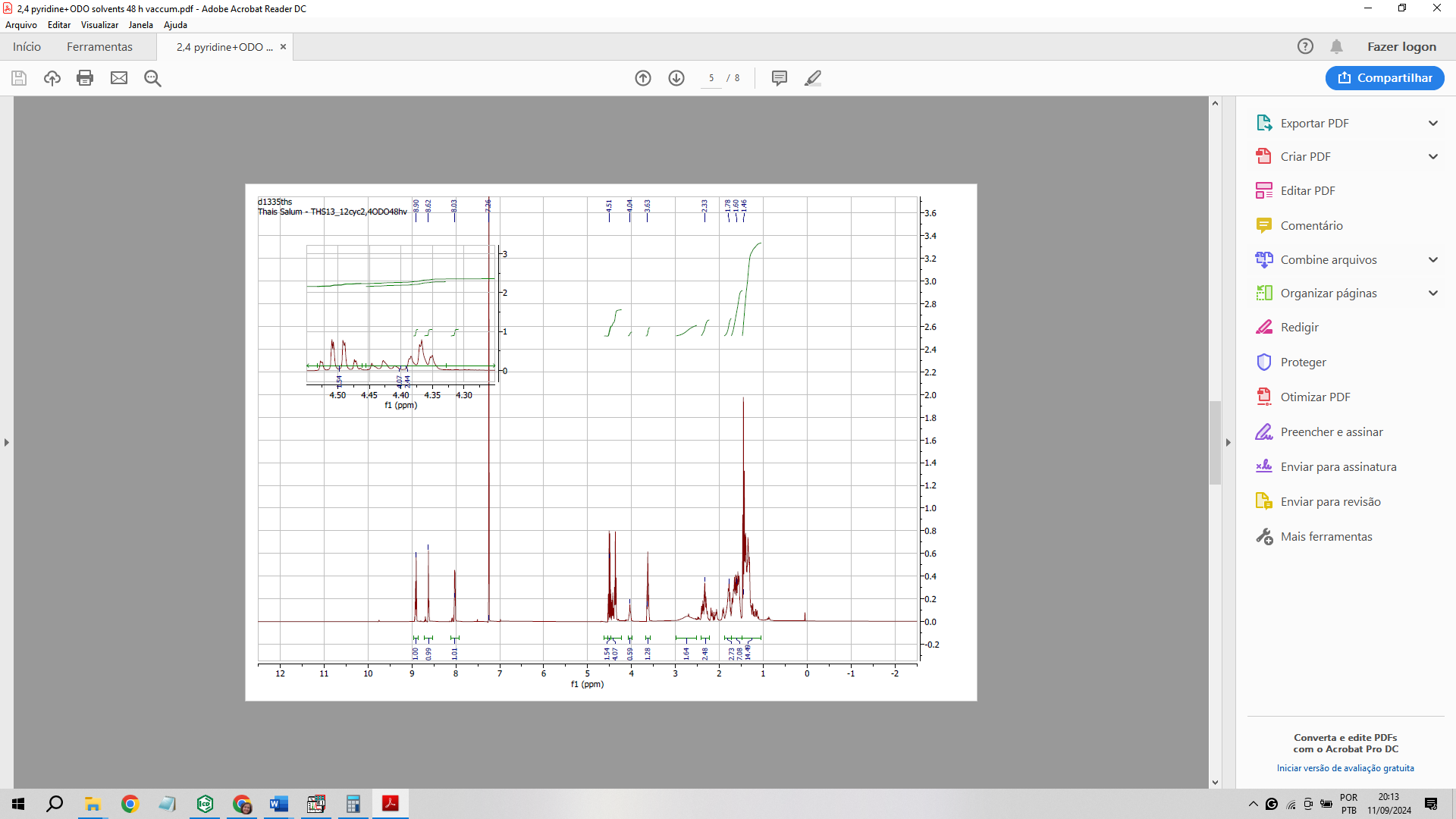


**Supplementary Figure 10.** ^1^H-NMR spectrum of the polymer PD24-ODO synthesized in cyclohexanone as a solvent after 48 h (6 h at 1000 mbar + 42 h at 360 mbar) of reaction.


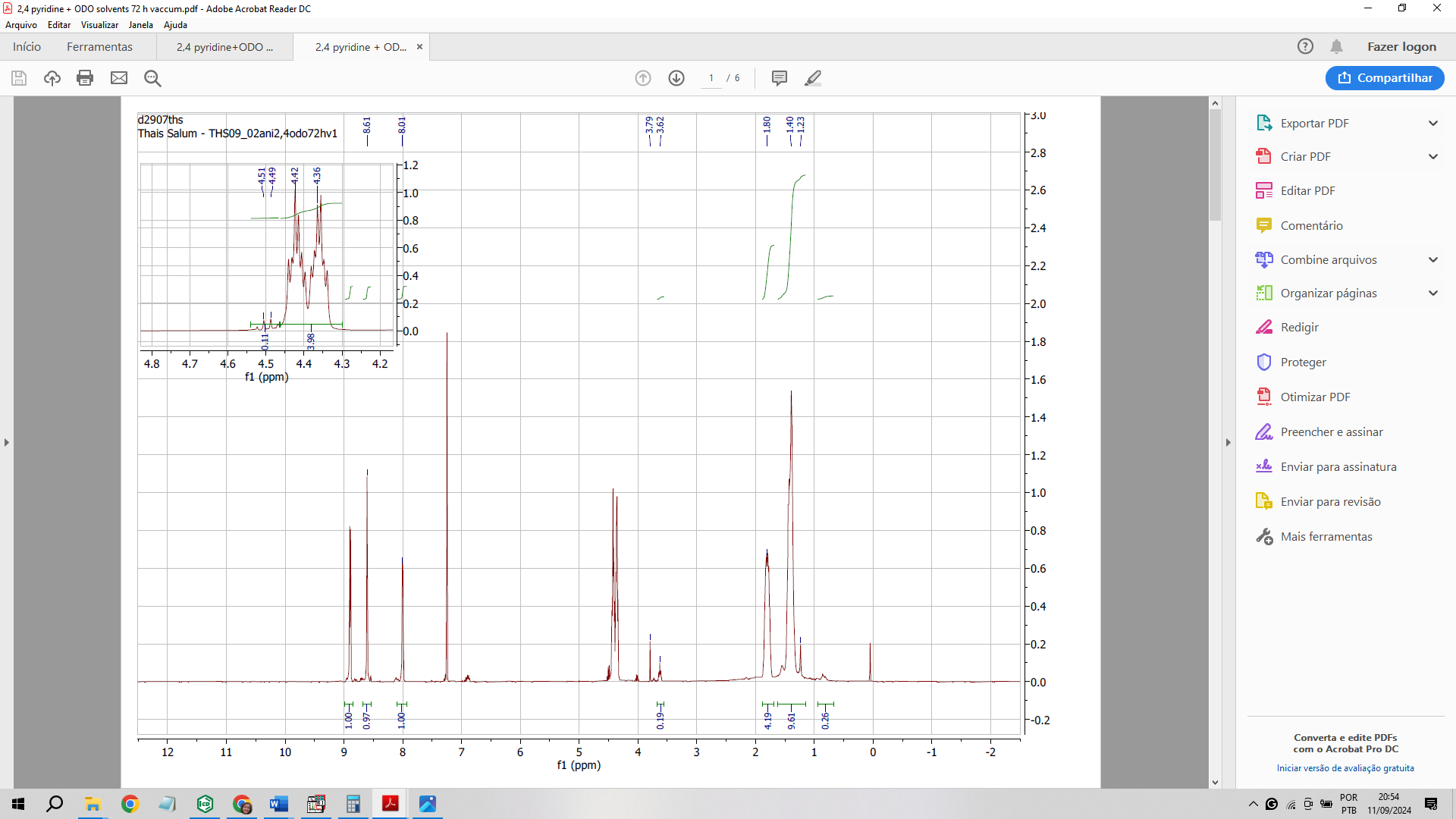


**Supplementary Figure 11.** ^1^H-NMR spectrum of the polymer PD24-ODO synthesized in anisole as a solvent after 72 h (6 h at 1000 mbar + 66 h at 360 mbar) of reaction.


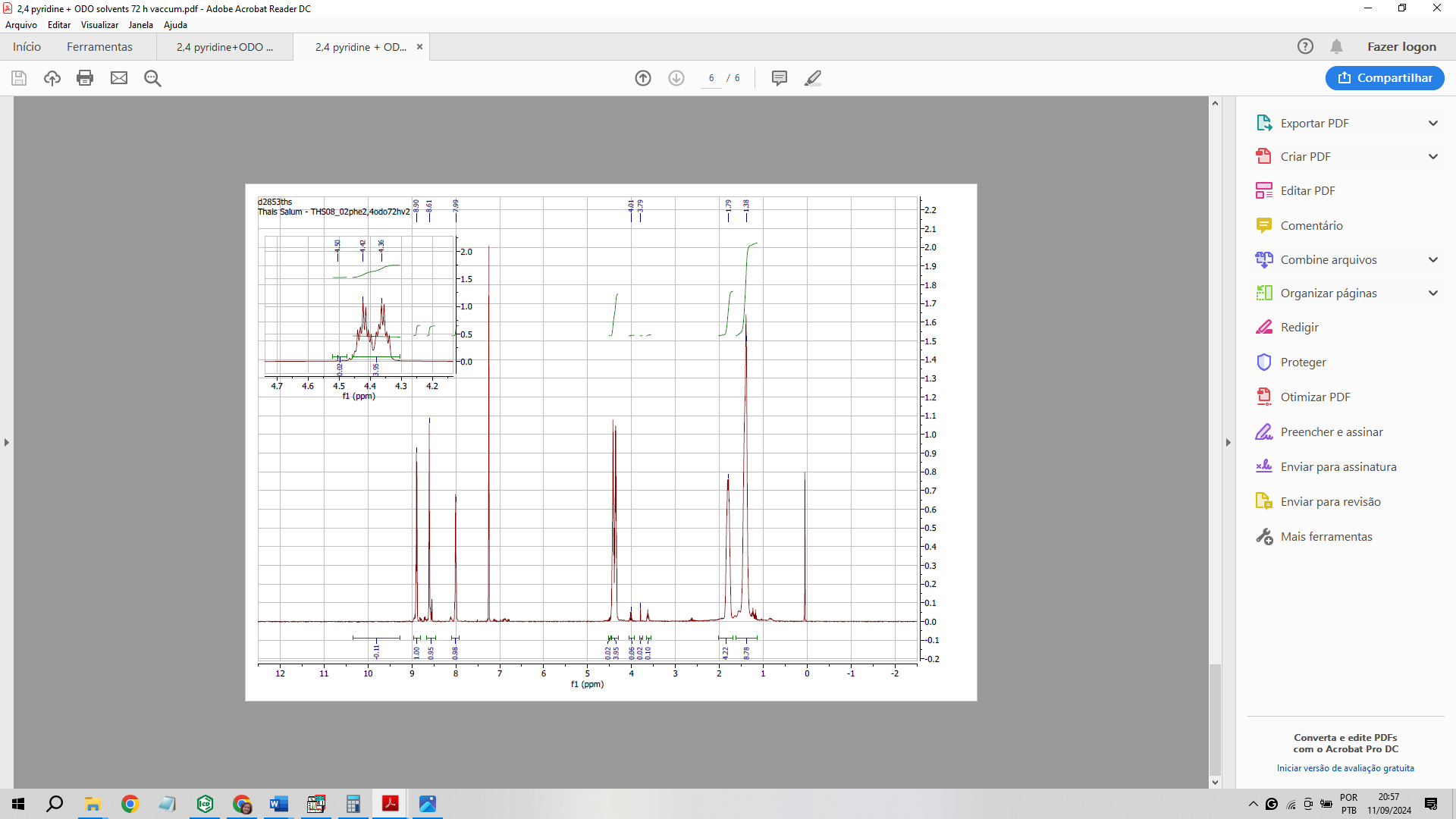


**Supplementary Figure 12.** ^1^H-NMR spectrum of the polymer PD24-ODO synthesized in phenetole as a solvent after 72 h (6 h at 1000 mbar + 66 h at 360 mbar) of reaction.


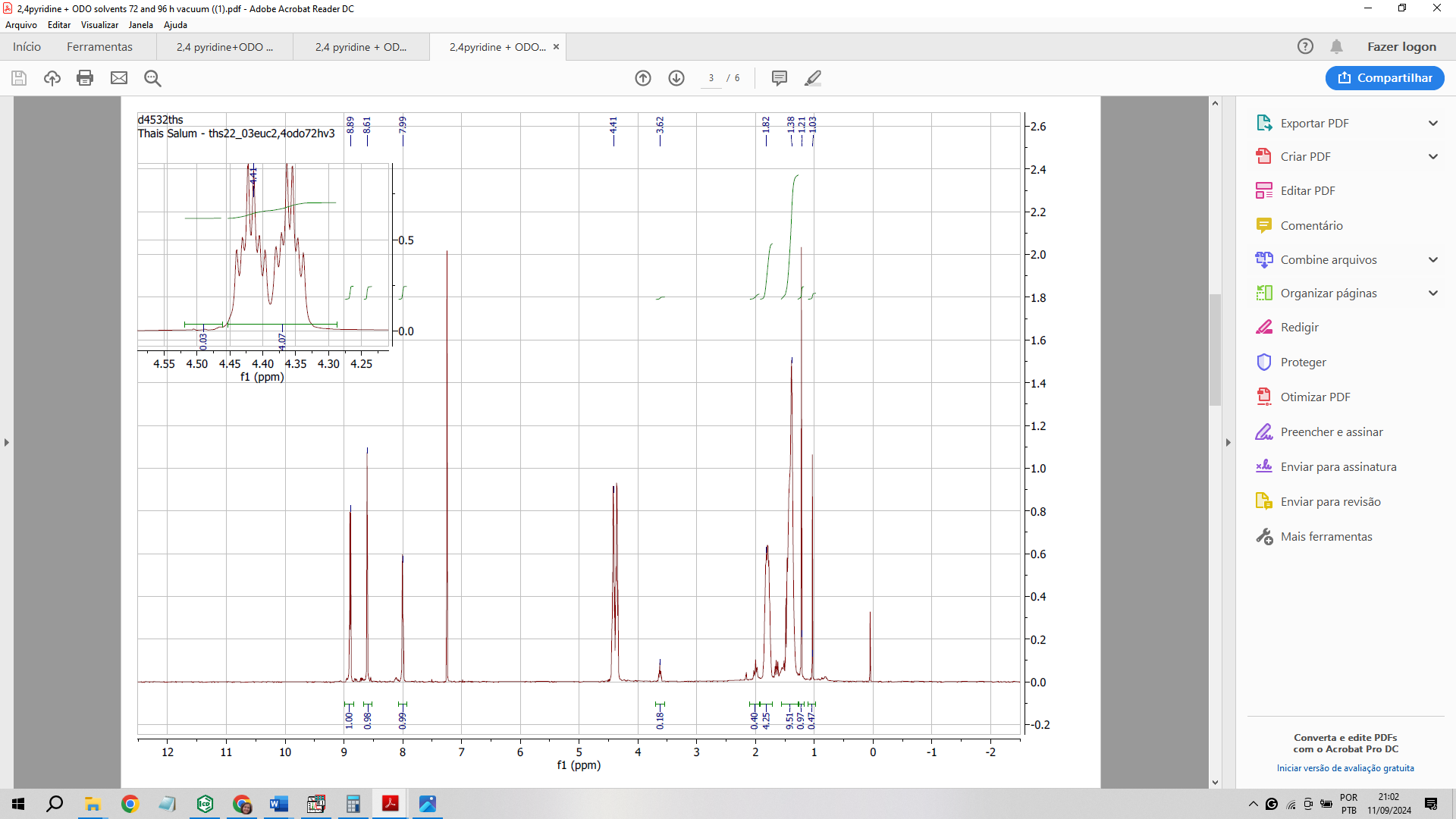


**Supplementary Figure 13.** ^1^H-NMR spectrum of the polymer PD24-ODO synthesized in eucalyptol as a solvent after 72 h (6 h at 1000 mbar + 66 h at 360 mbar) of reaction.


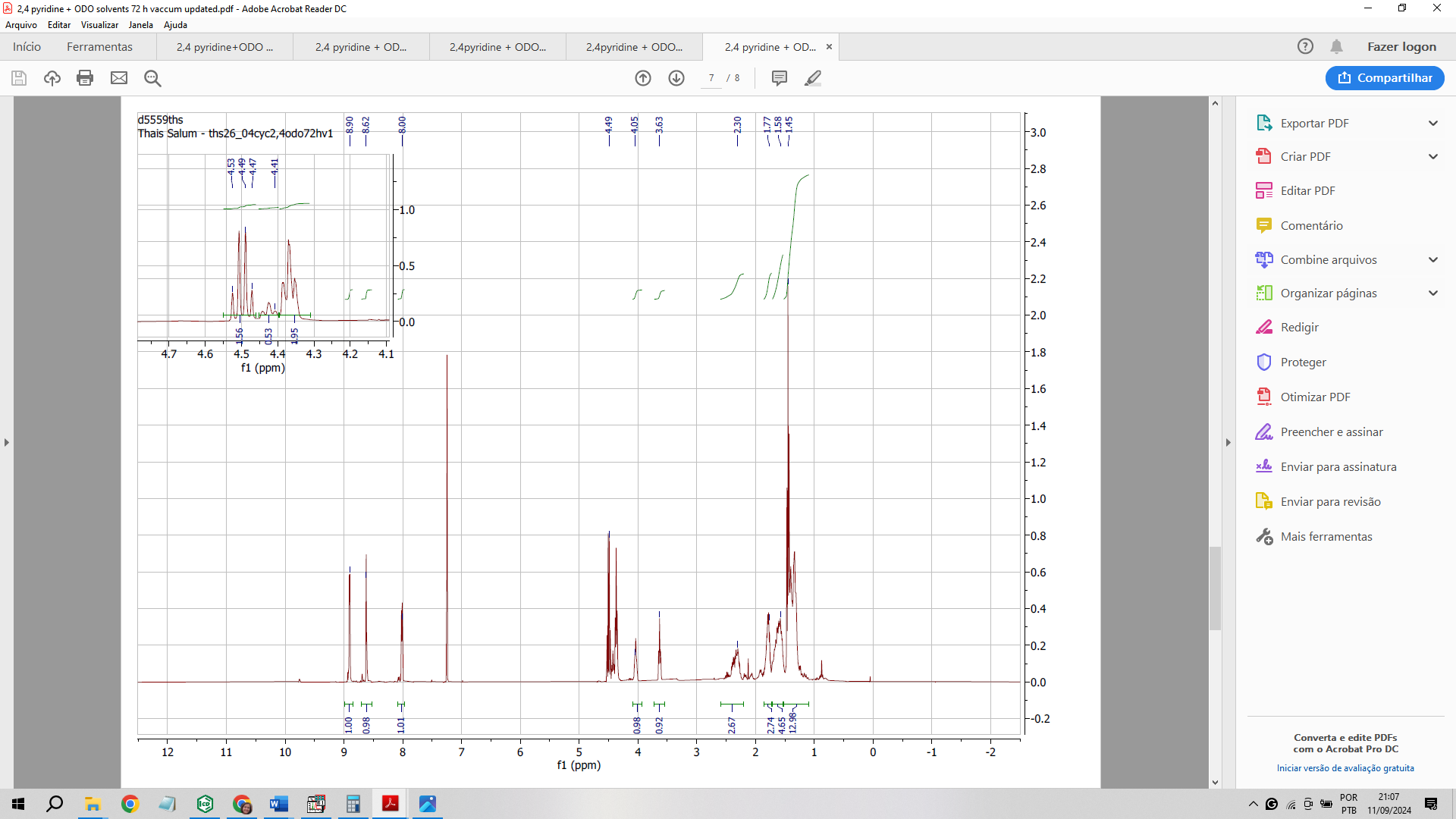


**Supplementary Figure 14.** ^1^H-NMR spectrum of the polymer PD24-ODO synthesized in cyclohexanone as a solvent after 72 h (6 h at 1000 mbar + 66 h at 360 mbar) of reaction.


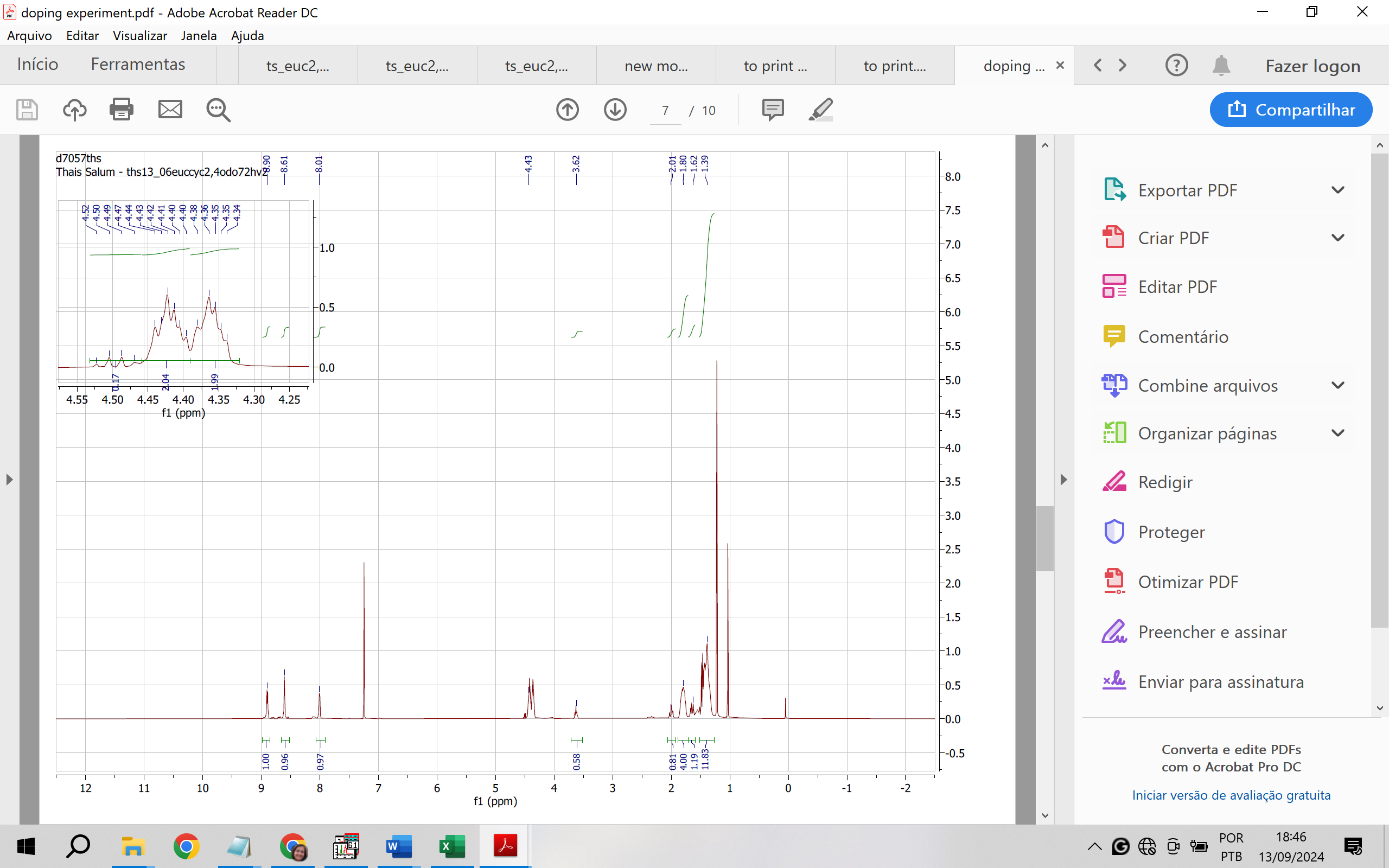


**Supplementary Figure 15.** ^1^H-NMR spectrum of the polymer PD24-ODO synthesized in eucalyptol:cyclohexane 9:1 as a solvent after 72 h (6 h at 1000 mbar + 66 h at 360 mbar) of reaction.


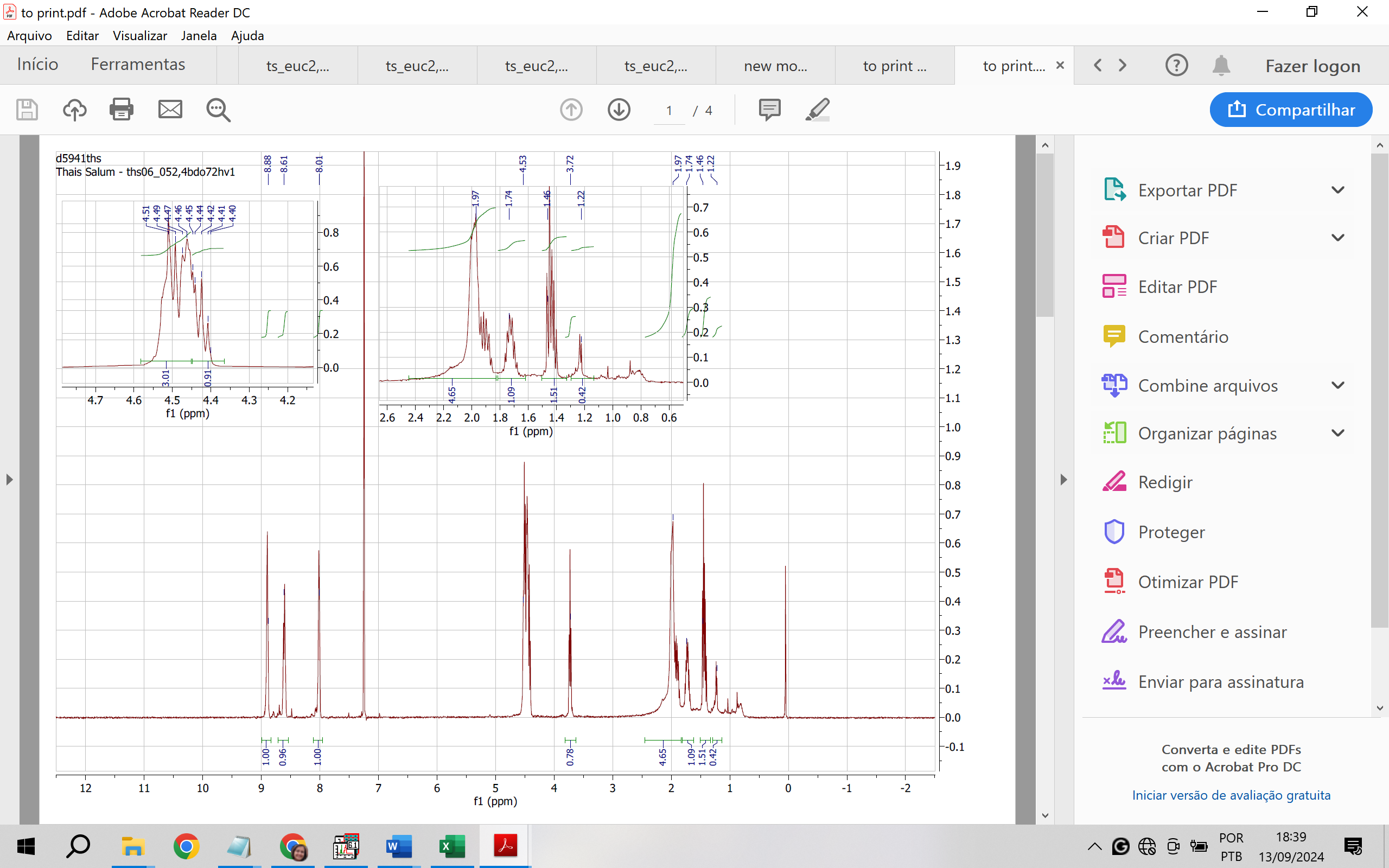


**Supplementary Figure 16.** ^1^H-NMR spectrum of the polymer PD24-BDO synthesized in eucalyptol as a solvent after 72 h (6 h at 1000 mbar + 66 h at 360 mbar) of reaction.


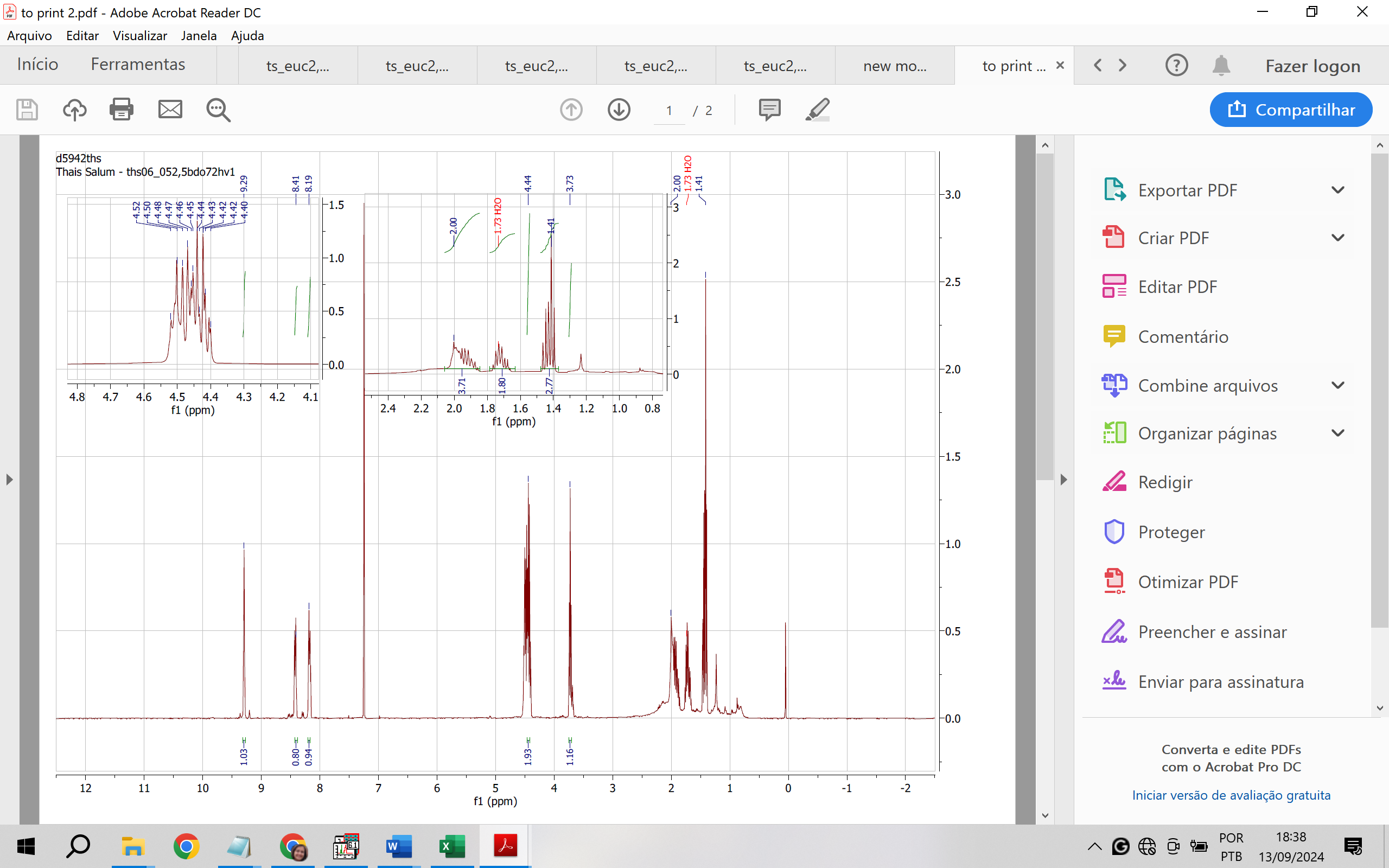


**Supplementary Figure 17.** ^1^H-NMR spectrum of the polymer PD25-BDO synthesized in eucalyptol as a solvent after 72 h (6 h at 1000 mbar + 66 h at 360 mbar) of reaction.


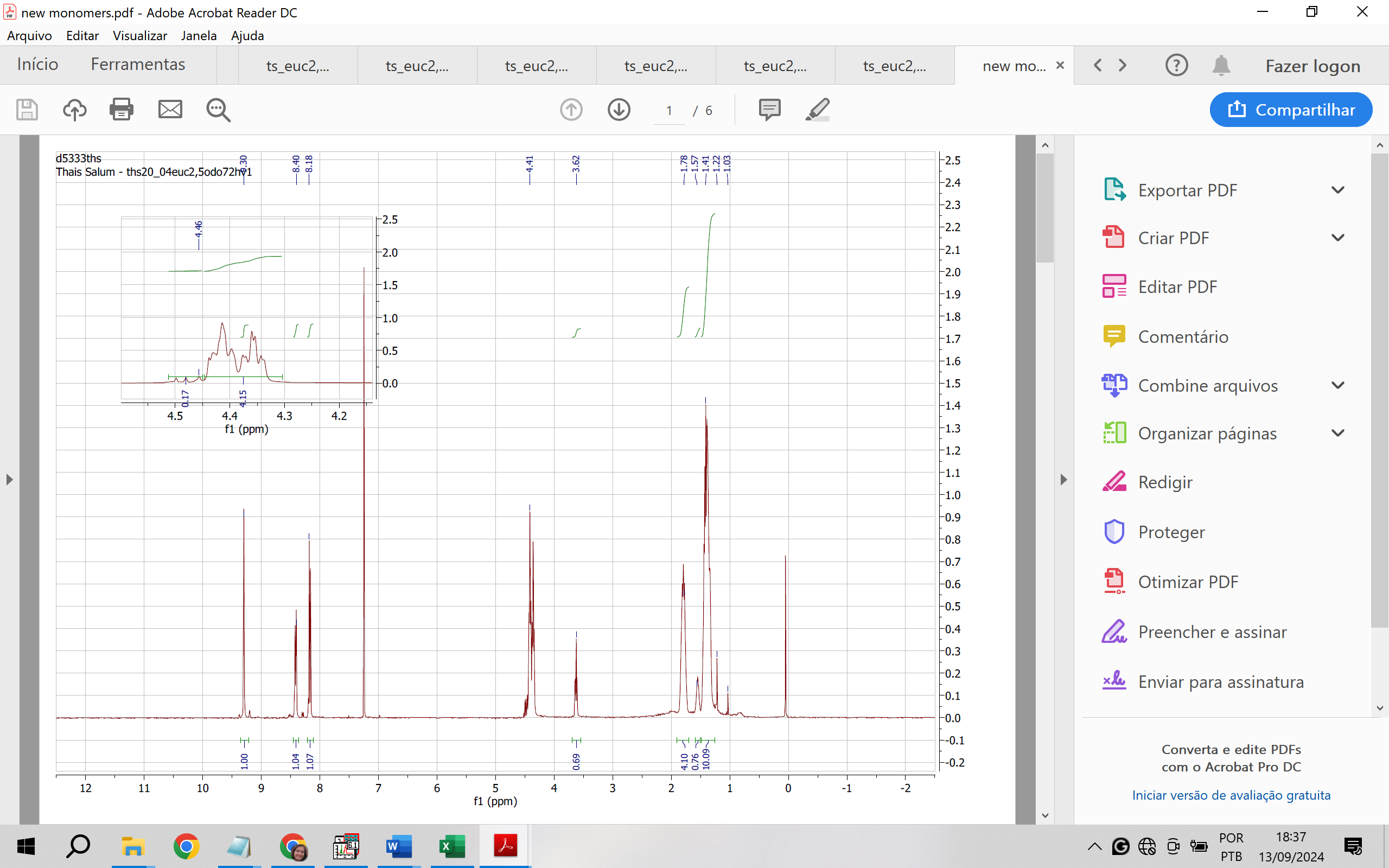


**Supplementary Figure 18.** ^1^H-NMR spectrum of the polymer PD25-ODO synthesized in eucalyptol as a solvent after 72 h (6 h at 1000 mbar + 66 h at 360 mbar) of reaction.


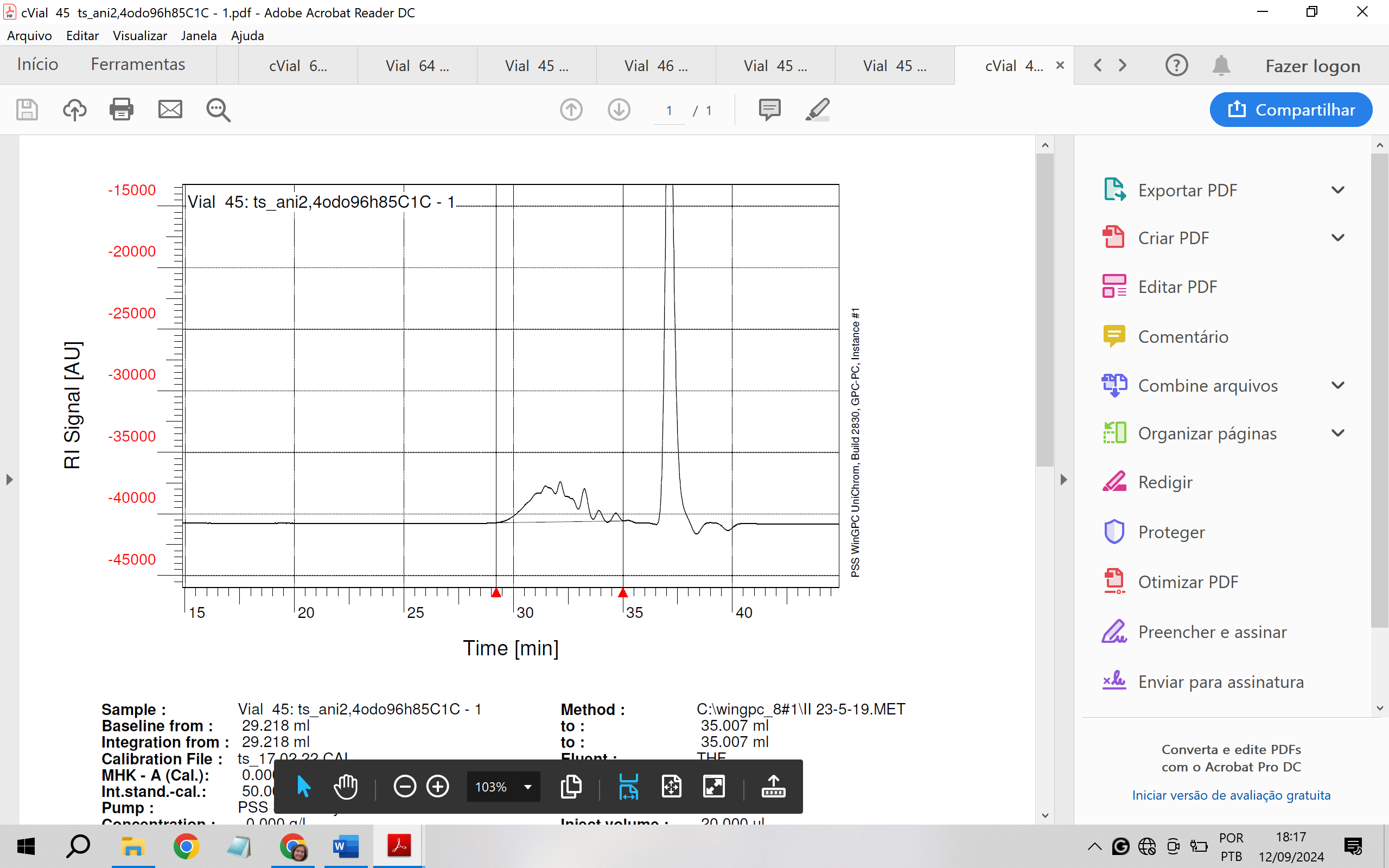
**Supplementary Figure 19.** GPC chromatogram of the polymer PD24-ODO synthesized in anisole as a solvent after 96 h (no vacuum) of reaction.


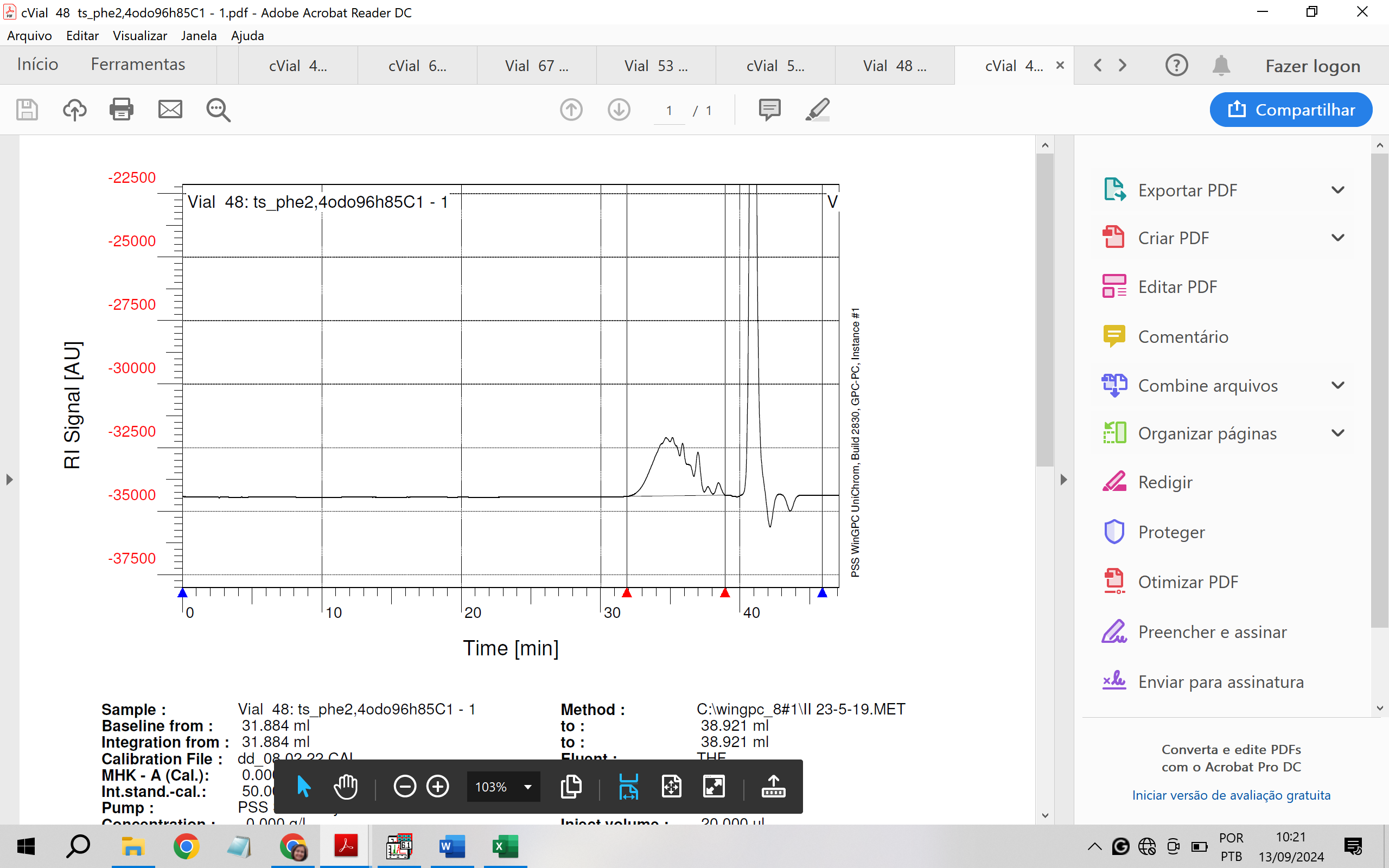


**Supplementary Figure 20.** GPC chromatogram of the polymer PD24-ODO synthesized in phenetole as a solvent after 96 h (no vacuum) of reaction.


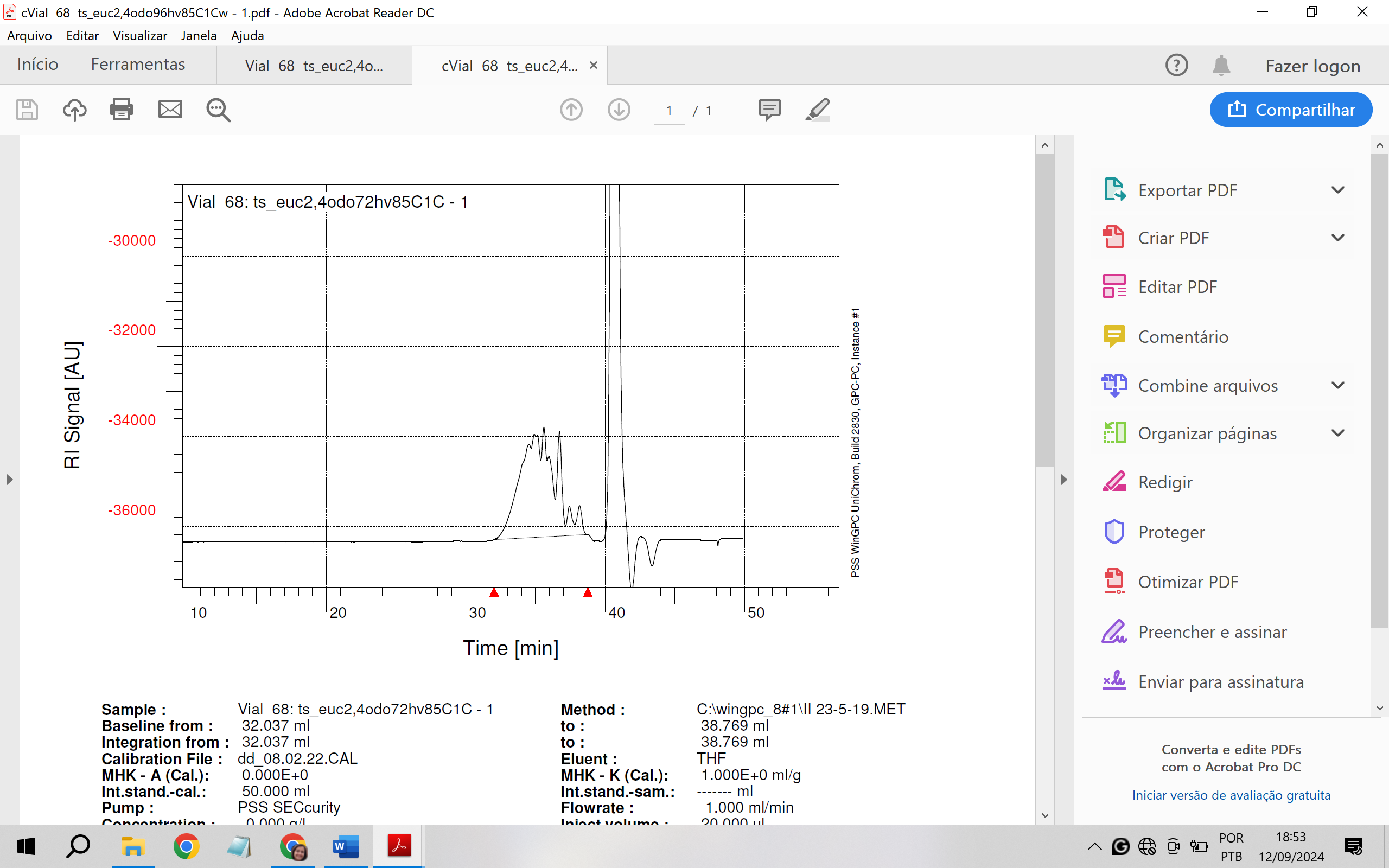


**Supplementary Figure 21.** GPC chromatogram of the polymer PD24-ODO synthesized in eucalyptol as a solvent after 96 h (no vacuum) of reaction.


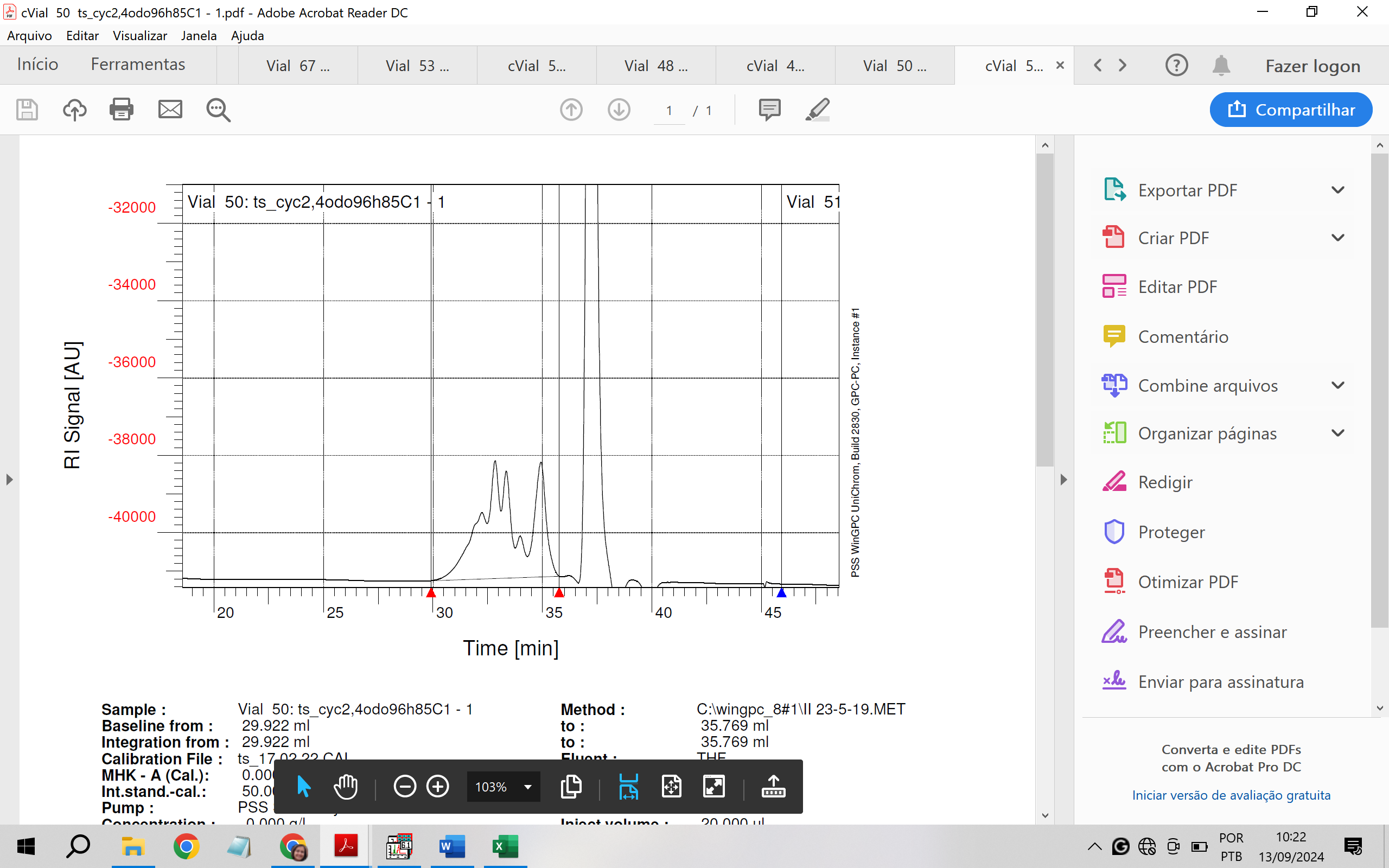


**Supplementary Figure 22.** GPC chromatogram of the polymer PD24-ODO synthesized in cyclohexanone as a solvent after 96 h (no vacuum) of reaction.


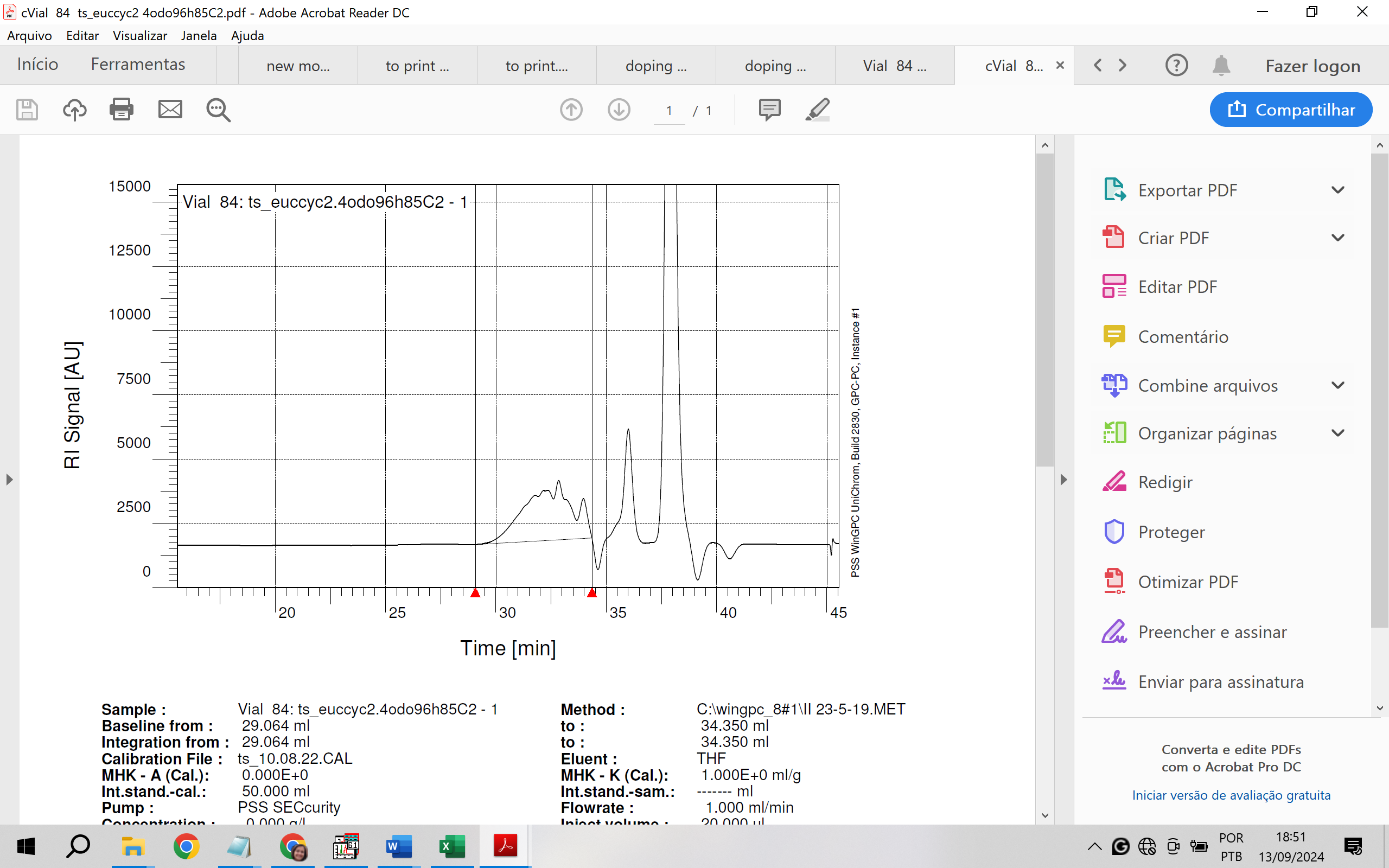


**Supplementary Figure 23.** GPC chromatogram of the polymer PD24-ODO synthesized in eucalyptol:cyclohexane 9:1 as a solvent after 96 h (no vacuum) of reaction.


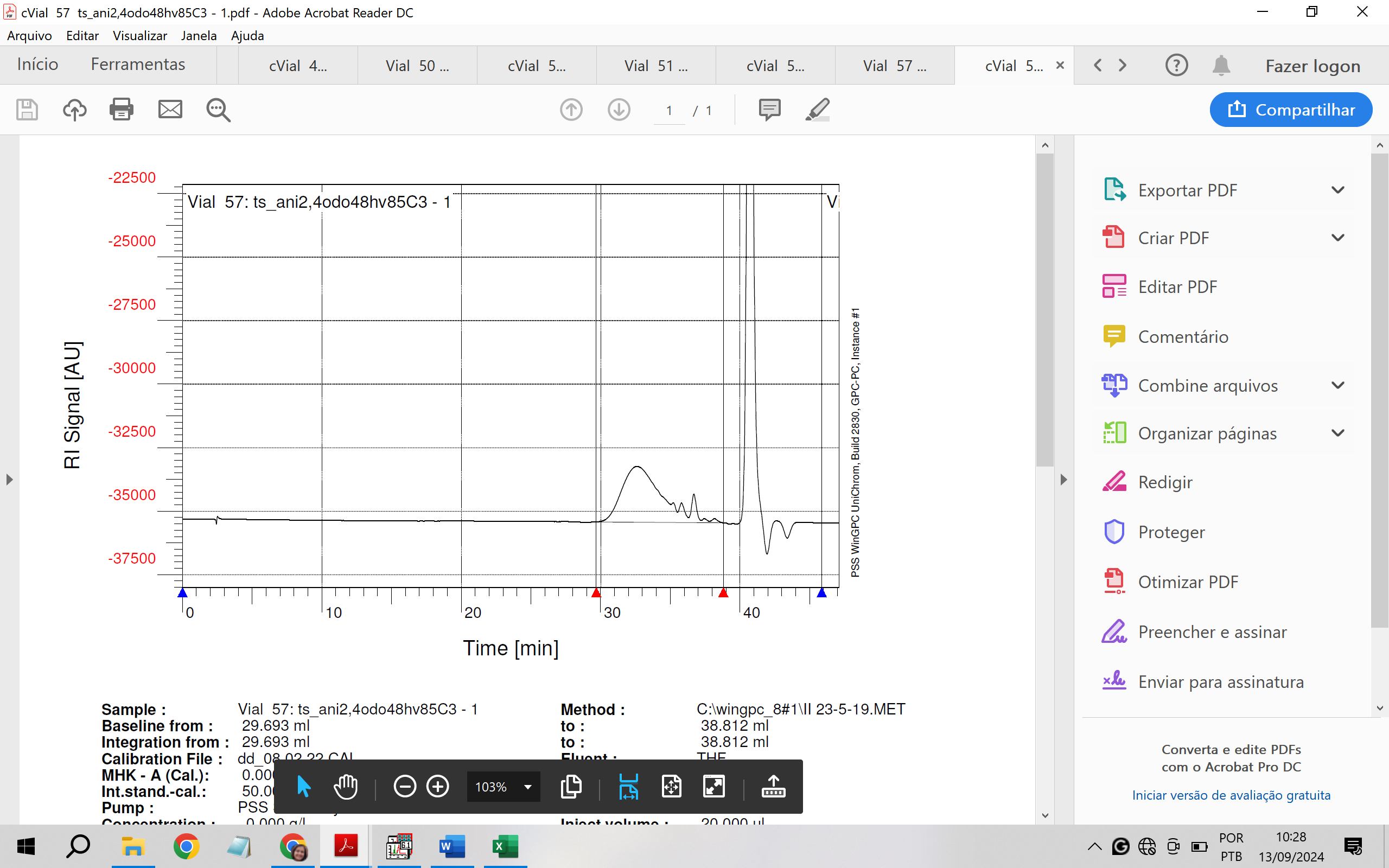


**Supplementary Figure 24.** GPC chromatogram of the polymer PD24-ODO synthesized in anisole as a solvent after 48 h (6 h at 1000 mbar + 42 h at 360 mbar) of reaction.


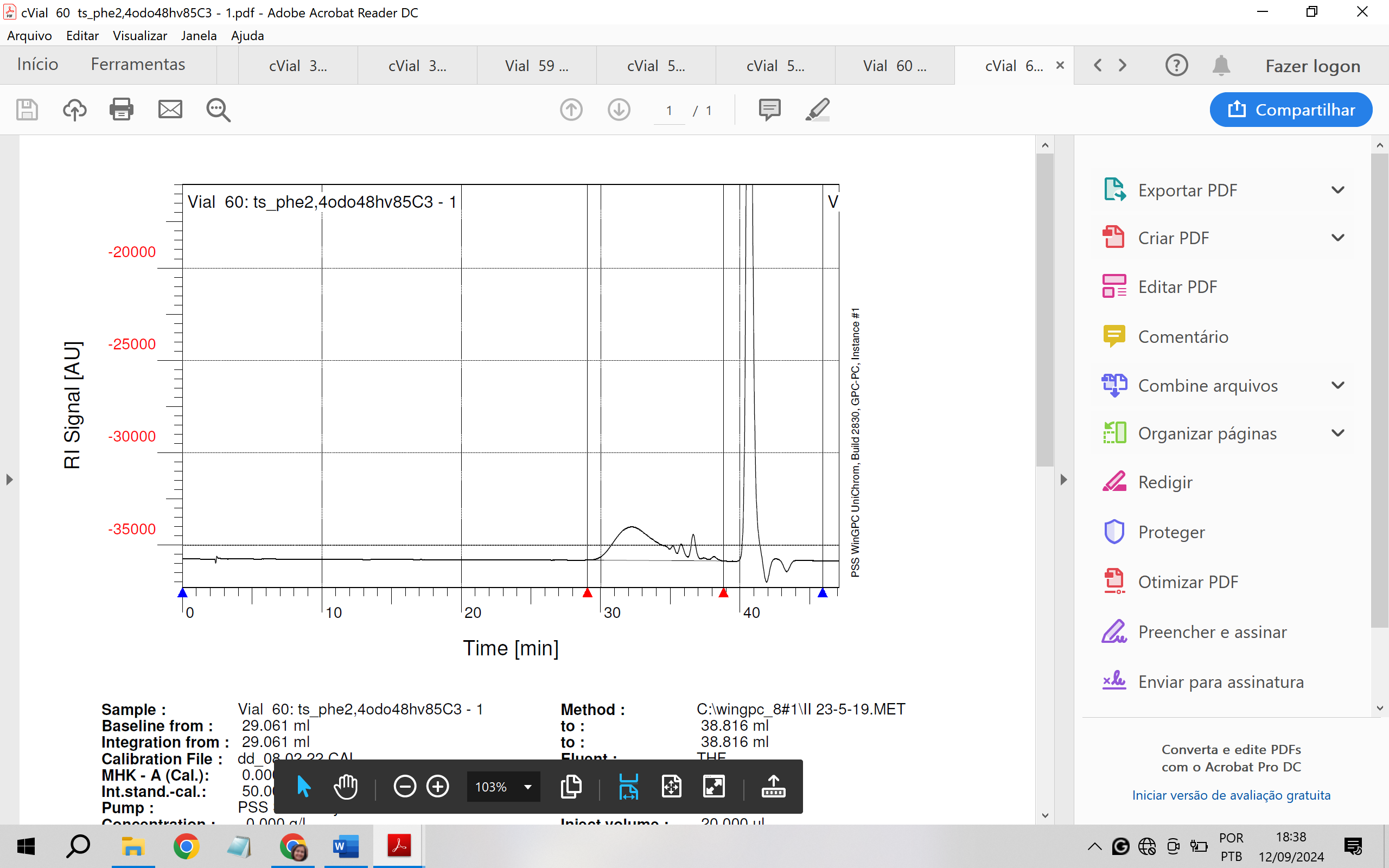


**Supplementary Figure 25.** GPC chromatogram of the polymer PD24-ODO synthesized in phenetole as a solvent after 48 h (6 h at 1000 mbar + 42 h at 360 mbar) of reaction.


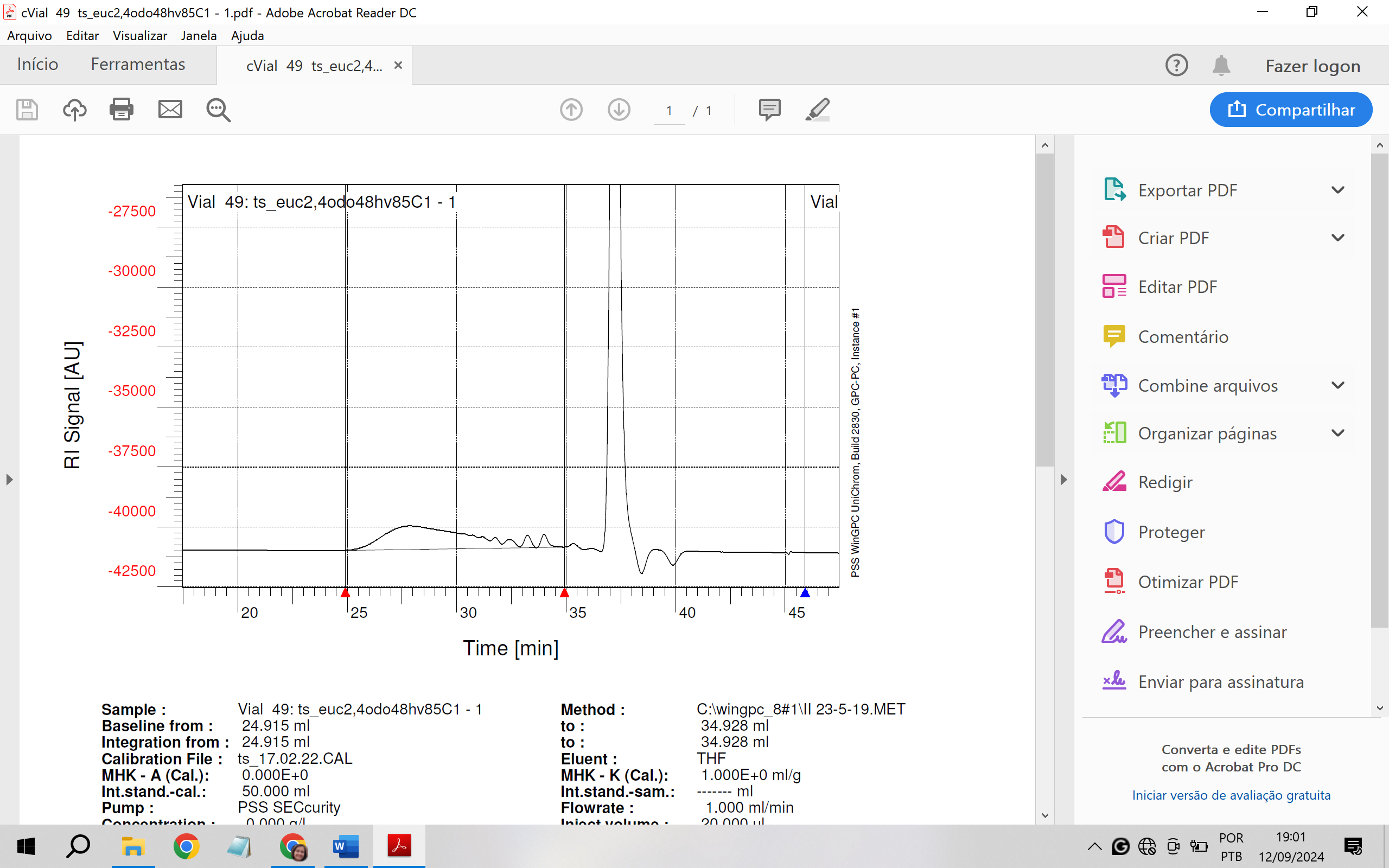


**Supplementary Figure 26.** GPC chromatogram of the polymer PD24-ODO synthesized in eucalyptol as a solvent after 48 h (6 h at 1000 mbar + 42 h at 360 mbar) of reaction.


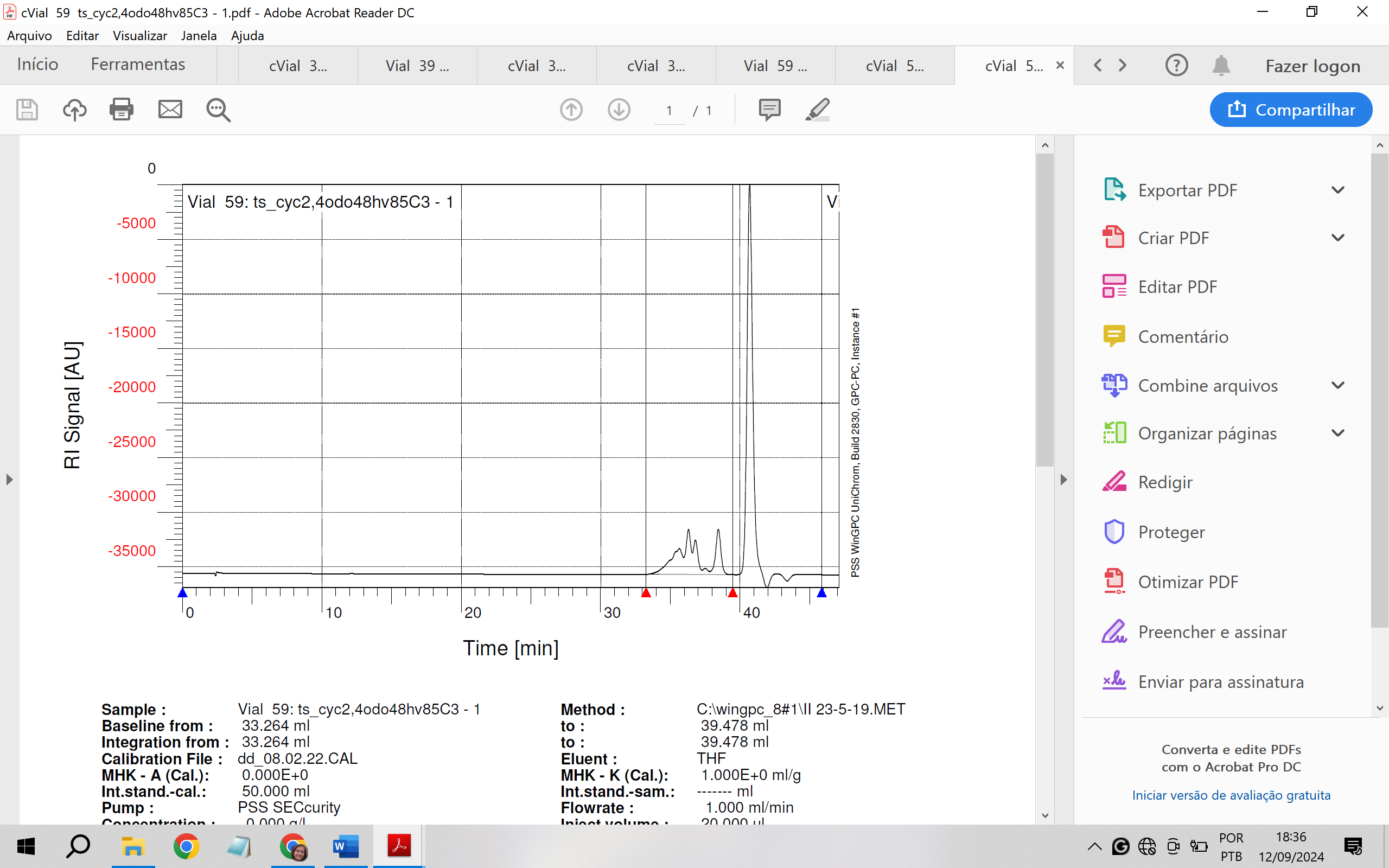


**Supplementary Figure 27.** GPC chromatogram of the polymer PD24-ODO synthesized in cyclohexanone as a solvent after 48 h (6 h at 1000 mbar + 42 h at 360 mbar) of reaction.


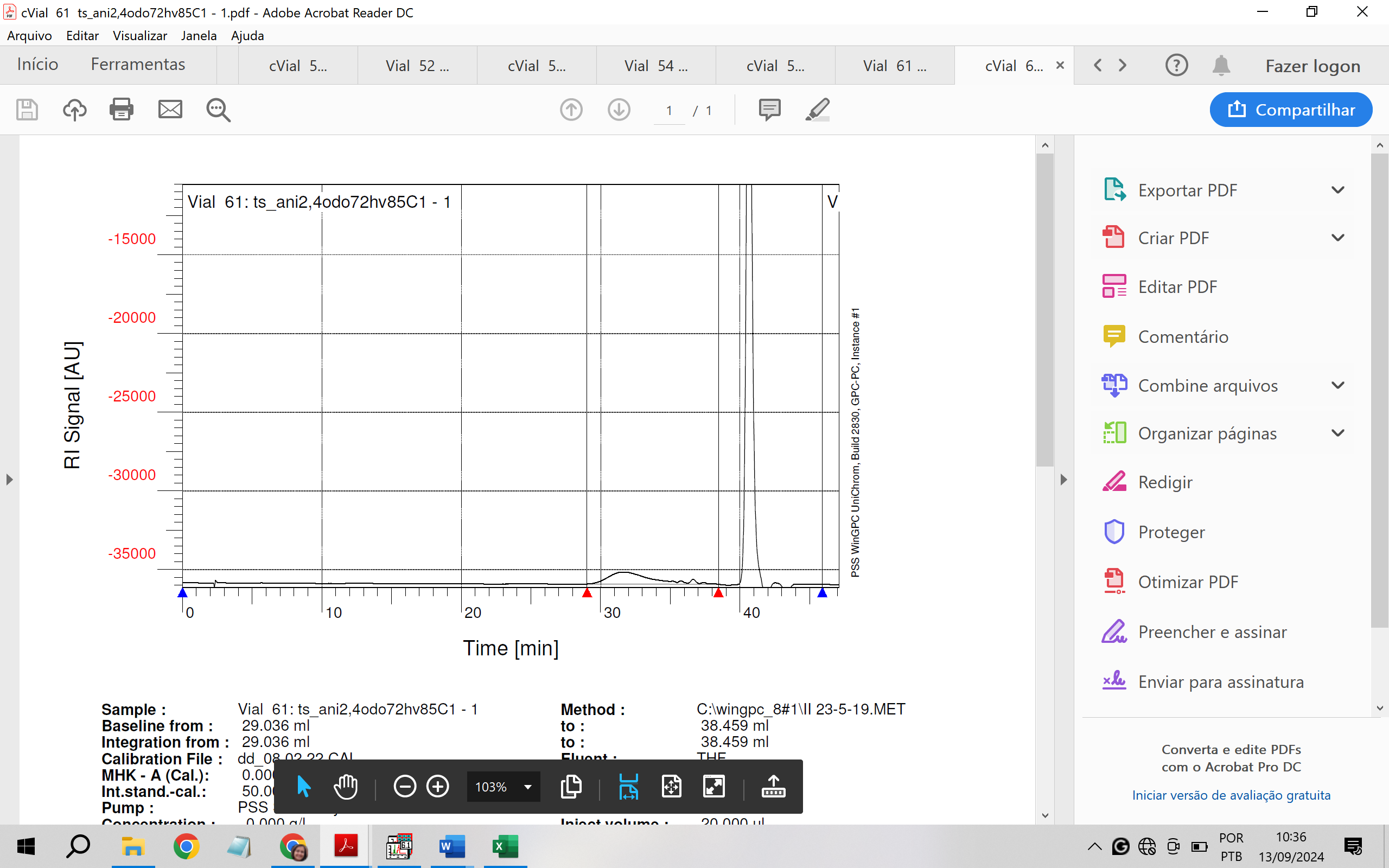


**Supplementary Figure 28.** GPC chromatogram of the polymer PD24-ODO synthesized in anisole as a solvent after 72 h (6 h at 1000 mbar + 66 h at 360 mbar) of reaction.


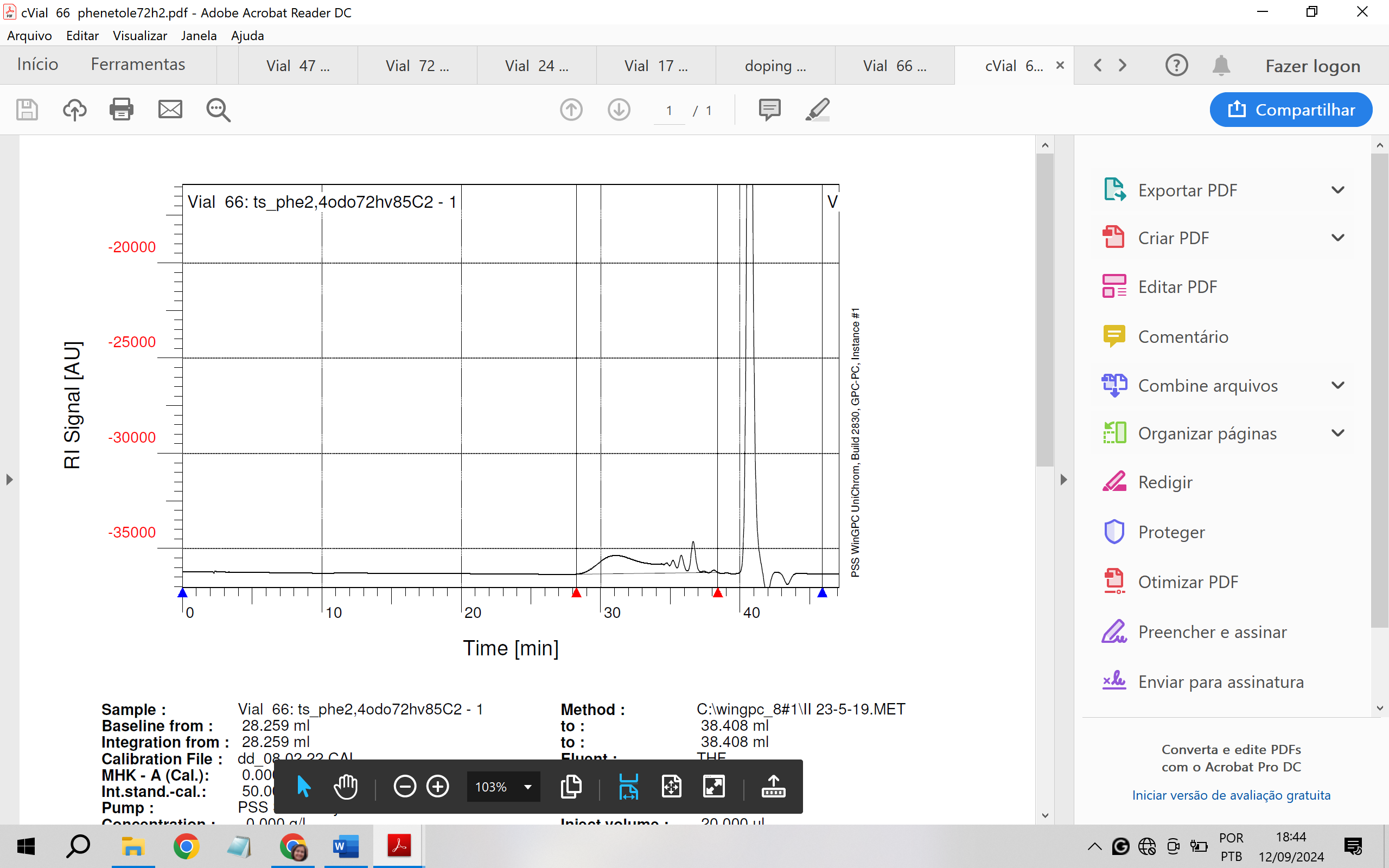


**Supplementary Figure 29.** GPC chromatogram of the polymer PD24-ODO synthesized in phenetole as a solvent after 72 h (6 h at 1000 mbar + 66 h at 360 mbar) of reaction.


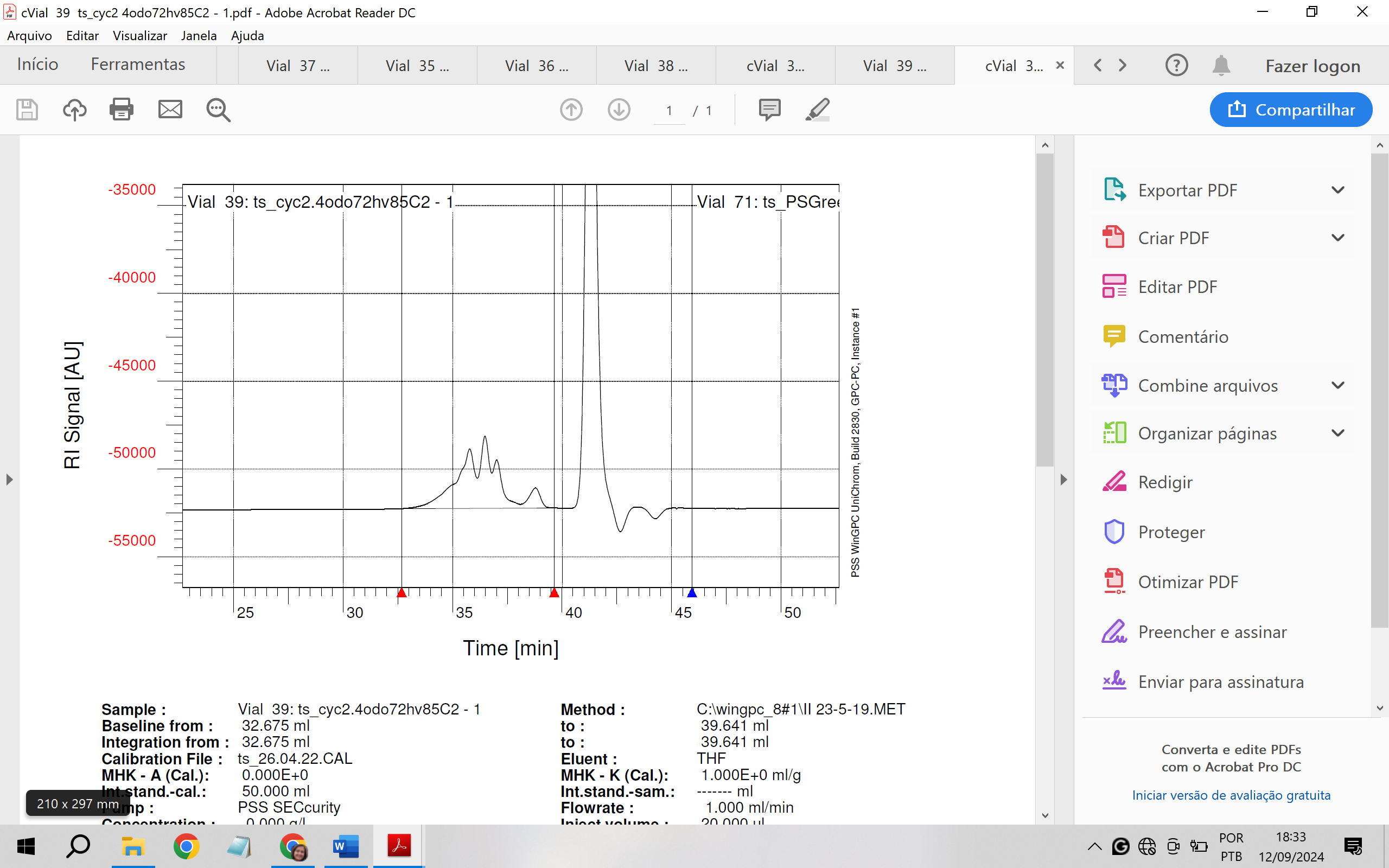


**Supplementary Figure 30.** GPC chromatogram of the polymer PD24-ODO synthesized in cyclohexanone as a solvent after 72 h (6 h at 1000 mbar + 66 h at 360 mbar) of reaction.


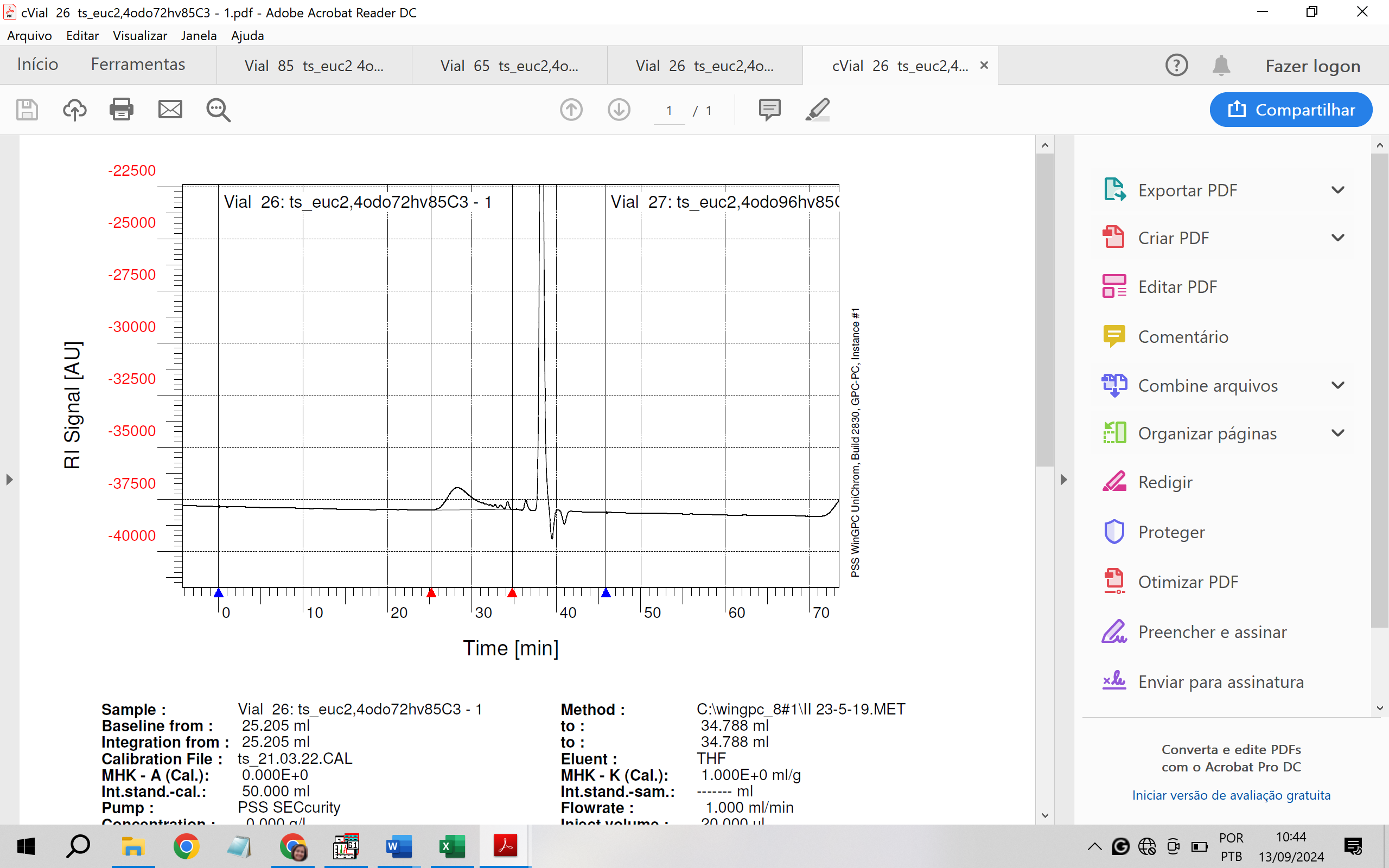


**Supplementary Figure 31.** GPC chromatogram of the polymer PD24-ODO synthesized in eucalyptol as a solvent after 72 h (6 h at 1000 mbar + 66 h at 360 mbar) of reaction.


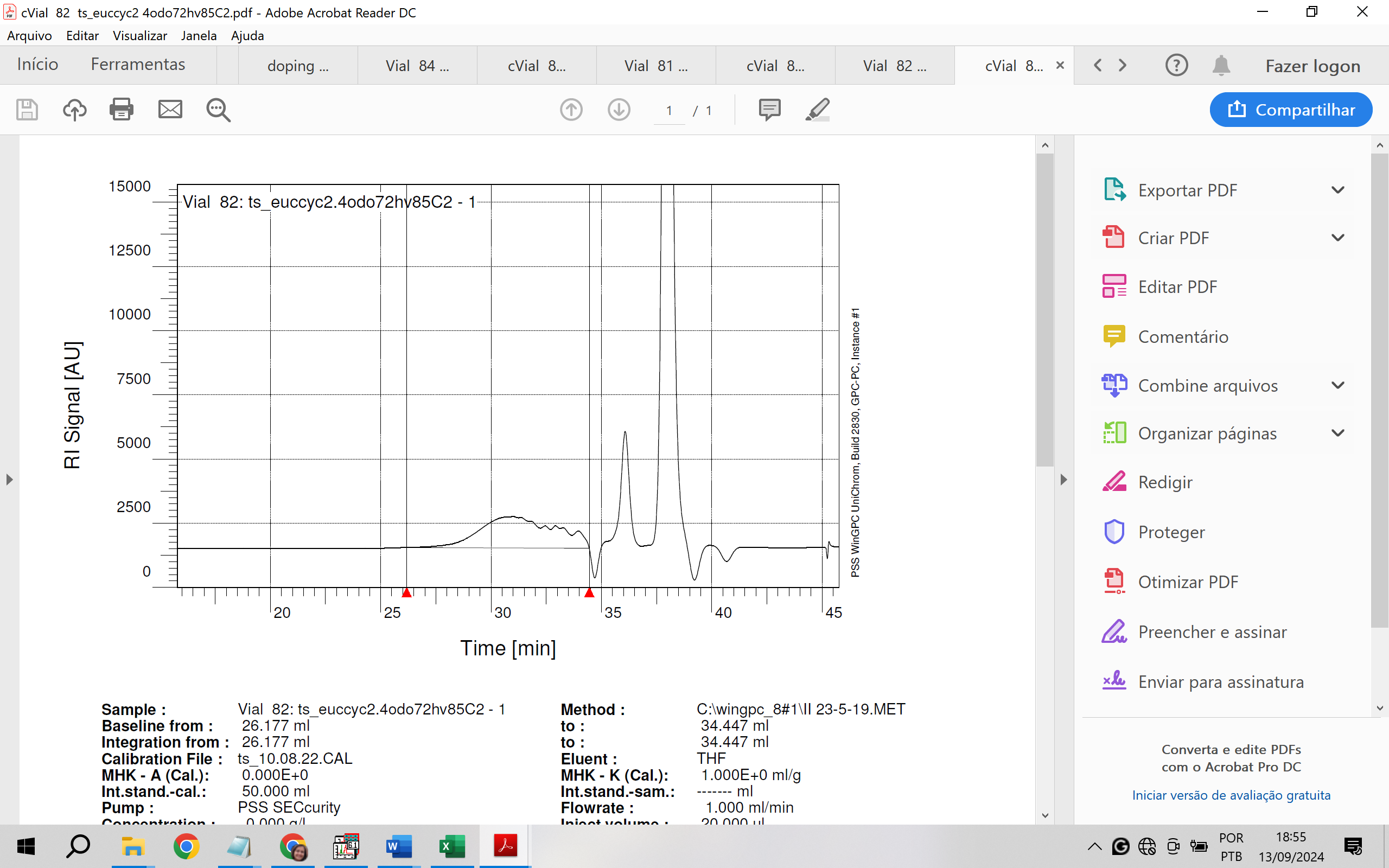


**Supplementary Figure 32.** GPC chromatogram of the polymer PD24-ODO synthesized in eucalyptol:cyclohexanone 9:1 as a solvent after 72 h (6 h at 1000 mbar + 66 h at 360 mbar) of reaction


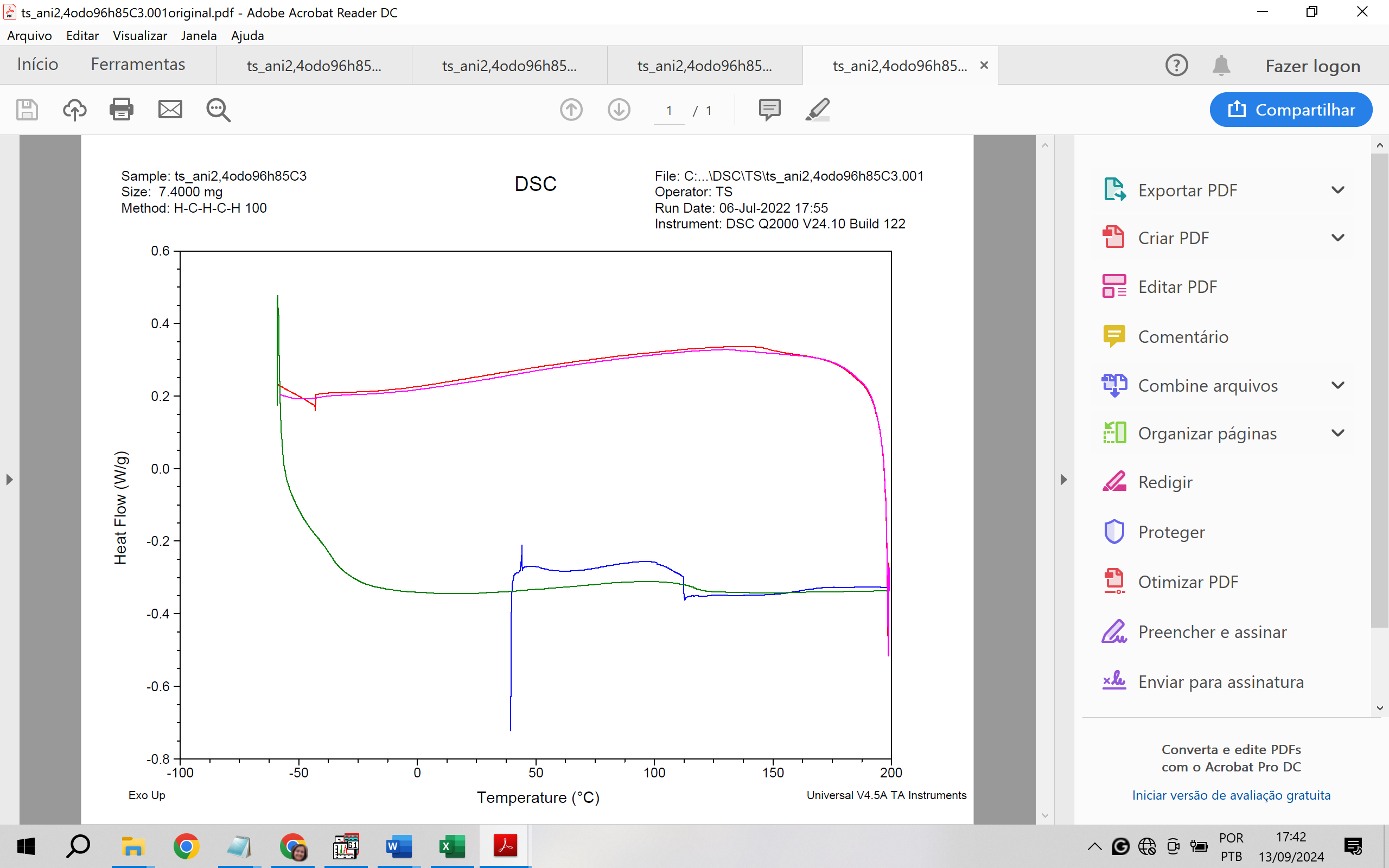


**Supplementary Figure 33.** DSC thermogram of the polymer PD24-ODO synthesised in anisole as a solvent in 96 h reaction (no vaccum).


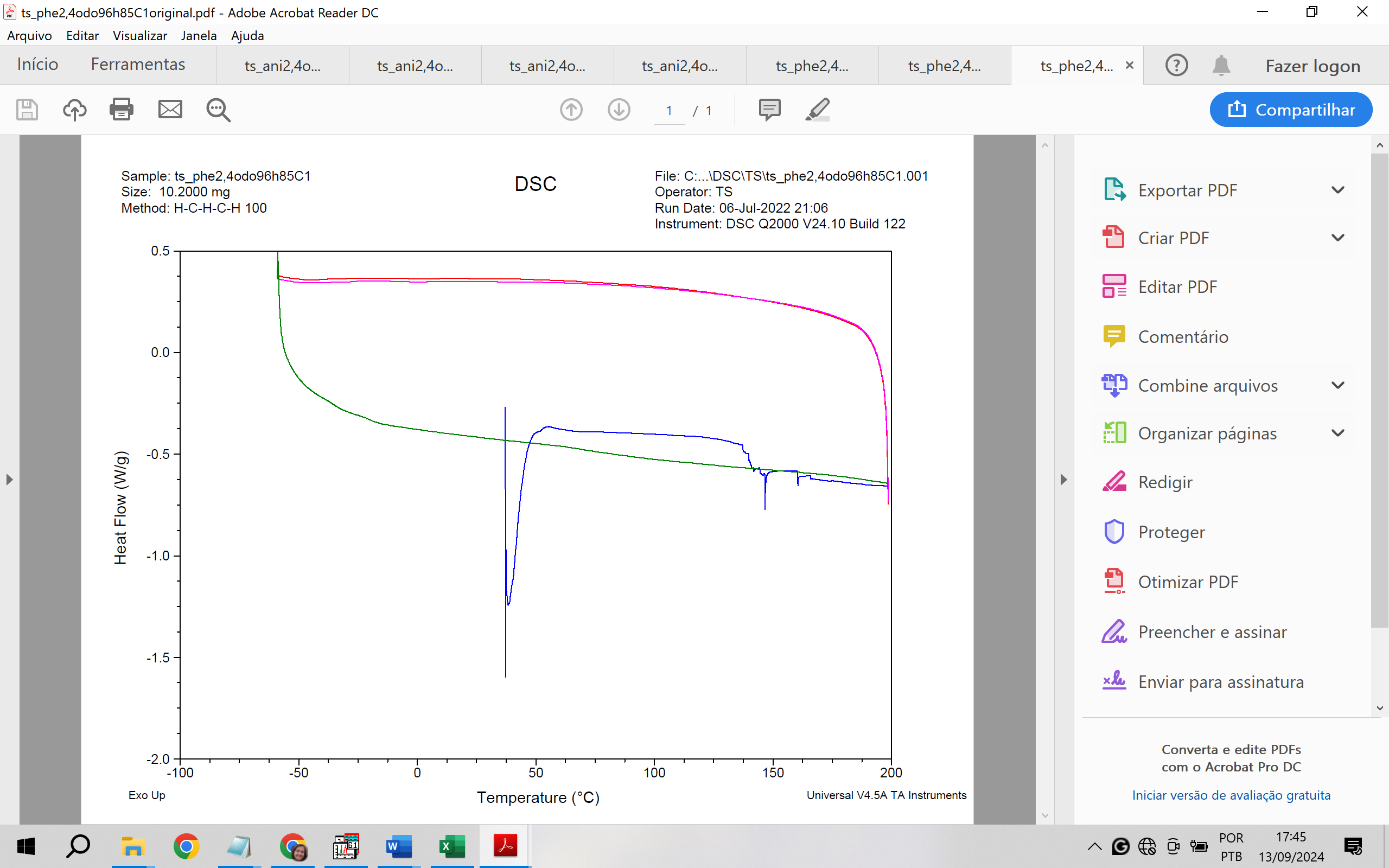


**Supplementary Figure 34.** DSC thermogram of the polymer PD24-ODO synthesised in phenetole as a solvent in 96 h reaction (no vaccum).


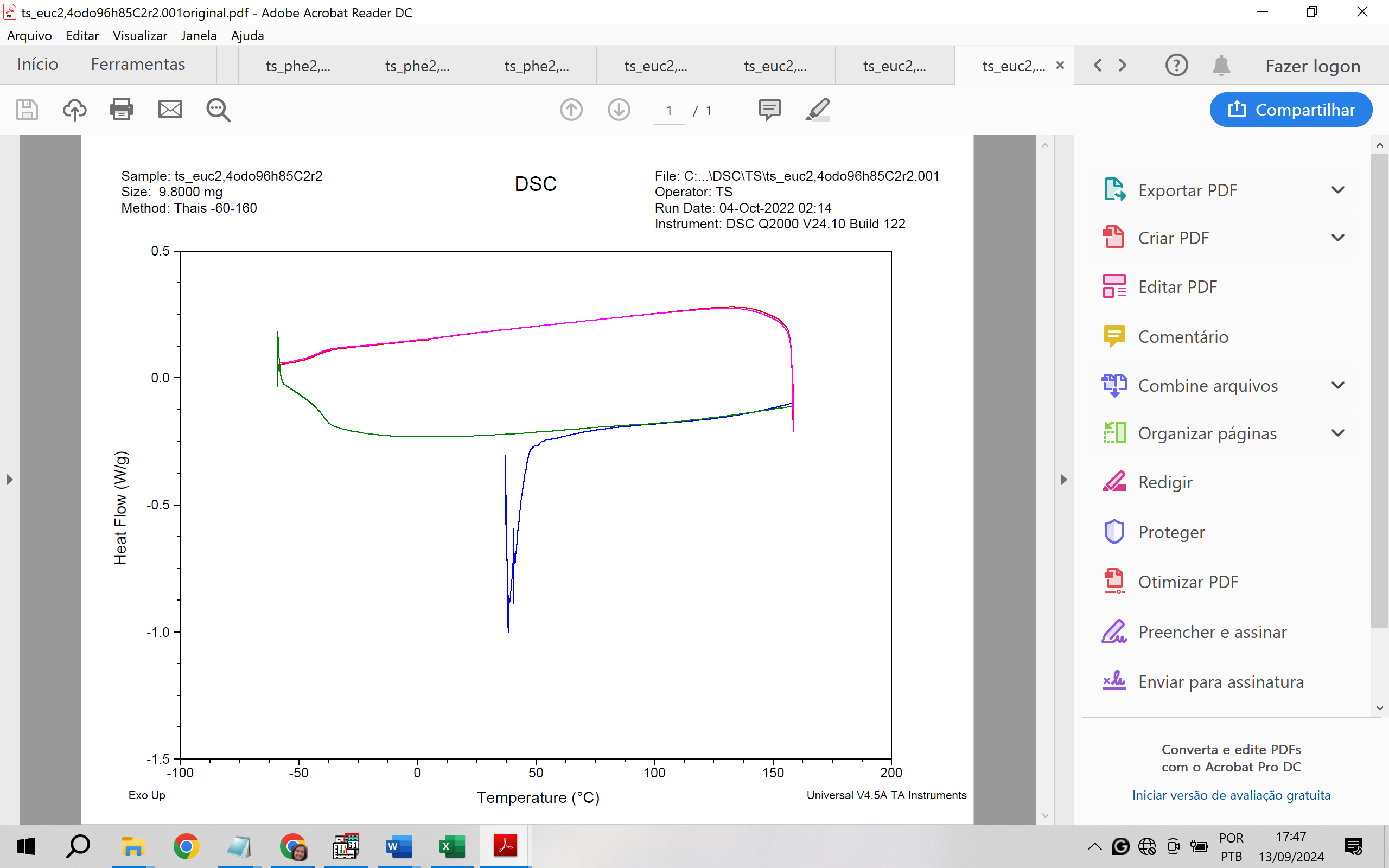


**Supplementary Figure 35.** DSC thermogram of the polymer PD24-ODO synthesised in eucalyptol as a solvent after 96 h (no vaccum) of reaction.


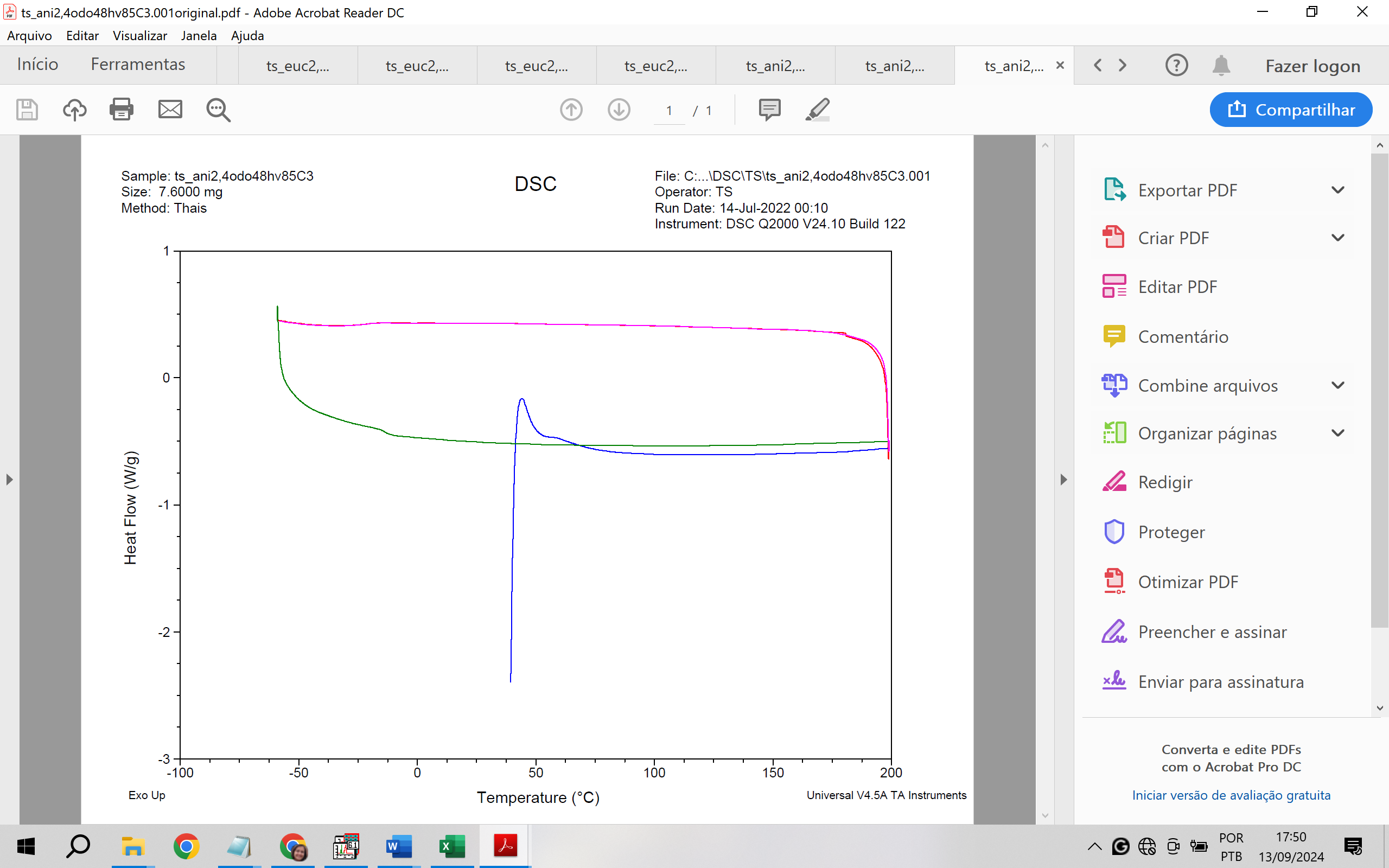


**Supplementary Figure 36.** DSC thermogram of the polymer PD24-ODO synthesised in anisole as a solvent after 48 h (6 h at 1000 mbar + 42 h at 360 mbar) of reaction.


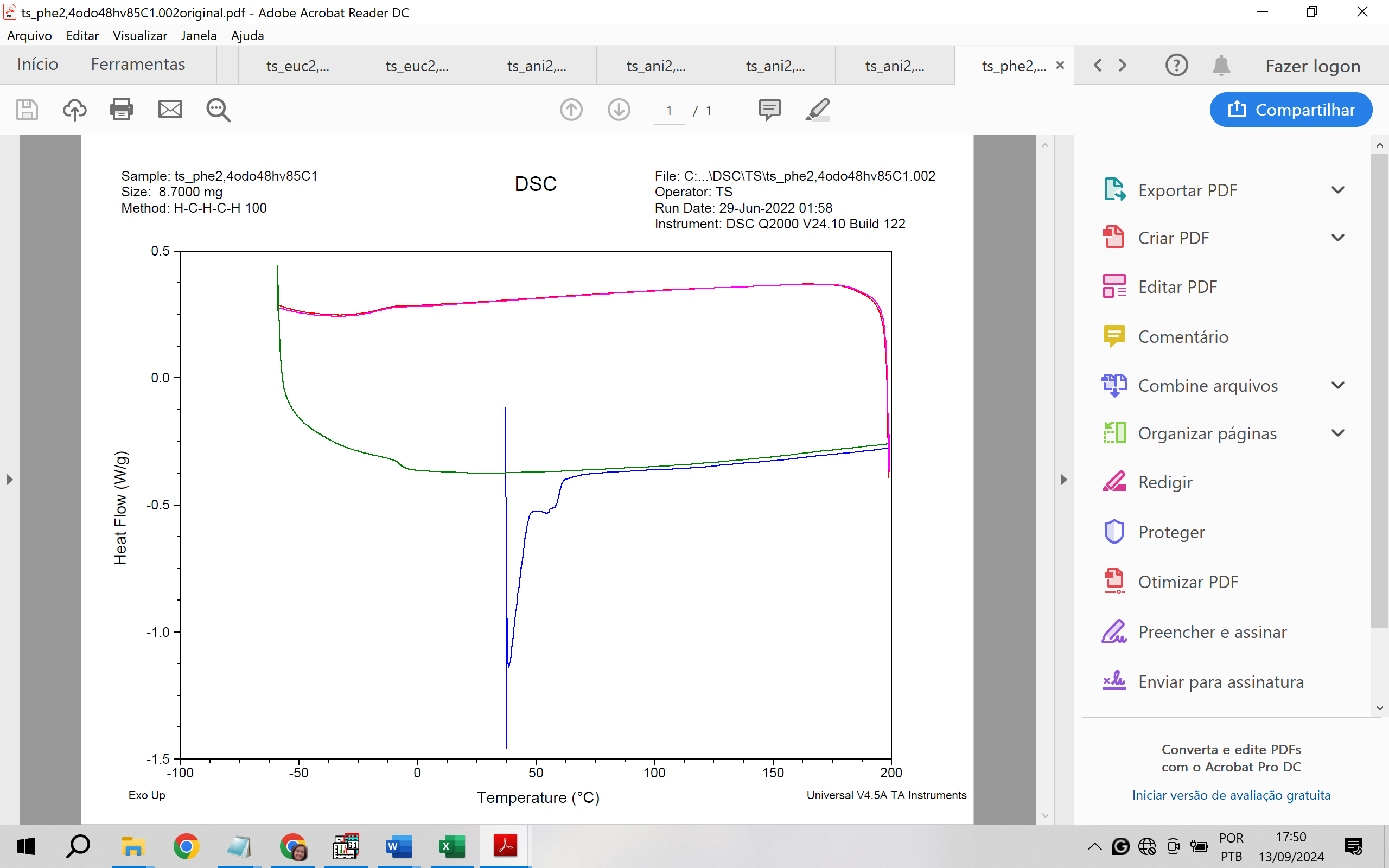


**Supplementary Figure 37.** DSC thermogram of the polymer PD24-ODO synthesised in phenetole as a solvent after 48 h reaction (6 h at 1000 mbar + 42 h at 360 mbar) of reaction.


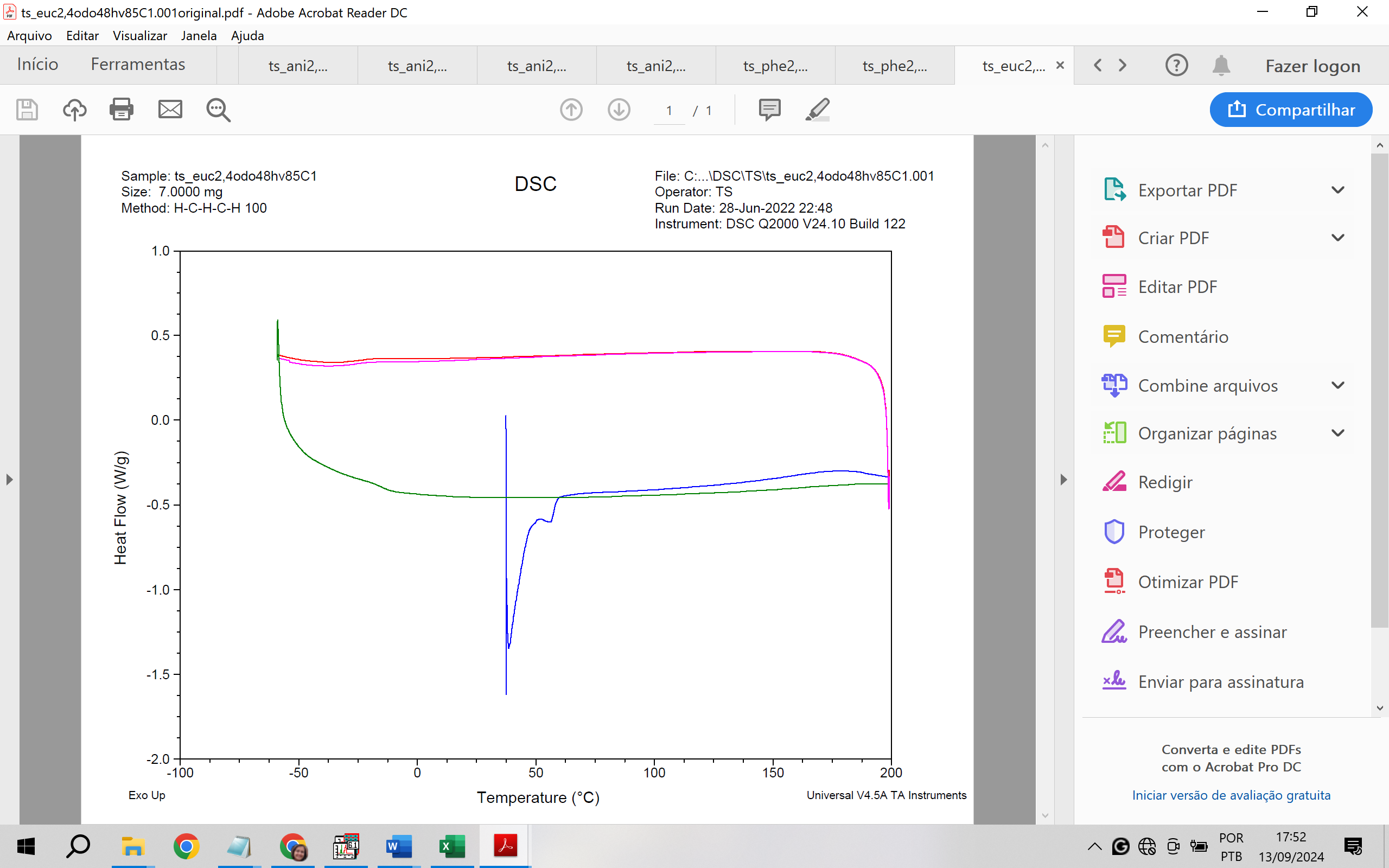


**Supplementary Figure 38.** DSC thermogram of the polymer PD24-ODO synthesised in eucalyptol as a solvent after 48 h (6 h at 1000 mbar + 42 h at 360 mbar) of reaction.


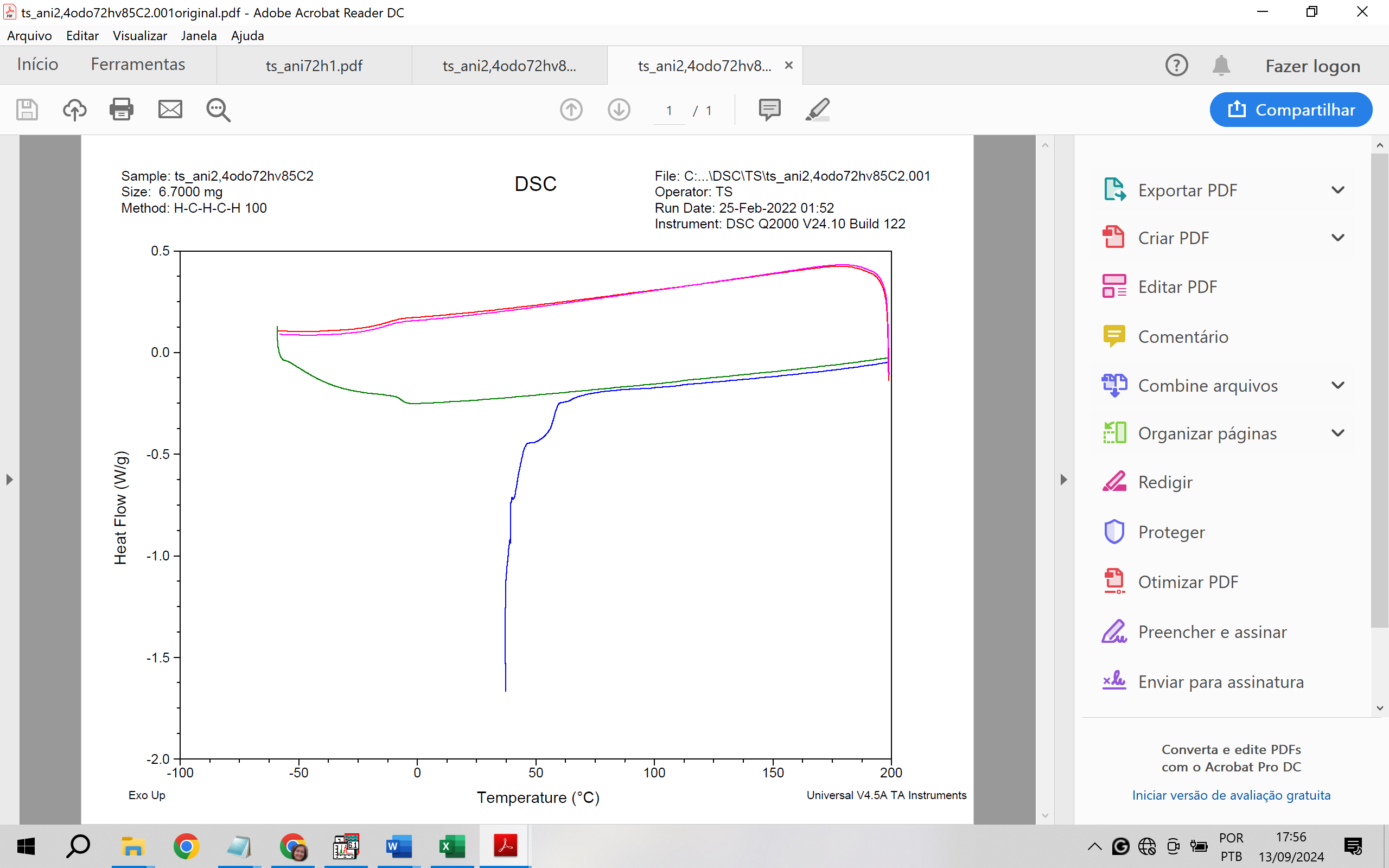


**Supplementary Figure 39.** DSC thermogram of the polymer PD24-ODO synthesised in anisole as a solvent after 72 h (6 h at 1000 mbar + 66 h at 360 mbar) of reaction.


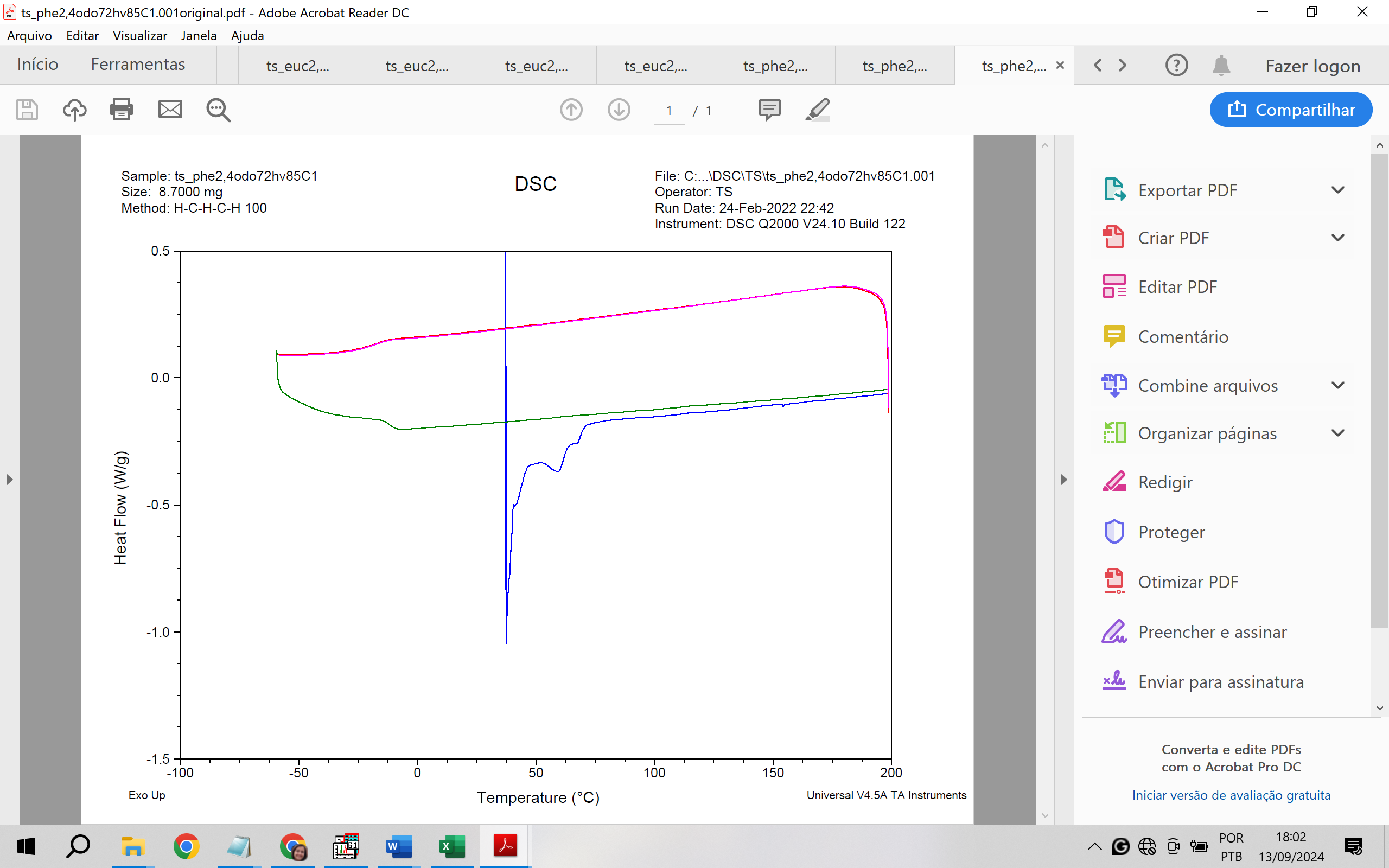


**Supplementary Figure 40.** DSC thermogram of the polymer PD24-ODO synthesised in phenetole as a solvent after 72 h (6 h at 1000 mbar + 66 h at 360 mbar) of reaction.


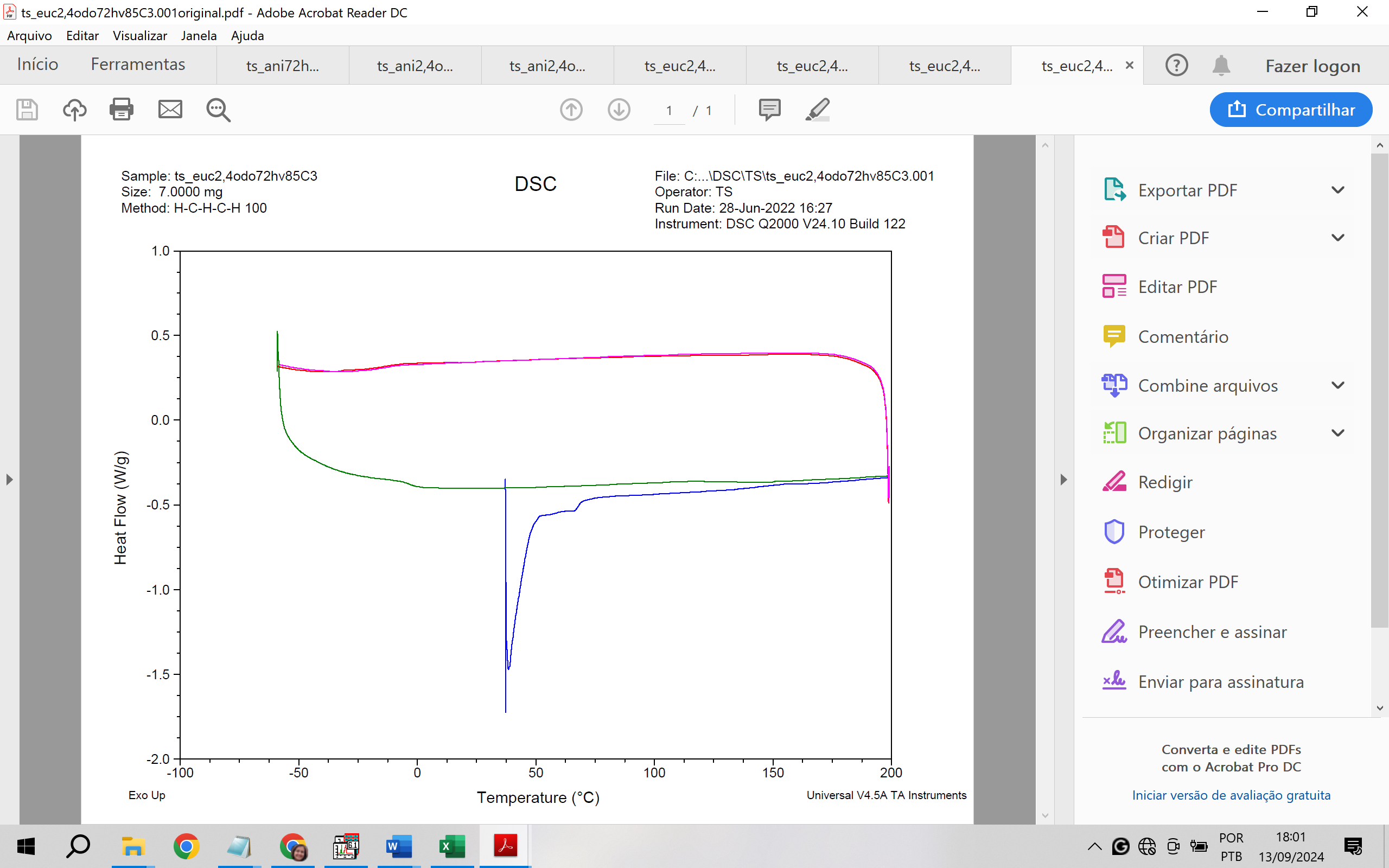


**Supplementary Figure 41.** DSC thermogram of the polymer PD24-ODO synthesised in eucalyptol as a solvent after 72 h (6 h at 1000 mbar + 66 h at 360 mbar) of reaction.


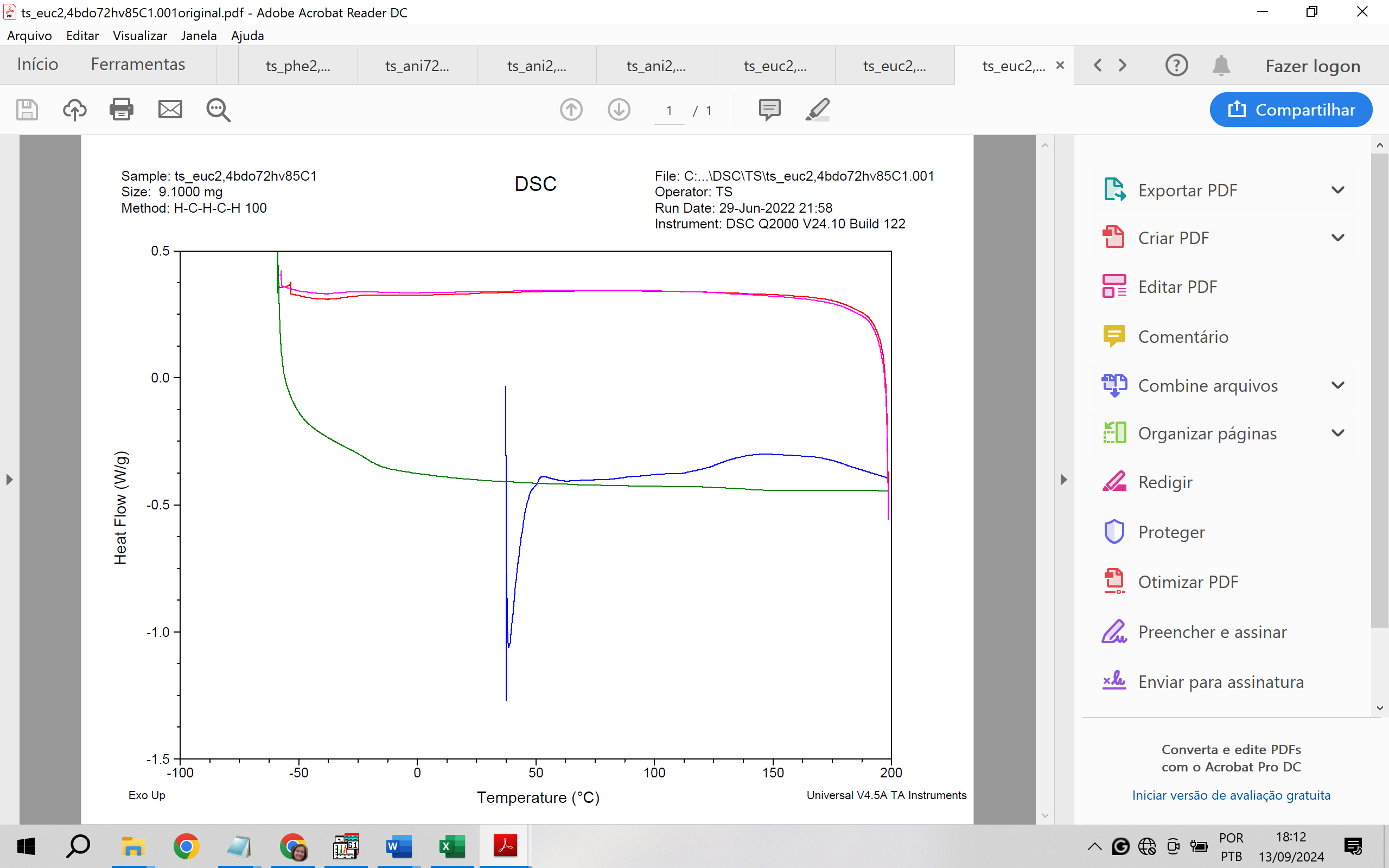


**Supplementary Figure 42.** DSC thermogram of the polymer PD24-BDO synthesised in eucalyptol as a solvent in 72 h (6 h at 1000 mbar + 66 h at 360 mbar).


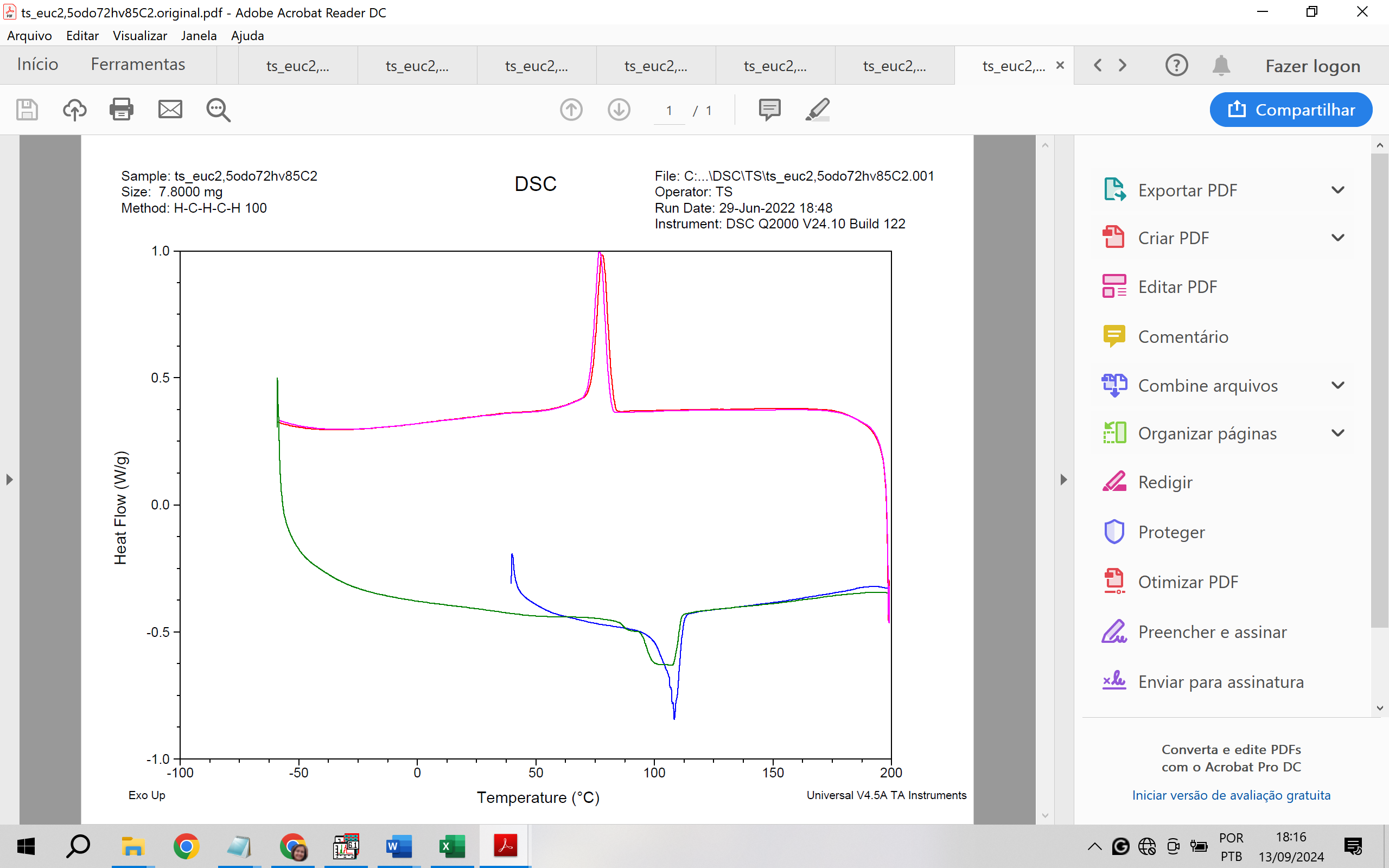


**Supplementary Figure 43.** DSC thermogram of the polymer PD25-ODO synthesised in eucalyptol as a solvent in 72 h (6 h at 1000 mbar + 66 h at 360 mbar).


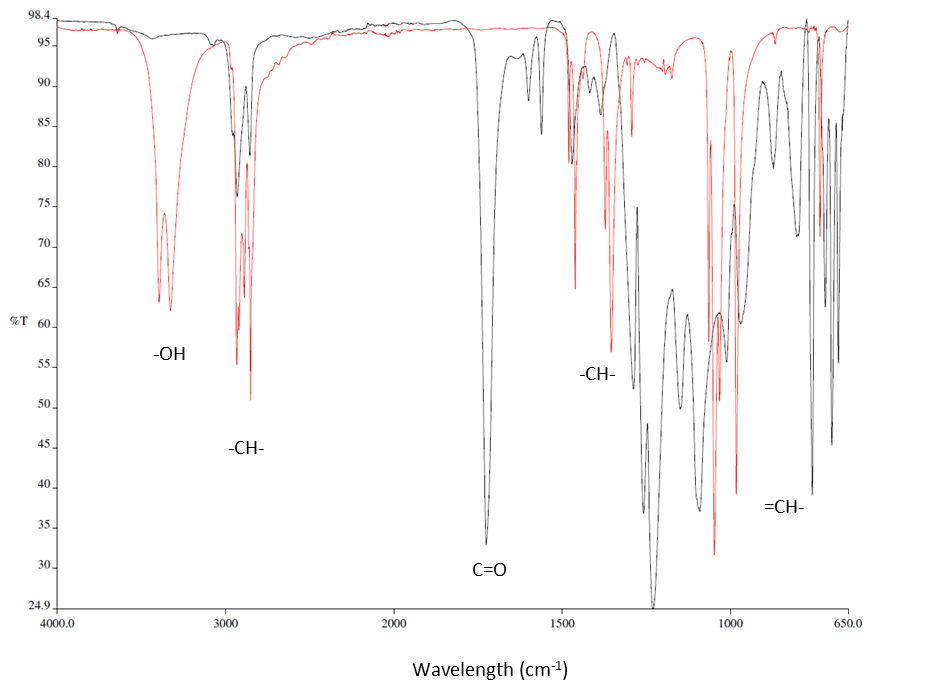


**Supplementary Figure 44.** Overlayed FT-IR of ODO (red) and of the polymer PD24-ODO (black) synthesized in chloroform as a solvent.


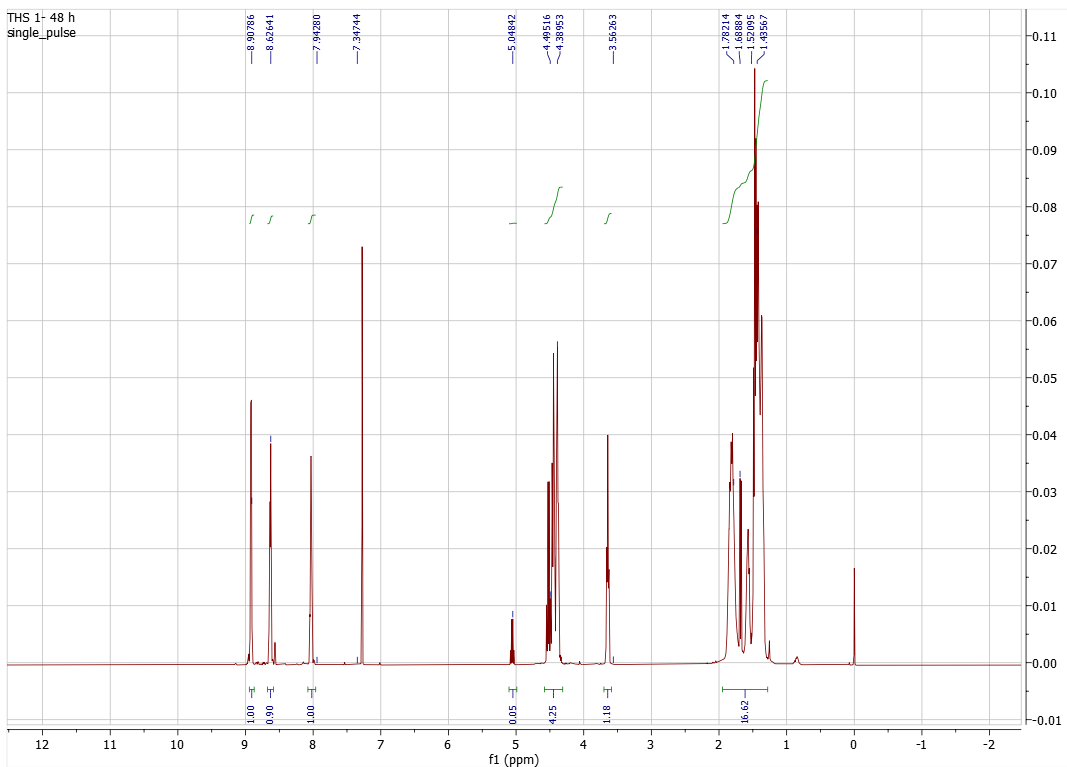
**Supplementary Figure 45.** ^1^H-NMR spectrum of the polymer PD24-BDO synthesized in toluene as a solvent after 48 h (6 h at 1000 mbar + 42 h at 360 mbar) of reaction.


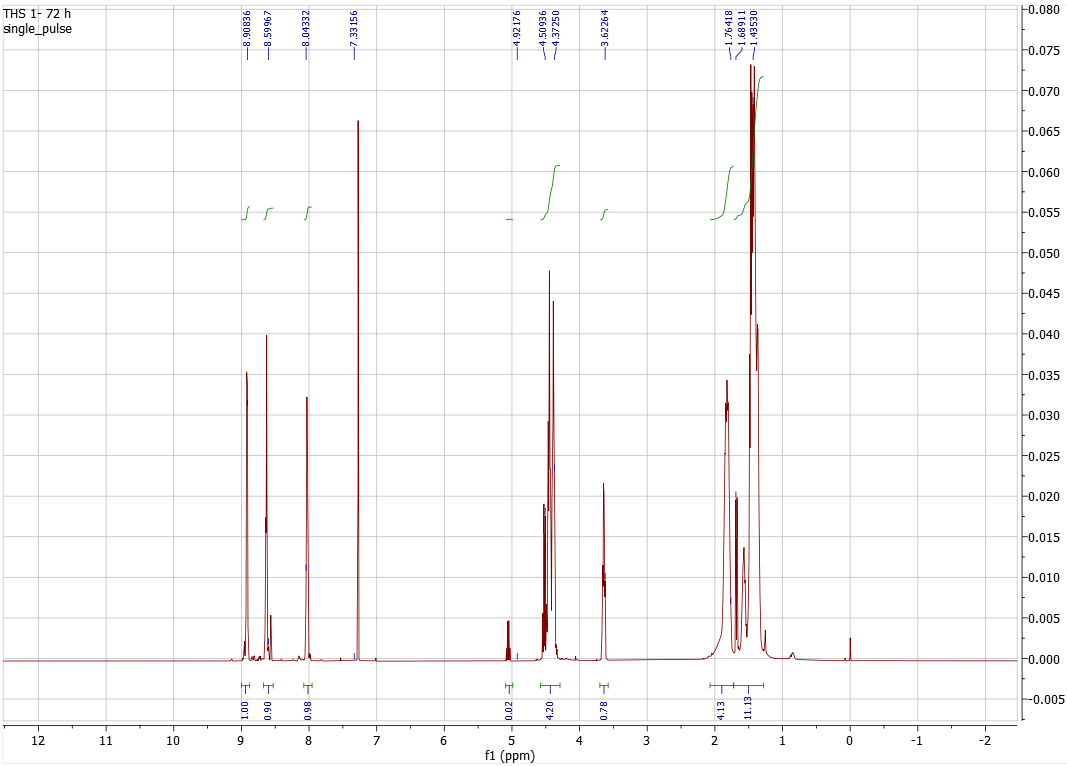


**Supplementary Figure 46.** ^1^H-NMR spectrum of the polymer PD24-ODO synthesized in toluene as a solvent after 72 h (6 h at 1000 mbar + 66 h at 360 mbar) of reaction.


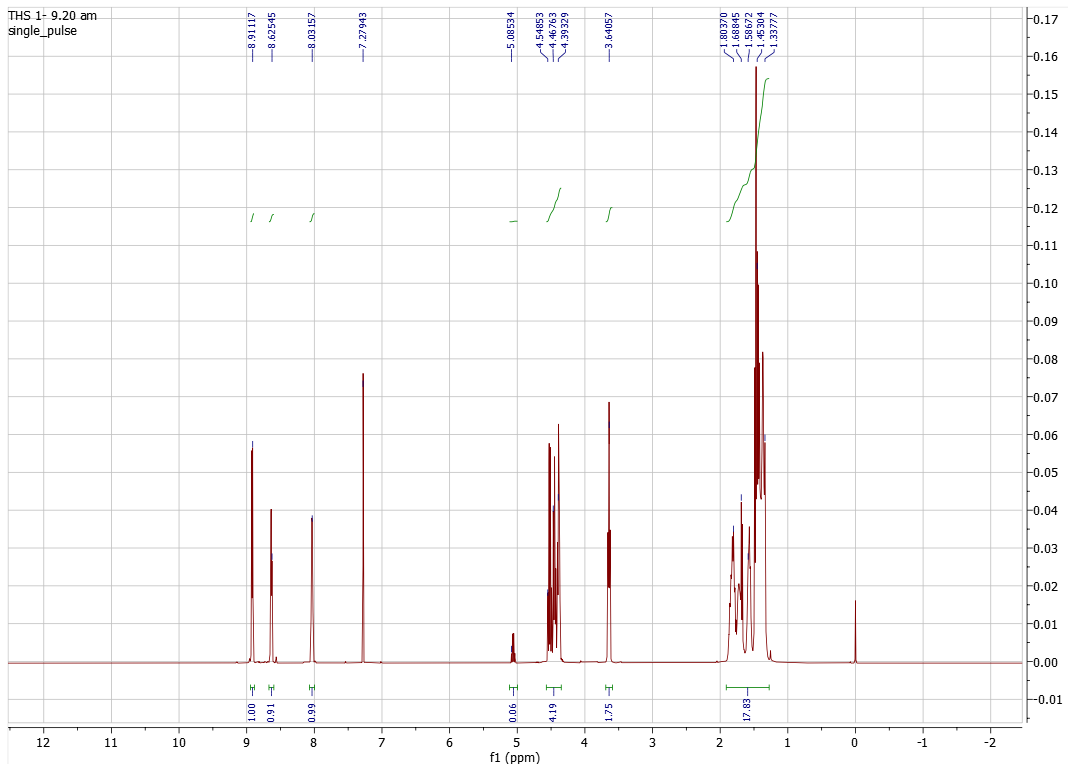
**Supplementary Figure 47.** ^1^H-NMR spectrum of the polymer PD24-ODO synthesized in toluene as a solvent after 96 h of reaction.

**Supplementary Tables**

| **Supplementary Table 1.** Price, hazard and toxicity comparison of the various solvents used in this work. | | | | | | |
| --- | --- | --- | --- | --- | --- | --- |
| **Solvent** | **Main hazards** | **Pictograms** | **Hazard statements** | **Precautionary statements** | **Lethal dose or concentration (LD, LC)** | **Price**  **[€/L]*** |
| Toluene | Highly flammable | 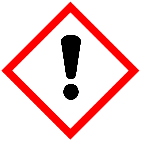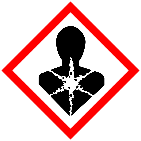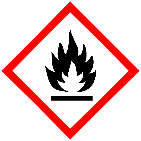 | H225, H304, H315, H336, H361d, H373 | P210, P240, P301+P310, P302+P352, P308+P313, P314, P403+P233 | LC_50_ >26700 ppm (rat, 1 h), 400 ppm (mouse, 24 h)  LC_Lo_ 55,000 ppm (rabbit, 40 min) | 45 |
| Cyclohexanone | - | 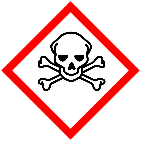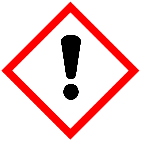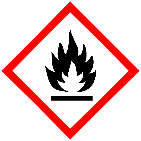 | H226, H302, H305, H312, H315, H318, H332 | P280, P305+P351+P338 | LC_50_ 8000 ppm (rat, 4 hr)  LC_Lo_ 4706 ppm (mouse, 1.5 hr) | 60 |
| Phenetole | - | 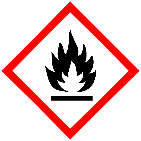 | H226 | - | LD50 Oral, Mouse, 2.200 mg/kg  LC_50_ and LC_Lo_ data not available | 140 |
| Anisole | - | 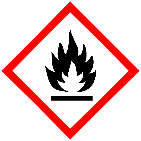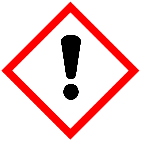 | H226, H315, H319 | P210, P233, P240, P241, P242, P243, P264, P280, P302+P352, P303+P361+P353, P305+P351+P338, P321, P332+P313, P337+P313, P362, P370+P378, P403+P235, P501 | LD50 Oral, Rat, 3700 mg/kg  LC_50_ and LC_Lo_ data not available | 65 |
| Eucalyptol | - | 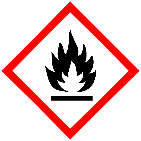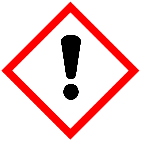 | H226, H304, H315, H317, H319, H411 | P210, P233, P240, P241, P242, P243, P261, P264, P272, P273, P280, P301+P310, P302+P352, P303+P361+P353, P305+P351+P338, P321, P331, P332+P313, P333+P313, P337+P313, P362, P363, P370+P378, P391, P403+P235, P405, P501 | LC50 - Oncorhynchus mykiss (rainbow trout) - 57 mg/l - 96 h  EC50 - Daphnia magna (Water flea) - > 100 mg/l - 48 h | 250 |
| ***** Price calculated considering average research laboratory prices of synthesis-grade reagents with a purity >99%. | | | | | | |

**Supplementary Table 2.** DSC analysis of the polyesters synthesized in different solvents.

| **Solvent** | **Diester** | **Diol** | **Reaction time (h)** | **Tg (°C)** | **ΔCp J/(g °C)** | **Melting temp. (°C)** |  |
| --- | --- | --- | --- | --- | --- | --- | --- |
| Anisole | PD24 | ODO | 96 | -26.76 | 0.26 | - |  |
|  |  |  |  | -35.5 | 0.28 | - |  |
|  | PD24 | ODO | 48 | -12.85 | 0.35 | - |  |
|  |  |  |  | -9.75 | 0.63 | - |  |
|  | PD24 | ODO | 72 | -6.27 | 0.37 | - |  |
|  |  |  |  | -6.72 | 0.35 | - |  |
| Phenetole | PD24 | ODO | 96 | -21.38 | 0.13 | - |  |
|  |  |  |  | -22.82 | 0.14 | - |  |
|  | PD24 | ODO | 48 | -6.94 | 0.32 | - |  |
|  |  |  |  | -10.83 | 0.30 | - |  |
|  | PD24 | ODO | 72 | -11.44 | 0.28 | - |  |
|  |  |  |  | -4.76 | 0.33 | - |  |
| Eucalyptol | PD24 | ODO | 96 | -35.55 | 0.57 | - |  |
|  |  |  |  | -40.25 | 0.52 | - |  |
|  | PD24 | ODO | 48 | -6.99 | 0.39 | - |  |
|  |  |  |  | -17.81 | 0.29 | - |  |
|  | PD24 | ODO | 72 | -7.57 | 0.26 | - |  |
|  |  |  |  | -3.97 | 0.32 | - |  |
|  | PD24 | BDO | 72 | -22.67 | 0.44 | - |  |
|  |  |  |  | -23.67 | 0.60 | - |  |
|  | PD25 | BDO | 72 | Not analysed (very low molecular weight) | | |  |
|  |  |  |  |  |  |  |  |
|  | PD25 | ODO | 72 | - | - | 92 |  |
|  |  |  |  | - | - | 93 |  |

**Supplementary Table 3.** TGA analysis of the polyesters synthesized in different solvents.

| Solvent | Diester | Diol | Reaction time (h) | Td_5_ (°C) | Td_10_ (°C) | Td_50_ (°C) |
| --- | --- | --- | --- | --- | --- | --- |
| Anisole | PD24 | ODO | 96 | 208 | 250 | 361 |
|  |  |  |  | 216 | 259 | 366 |
|  | PD24 | ODO | 48 | 324 | 341 | 371 |
|  |  |  |  | 308 | 329 | 368 |
|  | PD24 | ODO | 72 | 297 | 324 | 367 |
|  |  |  |  | 321 | 338 | 369 |
| Phenetole | PD24 | ODO | 96 | 214 | 258 | 360 |
|  |  |  |  | 230 | 277 | 363 |
|  | PD24 | ODO | 48 | 332 | 345 | 373 |
|  |  |  |  | 326 | 341 | 371 |
|  | PD24 | ODO | 72 | 324 | 339 | 370 |
|  |  |  |  | 326 | 339 | 370 |
| Cyclohexanone | PD24 | ODO | 96 | 144 | 172 | 318 |
|  |  |  |  | 143 | 167 | 329 |
|  | PD24 | ODO | 48 | 109 | 161 | 332 |
|  |  |  |  | 155 | 174 | 337 |
|  | PD24 | ODO | 72 | 174 | 203 | 352 |
|  |  |  |  | 172 | 201 | 346 |
| Eucalyptol | PD24 | ODO | 96 | 196 | 233 | 355 |
|  |  |  |  | 160 | 210 | 355 |
|  | PD24 | ODO | 48 | 288 | 323 | 366 |
|  |  |  |  | 241 | 306 | 364 |
|  | PD24 | ODO | 72 | 294 | 328 | 367 |
|  |  |  |  | 274 | 318 | 365 |
|  | PD24 | BDO | 72 | 213 | 244 | 328 |
|  |  |  |  | 207 | 237 | 327 |
|  | PD25 | BDO | 72 | 184 | 214 | 324 |
|  |  |  |  | 198 | 223 | 328 |
|  | PD25 | ODO | 72 | 248 | 304 | 371 |
|  |  |  |  | 263 | 311 | 370 |
